# Design and Application of Mini‐libraries of miRNA Probes for an Efficient and Versatile miRNA‐mRNA Cross‐linking

**DOI:** 10.1002/chem.202101171

**Published:** 2021-06-04

**Authors:** Anna L. Malinowska, Artur Laski, Jonathan Hall

**Affiliations:** ^1^ Institute of Pharmaceutical Sciences Department of Chemistry and Applied Biosciences ETH Zurich Vladimir-Prelog-Weg 4 8093 Zurich

**Keywords:** convertible nucleoside, cross-linking, miR-CLIP, oligonucleotides, RNA

## Abstract

MicroRNAs constitute a class of endogenous, non‐coding RNAs that influence various processes within the cell. By base‐pairing to partially‐complementary sites located in the 3’ untranslated region of target messenger RNAs, microRNAs participate in post‐transcriptional regulation of the majority of human protein‐coding genes. Their dysregulation has been related to many pathological processes and diseases. Thus, an in‐depth understanding of the microRNA mechanisms of action is crucial. Here, we present a new concept of probe design to achieve an efficient and sequence‐independent miRNA‐mRNA cross‐linking. The new strategy is based on the utilization of a controlled mixture of probes for a chosen miRNA, in which a trioxsalen moiety is introduced at the *N* ^4^‐position of a selected cytidine through short oligoethylene glycol‐based linkers. *In vitro* photo‐cross‐linking experiments with mini‐libraries of probes for microRNAs of interest showed variable cross‐linking efficiencies, demonstrating a general applicability of the presented approach.

## Graphical abstract

**We describe a new class of miRNA probes** with photo‐active trioxsalen attached *via* oligoethylene glycol‐based linkers. These cross‐link to RNA targets *in vitro* with efficiencies dependent on sequence and linker‐length. By using controlled mixtures of probes, a general approach to efficient covalent target‐capture independent of miRNAs sequence is proposed.

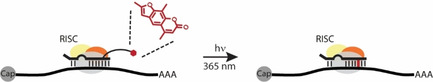

## Introduction

MicroRNAs (miRNAs) are endogenous, highly conserved RNAs acting as powerful regulators of a wide array of physiological processes in a variety of organisms.^[1]^ They bind to conserved sites in the 3’‐untranslated regions (3’‐UTRs) of target messenger RNAs (mRNAs) and induce their degradation^[2]^ or repress their translation.^[3]^ In canonical miRNA targeting, target recognition occurs by base‐pairing between the “seed” region (nucleotides 2–7 counting from the 5’‐end) of the miRNA and its mRNA target. However, miRNAs also participate in a wide variety of non‐canonical interactions, for example that involve regions other than their seeds,^[4]^ that contain bulged^[5]^ or mismatched pairing,^[6]^ by binding to regions outside the 3’‐UTR,^[7]^ or by binding to alternative classes of cellular RNAs.^[8]^ Alterations to miRNA expression patterns have been linked to numerous diseases including cardiovascular disorders,^[9]^ atherosclerosis,^[10]^ hepatitis C infections^[11]^ and cancer.^[12]^

**Figure 1:**
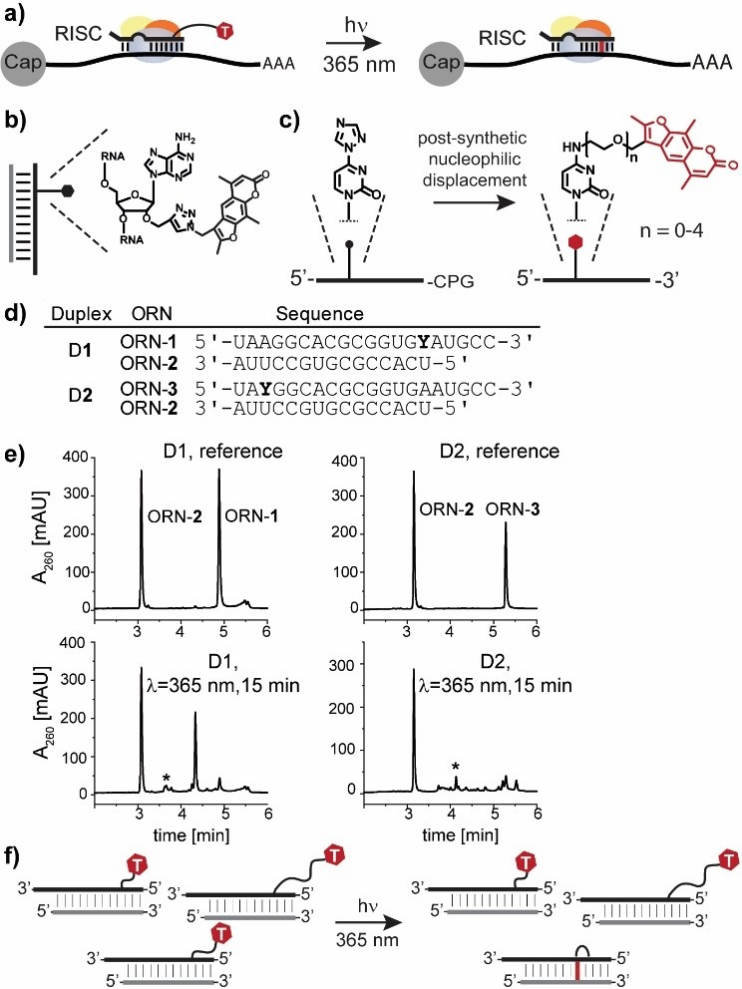
a) General scheme of the photo‐cross‐linking experiment with miRNA probes in miRISC; b) structure of adenosine modified with trioxsalen *via* CuAAC reaction; c) schematic representation of the triazole‐based convertible nucleoside approach for the functionalization of oligoribonucleotides with trioxsalen attached *via* linkers of different lengths; d) sequences of the miR‐124 probes (ORN‐**1** and ORN‐**3**) and their fully complementary counter‐strand (ORN‐**2**) tested in the *in vitro* photo‐cross‐linking experiments. **Y**: adenosine modified with trioxsalen attached at the 2’‐*O*‐position; e) RP‐HPLC chromatograms from the photo‐cross‐linking experiment with D**1** and D**2**. Top panels present the non‐irradiated samples (references). Lower panels represent samples irradiated for 15 min (λ=365 nm). Peaks designated with an asterisk (*) correspond to the cross‐linked duplex; D**1**: mass calc. [M+K]: 11493.3, mass found: 11491.4 and D**2**: mass calc.: 11452.3, mass found: 11452.8; f) schematic representation of the photo‐cross‐linking experiment with a mixture of miRNA probes in which the trioxsalen is attached at cytidines *via* linkers of different lengths.

In order to clarify the functions of a miRNA, new methods are needed to identify its targetome in cells. In the past few years, various computational and experimental approaches have been employed to identify the targetomes of miRNAs (reviewed in reference [13]). Our laboratory introduced the miRNA cross‐linking and immunoprecipitation (miR‐CLIP) method, which employs chemically‐synthesized miRNA probes, that are conjugated with photo‐reactive trioxsalen and biotin groups.^[8c]^ After transfection of the probe into cells, mild irradiation cross‐links the miRNA to its targets (Figure 1a). MiRNA‐mRNA products are isolated on streptavidin beads, and targets are identified by RNA sequencing. A strength of miR‐CLIP is that non‐canonical targets, i. e. new functions of miRNAs that are difficult to identify by other means, can be identified.[^8c^, ^14^]

Trioxsalen is a psoralen derivative, which is widely used for RNA cross‐linking, including miRNAs.^[15]^ It covalently cross‐links pyrimidines (preferentially uridines) in double‐stranded regions of RNA upon irradiation with UV light (λ=365 nm).^[16]^ In previous miR‐CLIP studies,[^8c^, ^14^] we prepared and tested miR‐CLIP probes for miR‐106a, miR‐132 and miR‐124. Additionally, miR‐106a and miR‐132 mono‐labelled analogues bearing trioxsalen at distinct sites were cross‐linked to short, complementary counter‐strands that mimic miRNA targets in the cell and were characterized using a dedicated *in vitro* photo‐cross‐linking HPLC‐assay. Trioxsalen was conjugated to the 2’‐*O*‐position of an adenosine *via* copper(I)‐catalysed azide‐alkyne cycloaddition (CuAAC; ‘click’) reaction (Figure 1b). We found that the efficiency of the cross‐linking reaction, which occurs through the minor groove in the miRNA/target duplex was variable and target‐sequence dependent; for some sequences, the data suggested that intra‐strand cross‐linking was a significant by‐product. We hypothesized that this was at least partially due to a paucity of accessible uridines in the target strands that could be reached by trioxsalen connected through a rigid triazole‐containing linker of defined length.

Here, we introduce a new strategy designed to capture miRNA targets in a sequence‐independent fashion. The approach employs probes in which trioxsalen is positioned in the major groove of a miRNA‐target duplex, with the cross‐linker conjugated *via* ethylene glycol‐based linkers of different lengths to cytidines (Figure 1c). We tested the approach using model probes of miR‐124 in the *in vitro* photo‐cross‐linking assays and found superior cross‐linking efficiencies compared to first‐generation probes prepared with the use of CLICK chemistry. Nevertheless, cross‐linking efficiency was dependent on linker length, and since it was not possible to predict *a priori* the ideal linker composition, we experimented with mixtures of probes bearing oligoethylene glycol‐linkers of different lengths, with the expectation that at least some library members would cross‐link sufficiently well to ensure efficient capture of the entire targetome. We validated the approach on different mini‐libraries of six miRNA probes. Moreover, the miR‐124 probes were accepted into the RNA‐induced silencing complex (RISC) and acted as miRNA mimics in a cellular reporter assay.

## Results and Discussion

First‐generation probes ORN‐**1** and ORN‐**3** (Figure 1d) were synthesized following the original protocol.^[8c]^ They were then tested for cross‐linking efficiency in the aforementioned *in vitro* assay. Thus, duplexes D**1** and D**2** were irradiated for 15 min (λ=365 nm), and cross‐linking yields were assessed using reverse‐phase HPLC (RP‐HPLC). Peaks with the lowest and the highest retention times corresponded to the unmodified counter‐strand (ORN‐**2**) and trioxsalen‐labelled probes (ORN‐**1**, ORN‐**3**), respectively, as verified by mass spectrometry (Figure 1e). Small amounts of cross‐linked products appeared on the chromatograms between the parent compounds, concomitant with the decrease in the peak intensities of the single‐strands. Side‐products of identical mass to the probe strand may have derived from intra‐strand cross‐linking.

**Figure 2:**
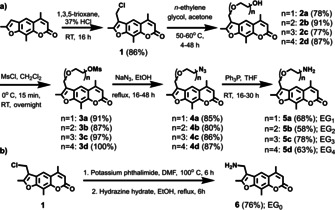
a) General synthetic scheme of trioxsalen derivatives with amino‐ethylene glycol linkers of various length (n=1‐4); b) synthesis of the 4’‐aminomethyltrioxsalen (n=0).

In the new probe design, ethylene glycol (EG)‐based linkers were employed for a cross‐linking reaction through the major groove of the miRNA‐target duplex. We have shown previously that small functional groups in the major groove do not significantly perturb the miRNA‐induced silencing complex (miRISC).^[17]^ We considered oligoethylene glycol groups as ideal linkers since they are flexible, are chemically‐inert and have been widely used in drug discovery, thanks to their advantageous pharmacokinetic properties.^[18]^ Trioxsalen analogues with amino‐linkers containing up to four ethylene glycol units were obtained following previously reported methods (Figure 2).^[19]^ Thus, trioxsalen was converted to the 4’‐chloromethyltrioxsalen (**1**),^[19a]^ and then substituted with the appropriate *n*‐ethylene glycol to produce **2 a**–**2 d** ^[19b]^ (Figure 2a). The hydroxyl groups were then mesylated and reacted with sodium azide to give **4 a**–**4 d**. In the final step, the azide derivatives were reduced to amines (**5 a**–**5 d**) with a Staudinger reaction. Purification of the amines was performed by acid‐base extraction. We found that for longer linkers (n=3, 4), extended reaction times were often required. Excess amounts of tri‐ and tetraethylene glycol were needed for quantitative conversions in the pegylation step, though the removal of the excess glycols required extensive aqueous washing steps and double purification by column chromatography. 4’‐Aminomethyltrioxsalen (**6**) was obtained from **1** by the Gabriel synthesis (Figure 2b).^[19c]^

The trioxsalen derivatives were conjugated at specific cytidines in the probe sequences using the “convertible nucleoside” approach^[20]^ (Figure 1c), which we have used previously for site‐specific functionalization of miRNA mimics.^[17]^ In this method, *O* ^4^‐triazolyluridine^[21]^ is incorporated into the oligonucleotide during solid‐phase synthesis. After synthesis of the full‐length sequence, but before oligonucleotide release from the solid support, the triazole leaving group is displaced by the amine‐bearing trioxsalen. Thus, *O* ^4^‐triazolyluridine phosphoramidite (**7**, Figure S1) was employed at position 6 of miR‐124 (ORN‐**4**, Table 1), and five equal portions of this solid support were then transferred into vials and treated with amines (**5 a**–**5 d**, **6**) in the presence of DBU. Reaction mixtures were shaken for 6 h at 60 °C, as these conditions appeared to be optimal in test reactions (data not shown). Reaction suspensions were then concentrated, and oligonucleotides were deprotected and detritylated under standard conditions. The RP‐HPLC purification resulted in the clean separation of the products, although for some of the compounds more than one purification round was needed. In total, five ORN‐**4** homologues modified at the *N* ^4^‐position of cytidine C_6_ with a trioxsalen attached through EG_0_‐EG_4_ linkers were obtained (ORN‐**4a** to ORN‐**4e**, Table 1, Table S1).

**Table 1:** MiR‐124 (ORN‐**4**) analogues modified with trioxsalen, attached by ethylene glycol‐based linkers.

ORN	Sequence^[a,b]^	Mass calc.	Mass obs.	*T*_M_^[c]^ (°C)	Δ*T*_M_^[d]^ (°C)
ORN‐**4**	UAAGGCACGCGGUGAAUGCC	6445	6445	76.9±0.3	–
ORN‐**4 a**	UAAGG**X_0_** ACGCGGUGAAUGCC	6685	6684	62.1±0.3	−14.8
ORN‐**4 b**	UAAGG**X_1_** ACGCGGUGAAUGCC	6729	6728	78.1±0.3	1.2
ORN‐**4 c**	UAAGG**X_2_** ACGCGGUGAAUGCC	6773	6772	75.6±0.3	−1.3
ORN‐**4 d**	UAAGG**X_3_** ACGCGGUGAAUGCC	6817	6816	74.5±0.2	−2.4
ORN‐**4 e**	UAAGG**X_4_** ACGCGGUGAAUGCC	6862	6860	73.3±0.2	−3.6

[a] 5’ to 3’. [b] **X**: cytidine functionalized at the *N* ^4^‐position. The 0–4 subscript indicates the number of ethylene glycol units present in the linker. [c] Melting temperatures with ORN‐**2** (2 μM concentration of each strand of the duplex in 100 mM NaCl, 10 mM phosphate buffer and 0.1 mM Na_2_EDTA, pH 7.0). [d] Δ*T*_M_=*T*_M_ (modified ORN) – *T*_M_ (ORN‐**4**).

In order to confirm that the cross‐linker groups would not perturb hybridization of the probes to RNA targets, their thermal melting stabilities (*T*_M_’s) with a 15‐nt complementary sequence (ORN‐**2**) were measured (Table 1, Figure S2a). The experimentally obtained *T*_M_’s for the probes were in general slightly lower than that of the unmodified duplex (−3.6 °C for D**8**, comprising ORN‐**4e**). However, for duplex D**4** comprising the probe with the EG_0_ linker (ORN‐**4a**), the *T*_M_ value (62.1+/‐ 0.3 °C) was lower than that of the unmodified parent duplex D**3** by 15 °C. Such a significant drop implied that the base‐pairing at this position was inhibited.^[22]^ Previous studies describe that mono‐substitution on the exocyclic *N* ^4^‐amine of (deoxy)cytidine does not abolish Watson‐Crick base‐pairing in DNA and RNA helices,[^17^, ^20a^, ^22^, ^23^] if the appended residue protrudes away from the Watson‐Crick face, towards the major groove of a duplex.[^20a^, ^24^] In the case of ORN‐**4a**, the linker was possibly too short so that the trioxsalen residue disturbed the local duplex structure. Therefore, probes with EG_0_ linker were not prepared for other miRNAs in further studies. We also performed circular dichroism (CD) measurements on the probes (Figure S2b). A strong positive band at 260 nm, a negative band at 210 nm and a small local maximum at 225 nm were consistent with A‐form helical conformations similar to unmodified RNAs. Taken together, the biophysical data provided supporting evidence that the new probe design would not alter the conformation of a duplex, thereby helping ensure that it would not affect miRISC functions in cells.

Next, *in vitro* photo‐cross‐linking experiments were performed with single probes of the ORN‐**4** series to assess how they might perform in miR‐CLIP experiments. Each of the probes was annealed to its counter‐strand ORN‐**2** and irradiated (λ=365 nm) for 15 min. Samples were then examined by RP‐HPLC and masses of the isolated product peaks were analysed by LC–MS. The parent duplex D**3** served as a negative control to ensure that no radical‐based cross‐linking independent of trioxsalen occurred.

**Figure 3:**
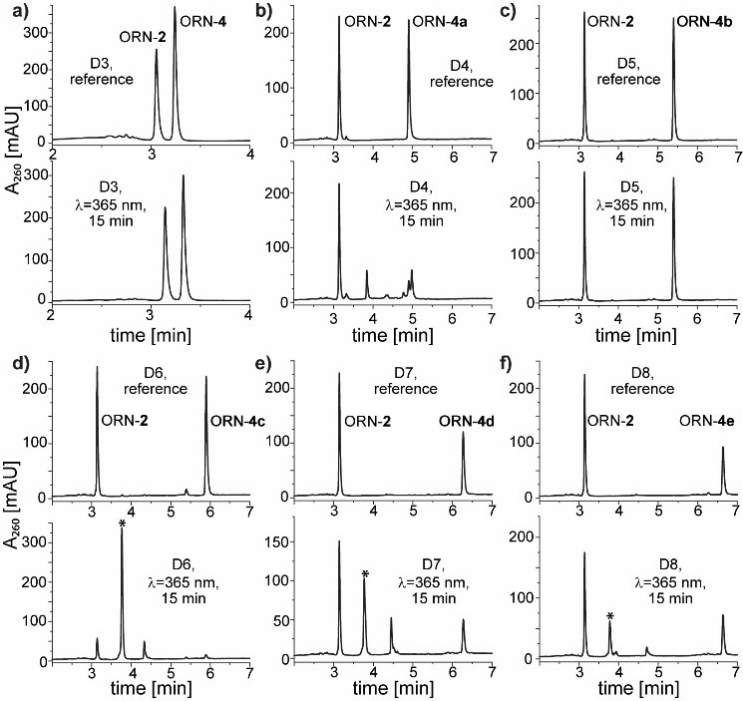
RP‐HPLC chromatograms from the *in vitro* photo‐cross‐linking experiments with miR‐124 probes from Table 1 and Table 2. Chromatograms are ordered according to increasing linker length (from EG_0_ to EG_4_). The top panels correspond to the non‐irradiated samples (references), lower panels represent samples irradiated for 15 min (λ=365 nm). Unmodified duplex D**3** serves as a negative control. Peaks marked with an asterisk (*) correspond to the cross‐linked duplexes; D**6**: mass calc.: 11461.3, mass found: 11459.6; D**7** mass calc.: 11505.3, mass found: 11503.6; D**8**: mass calc.: 11549.4, mass found: 11547.8.

Pleasingly, clean cross‐linking was observed for duplexes D**6**, D**7** and D**8**. The cross‐linking was most efficient for the probe with an EG_2_ linker (ORN‐**4c**, D**6**; Figure 3d, Table 2), which appeared to be almost quantitative on the chromatogram. Cross‐linking efficiency gradually decreased with the increased length of the linker, achieving ∼34 % and ∼24 % for D**7** and D**8** with EG_3_ and EG_4_ linkers, respectively (Figure 3e–f, Table 2).

**Table 2:** Photo‐cross‐linking experiments performed with ORN‐**4** homologues. The seed region of ORN‐**4** (unmodified miR‐124) is shown underlined.

#^[a]^	ORN	Sequence^[b]^	Mass calc.	CL^[c]^ product (yield)^[d]^
D3	ORN‐4 ORN‐2	5’‐UAAGGCACGCGGUGAAUGCC‐3’ 3’‐AUUCCGUGCGCCACU‐5’	11133	n.d.
D4	ORN‐4a ORN‐2	5’‐UAAGGX_0_ACGCGGUGAAUGCC‐3’ 3’‐AUUCCGUGCGCCACU‐5’	11373	n.d.
D5	ORN‐4b ORN‐2	5’‐UAAGGX_1_ACGCGGUGAAUGCC‐3’ 3’‐AUUCCGUGCGCCACU‐5’	11417	n.d.
D6	ORN‐4c ORN‐2	5’‐UAAGGX_2_ACGCGGUGAAUGCC‐3’ 3’‐AUUCCGUGCGCCACU‐5’	11461	Detected (∼76 %)
D7	ORN‐4d ORN‐2	5’‐UAAGGX_3_ACGCGGUGAAUGCC‐3’ 3’‐AUUCCGUGCGCCACU‐5’	11505	Detected (∼34 %)
D8	ORN‐4e ORN‐2	5’‐UAAGGX_4_ACGCGGUGAAUGCC‐3’ 3’‐AUUCCGUGCGCCACU‐5’	11549	Detected (∼24 %)

[a] Duplex entry. [b] **X** is trioxsalen‐substituted cytidine; subscript (0‐4) indicates the number of ethylene glycol units in the linker. [c] CL: cross‐linked. [d] Duplexes (3 μM concentration) were irradiated for 15 min at λ=365 nm. Approximate cross‐linking yields were calculated from peak intensities based on the consumption of the counter‐strand (ORN‐**2**) in the irradiated samples in comparison with the non‐irradiated samples. n.d.: undetected product.

The cross‐linking efficiencies observed for the probes carrying the modification at the *N* ^4^ position were significantly higher in comparison to those with the trioxsalen attached at the 2’‐*O*‐position of the same cytidine C_6_ (data not shown). This might have been due to shifting the modification from the minor to the major groove, and/or the higher flexibility of the linker chains. Both of these significantly affect the reach of the trioxsalen, possibly facilitating intercalation and subsequent cross‐linking. Nevertheless, we observed substantial differences in the cross‐linking efficiency as a function of linker length, with EG_2_ being the most effective reagent for the miR‐124 sequence modified at C_6_. Since C_6_ is located in the seed region of miR‐124, all canonical mRNA targets of miR‐124 share the same sub‐sequence in the immediate region of the expected cross‐linking. However, it was apparent that for non‐canonical miR‐124 targets, or a change to a different miRNA, the cross‐linking profile of the probes with oligoethylene glycol linkers would be altered. Furthermore, we had no easy means to predict which linker lengths might provide the most efficient cross‐linking yields. To help circumvent this constraint, we proceeded to investigate the use of a controlled mixture of probes, i. e. with linkers of different lengths. Based on the similar melting temperatures of ORN‐**4b** to ORN‐**4e**, we hypothesized that most of the probes would hybridize equally well to miR‐124 targets in cells creating a heterogeneous population of cross‐linked duplexes that differ only in the lengths of the trioxsalen linkers (represented schematically in Figure 1f). Thereafter, irradiation would be expected to produce a mixture of cross‐linked products with varying composition, all of which would serve equally well to identify the RNA targets of the miRNA in the miR‐CLIP protocol.

**Figure 4:**
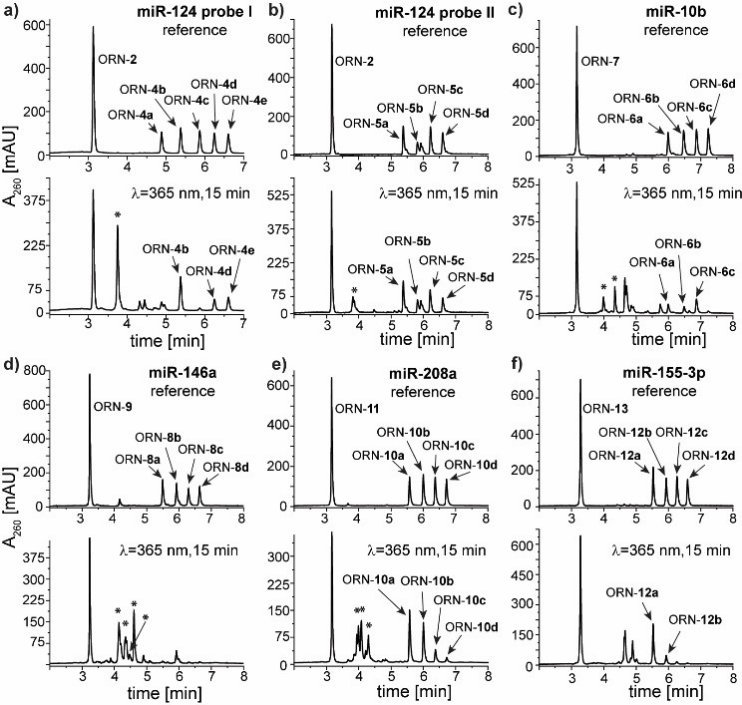
RP‐HPLC chromatograms from the *in vitro* photo‐cross‐linking experiments with an equimolar mixture of probes for a) miR‐124, b) miR‐124, c) miR‐10b, d) miR‐146a, e) miR‐208a, f) miR‐155‐3p with trioxsalen attached at the *N* ^4^ position of cytidine *via* linkers of different lengths. Mixtures consist of probes with linkers containing 0–4 EG units (ORN‐**4a** to ORN‐**4e**) or 1–4 EG units (all other series of probes). Irradiation time: 15 min (λ=365 nm). The concentration of each probe: 2 μM, concentration of the counter‐strand: 10 μM (a) or 8 μM (b‐f). Peaks indicated with an asterisk (*) correspond to the cross‐linked duplexes (masses of the isolated peaks are presented in the Supporting Information, section IV) (in many cases, probes with ethylene glycol‐based linkers were associated with abundant salt adducts in LC–MS analysis).

Therefore, equimolar (2 μM) amounts of ORN‐**4a** to ORN‐**4e** were mixed with the counter‐strand (ORN‐**2**; 10 μM) and irradiated. Chromatograms for the samples prior to irradiation (Figure 4a, top panel) showed a clean separation of the individual oligonucleotides. Post irradiation, the deconvoluted mass of the main newly‐formed peak (marked with an asterisk in Figure 4a) matched the expected masses of the cross‐linked duplexes D**6** and D**7**. Concomitantly, the intensity of the peak corresponding to ORN‐**4c** (containing EG_2_ linker) decreased. The intensities of ORN‐**4d** and ORN‐**4e** were also moderately reduced, reflecting the lower cross‐linking efficiency of these probes, in alignment with the data of Figure 3 and Table 2. The data confirmed that the application of the probes as a mixture was not detrimental to cross‐linking efficiency.

In order to determine whether such promising results could be also obtained when the trioxsalen is conjugated at other positions in miR‐124, we prepared a second set of probes, in which the oligoethylene glycol linkers were moved from C_6_ to C_8_ (ORN‐**5a** to ORN‐**5d**). Purities of the oligonucleotides in the ORN‐**5** series were similar to the purities of the members of the ORN‐**4** series (Table S1). ORN‐**5a** to ORN‐**5d** were annealed with ORN‐**2** and subjected to photo‐cross‐linking in an analogous fashion to ORN‐**4a** to ORN‐**4e**. Once again, the shifted retention times in the RP‐HPLC chromatogram, consumption of the counter‐strand and the mass analysis of the newly‐formed peaks were consistent with duplex cross‐linking (Figure 4b), but because of the presence of various ionic adducts in the MS deconvolution spectra, we were unable to identify which of the individual probes within the mixture had reacted preferentially (deconvoluted masses from the cross‐linking experiments are presented in the Supporting Information, section IV).

To expand the analysis to different miRNAs, we prepared an analogous set of four probes each for miR‐10b, miR‐146a, miR‐208a and miR‐155‐3p (Table 3, Table S1). The purities of the synthesized oligoribonucleotides were with a few exceptions higher than 95 % (Table S1). The new probes were also irradiated as mixtures using the *in vitro* assay in an analogous fashion to that of miR‐124. Cross‐linked products were identified based on their masses and their retention times, and showed a range of reactivity profiles. Specifically, duplexes comprising miR‐10b probes yielded cross‐linking products mostly from the EG_3_ and EG_4_ linkers (ORN‐**6c** to ORN‐**6d**, resp.; Figure 4c), which were confirmed unambiguously by their masses. MiR‐146a probes comprising EG_2_, EG_3_ and EG_4_ linkers (ORN‐**8b** to ORN‐**8d**) cross‐linked to ORN‐**9** with almost complete consumption of the probes (Figure 4d), whereas for miR‐208a, cross‐linking to ORN‐**11** was most efficient with the longer linker lengths, i. e., with ORN‐**10c** and ORN‐**10d** (Figure 4e).

**Table 3:** Sequences of probes used in the *in vitro* photo‐cross‐linking experiments as controlled mixtures.

miRNA	ORN	Sequence (5′ to 3′)^[a,b]^
miR‐124	ORN‐**4 a–e** ORN‐**2**	5′‐UAAGG**X_n_** ACGCGGUGAAUGCC‐3′ 3′‐AUUCCGUGCGCCACU‐5′
miR‐124	ORN‐**5 a–d** ORN‐**2**	5′‐UAAGGCA**X_n_** GCGGUGAAUGCC‐3′ 3′‐AUUCCGUGCGCCACU‐5′
miR‐10b	ORN‐**6 a–d** ORN‐**7**	5′‐UAC**X_n_** CUGUAGAACCGAAUUUGUG‐3′ 3′‐AUGGGACAUCUUGGC‐5′
miR‐146a	ORN‐**8 a–d** ORN‐**9**	5′‐UGAGAA**X_n_** UGAAUUCCAUGGGUU‐3′ 3′‐ACUCUUGACUUAAGG‐5′
miR‐208a	ORN‐**10 a–d** ORN‐**11**	5′‐AUAAGA**X_n_** GAGCAAAAAGCUUGU‐3′ 3′‐UAUUCUGCUCGUUUU‐5′
miR‐155‐3p	ORN‐**12 a–d** ORN‐**13**	5′‐CUCCUACAUAUUAG**X_n_** AUUAACA‐3′ 3′‐GAGGAUGUAUAAUCG‐5′

[a] **X**: cytidine with trioxsalen at the *N* ^4^ position; the subscript n indicates a mixture of probes with linkers containing 0–4 EG units (ORN‐**4** series) or 1–4 EG units (other oligoribonucleotide series). [b] Samples were irradiated for 15 min (λ=365 nm). The concentration of each probe: 2 μM; concentration of the counter‐strands: 10 (a) or 8 (b‐f) μM.

For miR‐155‐3p, trioxsalen was conjugated to position 15 of the sequence (ORN‐**12a** to ORN‐**12d**), i. e. outside of the seed region. In this example, two new faster migrating peaks were detected in the HPLC chromatogram, for which the mass analysis corresponded to starting material (Figure 4f). This was consistent with no capture of the complementary strand ORN‐**13**, but rather with intra‐strand cross‐linking events, possibly at dinucleotides U_11_U_12_ or U_17_U_18_ in ORN‐**12**. It should be noted that position‐15 is the last base‐pair of the ORN‐**12**/ORN‐**13** duplex, which may also have adversely influenced cross‐linking with proximal uridines, since the last base‐pair may be susceptible to duplex “breathing”.

Taken together, these results validate the concept of using controlled mixtures of probes with variable linker lengths to increase the overall cross‐linking efficiency and at least partly circumvent the sequence‐dependent covalent capture of target mRNA by miRNA probes.

**Figure 5:**
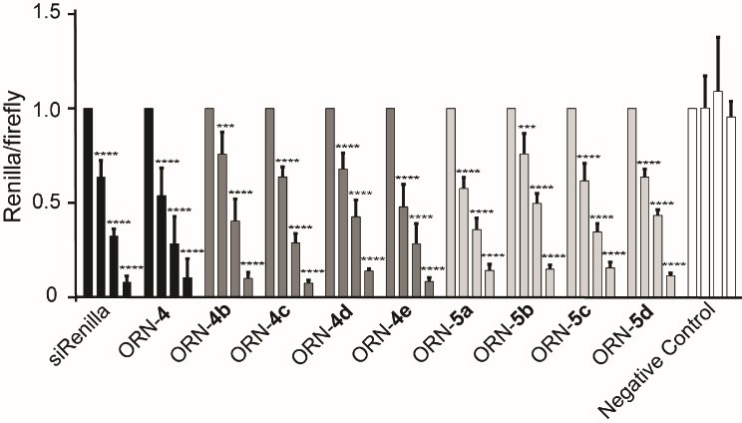
Performance of the trioxsalen‐modified miR‐124 probes, measured in a dual‐luciferase reporter assay. HEK293T cells were transfected with the fully complementary reporter plasmid and increasing concentrations (0, 2.5, 10 and 40 nM) of one of the following duplexes: siRenilla, unmodified miR‐124 duplex (ORN‐**4**), trioxsalen‐modified miR‐124 duplexes ORN‐**4b** to ORN‐**4e** and ORN‐**5a** to ORN‐**5d**, or a randomized negative control.^[26]^ A fully‐complementary counter‐strand (ORN‐**2**) was used as the passenger strand to create duplexes with ORN‐**4**, ORN‐**4b** to ORN‐**4e** and ORN‐**5a** to ORN‐**5d**. Significance calculated by 2‐way ANOVA followed by Dunnett's test: * (P≤0.05), ** (P≤0.01), *** (P≤0.001), **** (P ≤0.0001). N=3.

Having demonstrated the potential advantageous properties of using mini‐libraries of this new class of probes for miR‐CLIP experiments, we assessed their compatibility with the RNA interference mechanism in cells. It has been reported that the substitution of miRNA mimics with functional groups ‐ particularly in their seed regions ‐ can lead to their inactivation in miRNA mechanisms of silencing, presumably due to a failure to be taken into the miRISC.[^8c^, ^25^] To assess the functionality of the new probes, the miR‐124 probe series were tested in HEK293T cells using a reporter assay that we employed in the original miR‐CLIP study.^[8c]^ For this purpose, a fully complementary binding site to miR‐124 was cloned into the 3’‐UTR of the *Renilla* luciferase gene, encoded on a dual reporter plasmid. Next, HEK293T cells were co‐transfected with the plasmid and with duplexes containing probes (ORN‐**4b** to ORN‐**4e** and ORN‐**5a** to ORN‐**5d**), native miR‐124 (ORN‐**4**) and a positive control (siRNA targeting *Renilla* gene) or a negative control (randomized duplex).^[26]^ After readout, all of the trioxsalen‐bearing probes showed similar concentration‐dependent inhibition of reporter gene expression, with effects comparable to the positive controls (Figure 5). Probes with the modification at position C_6_ of the sequence (ORN‐**4** series) demonstrated slightly stronger target repression than probes substituted at C_8_ (ORN‐**5** series). These results provided confirmatory evidence that this new class of probe can be functional and incorporated into miRISC machinery in cells.

## Conclusion

In conclusion, we have presented a new strategy for the design and use of novel class of trioxsalen‐labelled probes conjugated to cytidines of a miRNA through oligoethylene glycol linkers using the convertible nucleoside approach. These probes cross‐linked *in vitro* to complementary synthetic targets with varying efficiencies depending on the length of the linker and the relative location of reacting uridines in the target strands. By employing an equimolar mixture of probes with different linkers for a given miRNA, the overall yields of successful cross‐linking could be maximized while at the same time, attenuating sequence‐dependent effects governed by the miRNA sequence. This strategy is therefore well‐suited to the miR‐CLIP method where all of the targets of a given miRNA can be captured and identified in cells. We validated the strategy on six model miRNAs, of which only one ‐ miR‐155‐3p ‐ failed to *produce* a cross‐linking reaction. Reasons for this were unclear, but may well have been related to the technical set‐up of the assay, i. e. it is possible that in a native environment, miR‐155‐3p probes (ORN‐**12** series) would also be effective. The convertible nucleoside approach is a very effective method to introduce modifications into the major groove of RNA duplexes. The versatility of our method can be increased further by adapting this method also to the *N* ^6^ position of adenosine. Experiments in this direction, as well as miR‐CLIP reagents using these probes are underway.

## Experimental Section

### General experimental details

Trioxsalen (CAS Number: 3902‐71‐4) was purchased from TCI. Thin‐layer chromatography (TLC) was done on silica gel 60 Å F254 aluminium sheets (Merck). Synthesized compounds were purified using the preparative flash column chromatography (Isolera One flash chromatography system, Biotage) which was carried out on silica gel, 60 Å (Fluka). Solvents for TLC, column chromatography, and extractions were commercial grade. NMR spectra were recorded on a Bruker Av400 at a resonance frequency of 400 MHz (for ^1^H NMR), 101 MHz (for ^13^C NMR) or 162 MHz (for ^31^P NMR). Solvent signals were used as internal standards. Chemical shifts (*δ*) are given in ppm. ^1^H NMR spectra are described as follows: multiplicity, the coupling constant *J* [Hz], the number of protons (integral). Multiplicity in the ^1^H NMR spectra is described as s=singlet, d=doublet, dd=doublet of doublets, dt=doublet of triplets, ddd=doublet of doublet of doublets, t=triplet and m=multiplet. For ^13^C and ^31^P NMR spectra, only the chemical shifts of signals are given. High‐resolution mass spectrometry (HR‐MS) was performed by the MS service of the Laboratory for Organic Chemistry at the ETH Zürich. The ESI‐HR‐MS spectra were recorded on a Bruker Daltonics maXis (ESI‐QTOF‐MS).

### Oligonucleotide synthesis

The sequences of the synthetic miRNAs were designed based on the miRNA sequences reported in the freely available miRBase database.^[27]^

### General information

Chemicals and solvents were purchased from Biosolve, Sigma‐Aldrich, VWR, Acros Organics and TCI. RNA phosphoramidites were purchased from Thermo Fisher Scientific. Oligoribonucleotides were synthesized on a 50 nmol or 1 μmol scale with the MM12 synthesizer (Bio Automation Inc.) using 500 Å UnyLinker Controlled Pore Glass (CPG) (ChemGenes). Cleavage of the DMTr group was performed with 3 % dichloroacetic acid in dichloromethane (DCM) (v/v). The RNA phosphoramidites were prepared as 0.08 M solutions in dry acetonitrile (ACN). 0.24 M solution of 5‐benzylthio‐1*H*‐tetrazole (Carbosynth) in dry ACN was used as an activator. Failed sequences were capped with a 1 : 1 mixture of CapA (acetic acid anhydride in tetrahydrofuran) and CapB (*N*‐methylimidazole and pyridine in tetrahydrofuran). The oxidizing step was performed with 0.02 M iodine in tetrahydrofuran/pyridine/water (7 : 2 : 1, v/v/v).

### Synthesis parameters

**50 nmol scale**: deblock 3×20 s, coupling times: 2×120 s for the standard RNA phosphoramidites and 2×180 s for the modified phosphoramidites, capping: 1×30 s, oxidation: 1×40 s.

**1 μmol scale**: deblock 2×40 s, coupling times: 1×300 s for the standard RNA phosphoramidites and 1×360 s for the modified phosphoramidites, capping: 1×60 s, oxidation: 1×60 s.

### Oligonucleotides: cleavage from the CPG, deprotection and purification

After the solid‐phase synthesis, the CPG with the synthesized oligoribonucleotides was treated with gaseous methylamine for 1.5 h at 70 °C (unmodified oligoribonucleotides) or with a mixture of 200 μL of ammonia solution (25 % in water) and 200 μL of methylamine solution (40 % in water) for 6 h at 35 °C (modified oligoribonucleotides). After basic deprotection and cleavage of the oligoribonucleotides from the solid support, the CPG was filtered and washed with 3×200 μL of water/EtOH (1 : 1) mixture. 20 μL of 1 N Tris‐base was added to the filtrate and it was evaporated in a SpeedVac (miVac duo SpeedVac, Genevac). Desilylation was carried out by the treatment of the oligoribonucleotides with 130 μL of a freshly prepared mixture of *N*‐methyl‐2‐pyrrolidone (NMP) (60 μL), triethylamine (30 μL) and triethylamine trihydrofluoride (Et_3_N×3HF) (40 μL) at 70 °C for 2 h. The reaction was quenched with trimethylethoxysilane (200 μL, 3 min, room temperature). Diethyl ether (200 μL, 5 min, room temperature) was then added and the mixture was vortexed and centrifuged.

In case of the 1 μmol scale synthesis, the CPG was divided into smaller portions (around 20 mg of the CPG each) and treated with a doubled amount of the reagents.

The supernatant was removed and the precipitate was dissolved in 200 μL of ultrapure water and purified on an Agilent 1200 series preparative RP‐HPLC on an XBridge OST C‐18 column (10×50 mm, 2.5 μm; Waters) at 65 °C with a flow rate 5 mL/min. Eluent A: 0.1 M aqueous triethylamine/acetic acid, pH 8.0; eluent B: 100 % ACN; gradient 10–50 % B in 5 min. Fractions containing the DMTr‐on product were collected, dried in a SpeedVac (miVac duo SpeedVac, Genevac) and treated with 200 μL of 40 % aqueous acetic acid for 15 min at room temperature. Samples were concentrated in a SpeedVac (miVac duo SpeedVac, Genevac), dissolved in 200 μL of ultrapure water and purified in DMTr‐off mode by RP‐HPLC on an XBridge OST C‐18 column (10×50 mm, 2.5 μm; Waters) at 65 °C with a flow rate 5 mL/min. Eluent A: 0.1 M aqueous triethylamine/acetic acid, pH 8.0; eluent B: 100 % ACN; gradient 2–20 % B in 6 min. The gradient was changed for samples with longer linkers (2‐22 % B in 8 min). Fractions containing the product were collected, dried in a SpeedVac (miVac duo SpeedVac, Genevac), re‐dissolved in 200 μL of ultrapure water and analysed by LC‐MS (Agilent 1200/6130 system) on an Acquity OST C‐18 column (2.1×50 mm; Waters). The column oven was set to 65 °C, flow‐rate: 0.3 mL/min. Eluent C: ultrapure water containing 0.4 M hexafluoroisopropanol (HFIP), 15 mM triethylamine; eluent D: 100 % MeOH; gradient 5–35 % D in 14 min (ORN‐**4**), 5–40 % D in 14 min (ORN‐**1**, ORN‐**2**, ORN‐**7**) or 5–50 % D in 14 min (all the other oligoribonucleotides).

UV‐absorption (λ=260 nm) of the final products was measured in duplicates (modified oligoribonucleotides) or triplicates (unmodified oligoribonucleotides) on a NanoDrop 2000 spectrophotometer (Fisher Scientific). Concentrations were calculated with in‐house programmed software. No next‐neighbour correction for the extinction coefficients of the modified bases was made (influence <10 %).

### Post‐synthetic triazole substitution

20 mg portions of the CPG with ORN‐**4**, ORN‐**5**, ORN‐**6**, ORN‐**8**, ORN‐**10** and ORN‐**12** modified with the *O* ^4^‐triazolyluridine were prepared in screw cap tubes. Amines (**5a**–**5d** or **6**; 10–18 mg/reaction, depending on the linker length) were dissolved in a mixture of 150 μL of dry ACN and 50 μL of DBU and added to the CPG. The suspension was then incubated for 6 h at 60 °C with shaking (ThermoMixer C, Eppendorf) and concentrated in the SpeedVac (miVac duo SpeedVac, Genevac). Further deprotection and purification was performed according to the standard procedure for the modified oligoribonucleotides (described above). Some samples required double purification by RP‐HPLC.

### *In vitro* photo‐cross‐linking assay

**The*****in vitro*****photo‐cross‐linking with single probes**: The *in vitro* photo‐cross‐linking assay was performed following the published protocols with minor changes.^[8c]^ Calculated volumes of the trioxsalen‐modified ORN and its unmodified counter‐strand were mixed, dried, and re‐dissolved in 200 μL of the phosphate buffer (2.5 mM Na_2_HPO4, 5 mM NaH_2_PO_4_, 100 mM NaCl and 0.1 mM Na_2_EDTA) so that the final concentration of each strand was 3 μM. For annealing, the solution was heated to 95 °C, held at that temperature for 5 min and cooled down to room temperature over 2 h. Samples were irradiated for 15 min (Bio‐Link BLX, Vilber. UV source: 5×8‐watt lamps, λ=365 nm, a distance of the plate from the lamp: 5 cm) in an open 24‐well plate placed on ice. Then, the samples were purified by RP‐HPLC [XBridge OST C‐18 column (10×50 mm, 2.5 μm; Waters) at 65 °C with a flow rate 5 mL/min] using a gradient 1–60 % D in 12 min. Collected fractions were dried (miVac duo SpeedVac, Genevac), re‐dissolved in ultrapure water and analysed by LC–MS [Acquity OST C‐18 column (2.1×50 mm; Waters). The column oven was set to 65 °C, flow‐rate: 0.3 mL/min] with a gradient 5–60 % D in 14 min. In case of some of the oligonucleotides modified with the ethylene glycol linkers, the analysis by LC–MS was made more difficult by the presence of salt adducts (usually sodium or potassium cations), especially in case of cross‐linked duplexes.

**The*****in vitro*****photo‐cross‐linking with a mixture of probes**: Samples were prepared according to the standard protocol described above with a small modification; calculated volumes (corresponding to the 2 μM concentrations) of the trioxsalen‐modified ORNs were taken and mixed with the calculated volume (corresponding to the 10 μM or 8 μM concentration) of the unmodified counter‐strands. The mixture was concentrated, re‐dissolved in 200 μL of the phosphate buffer (2.5 mM Na_2_HPO_4_, 5 mM NaH_2_PO_4_, 100 mM NaCl and 0.1 mM Na_2_EDTA), annealed and cooled down to room temperature. After irradiation, samples were analysed according to the standard procedure.

## Conflict of interest

The authors declare no conflict of interest.

## Supporting information

As a service to our authors and readers, this journal provides supporting information supplied by the authors. Such materials are peer reviewed and may be re‐organized for online delivery, but are not copy‐edited or typeset. Technical support issues arising from supporting information (other than missing files) should be addressed to the authors.

Supplementary
