# Supplementary material for: Design and Application of Mini‐libraries of miRNA Probes for an Efficient and Versatile miRNA‐mRNA Cross‐linking
Source: Chemistry. 2021 Jun 4;27(39):10193–200. doi: 10.1002/chem.202101171 (PMC8362200; doi:10.1002/chem.202101171)
Supplement: Supplementary file 1 — Supplementary [file CHEM-27-10193-s001.pdf]

Chemistry–A European Journal

Supporting Information

Design and Application of Mini-libraries of miRNA Probes for an Efficient and Versatile miRNA-mRNA Cross-linking

Anna L. Malinowska, Artur Laski, and Jonathan Hall*

Supporting Information

Table of Contents

I. Supplementary Figures S2

II. Experimental details S2

II.1. Synthesis and characterization of the trioxsalen analogues with ethylene glycol-based amino-linkers of different lengths S2

II.2. Post-synthetic functionalization of the ORNs with the CLICK chemistry S7

II.3. Biophysical characterization of the probes S7

II.3.1. Thermal stability studies S7

II.3.2. CD spectroscopy S7

II.4. Luciferase assay S7

III. NMR spectra and LC-MS chromatograms S8

III.1. NMR Spectra S8

III.2. LC-MS chromatograms of ORNs S17

III.2.1. miR-124 probes S18

III.2.2. miR-10b probes S22

III.2.3. miR-146a probes S24

III.2.4. miR-208a probes S26

III.2.5. miR-155-3p probes S27

IV. Chromatograms from the *in vitro* photo-cross-linking experiments S30

I. Supplementary Figures

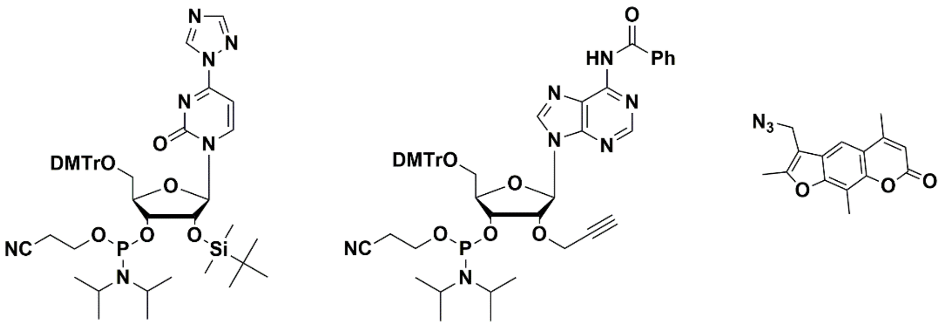

7 8 9

Figure S1: Structure of the fully protected O^4-triazolyluridine phosphoramidite (**7**), the fully protected 2'-*O*-propargyl adenosine phosphoramidite (**8**) and 4'-azidomethyltrioxsalen (**9**).

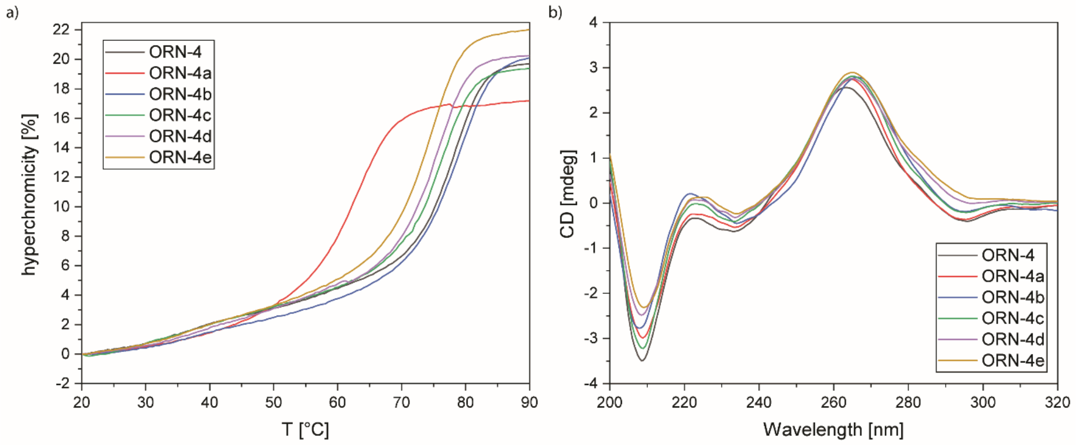

Figure S2: a) Melting profiles and b) CD spectra of the miR-124 analogues (ORN-**4** series) modified with the trioxsalen at the N^4-position of the cytidine C_6 through linkers of different lengths (number of ethylene glycol units: 0-4). Fully complementary counter-strand (ORN-**2**) was used as a second strand of the duplex. Conditions: 2 (a) or 3 (b) µM concentration of each strand of the duplex in 100 mM NaCl, 10 mM phosphate buffer and 0.1 mM Na_2EDTA, pH 7.0.

II. Experimental details

II.1. Synthesis and characterization of the trioxsalen analogues with ethylene glycol-based amino-linkers of different lengths

The synthesis was performed following (**1**, **2a**-**5a**, **2b**-**5b**, **6**-**9**) or adapting (**2c**-**5c** and **2d**-**5d**) the published protocols.

4'-Chloromethyl-4,5',8-trimethylpsoralen (1)[1]:

^{1}**H NMR** (400 MHz, Chloroform-*d*): δ = 7.60 (s, 1H), 6.27 (s, 1H), 4.74 (s, 2H), 2.58 (s, 3H), 2.55 – 2.50 (m, 6H). ^{13}**C NMR** (101 MHz, Chloroform-*d*) δ = 161.9, 155.4, 154.8, 153.3, 149.6, 116.6, 113.3, 112.3, 111.4, 109.7, 36.3, 19.5, 12.4, 8.6. **ESI-HR-MS** (positive mode): *m/z* calculated for $[M+H]^+$ ($C_{15}H_{14}ClO_3$): 277.0626, found: 277.0628.

4'-[(4-Hydroxy-2-oxa)butyl]-4,5',8-trimethylpsoralen (2a)[2]:

^{1}**H NMR** (400 MHz, Chloroform-*d*) δ = 7.54 (s, 1H), 6.15 (s, 1H), 4.65 (s, 2H), 3.79 – 3.71 (m, 2H), 3.63 – 3.55 (m, 2H), 2.52 – 2.45 (m, 6H), 2.44 (s, 3H). ^{13}**C NMR** (101 MHz, Chloroform-*d*) δ = 161.6, 155.0, 154.7, 153.4, 149.2,

125.1, 116.2, 112.7, 111.9, 111.6, 109.1, 71.2, 63.4, 62.0, 19.4, 12.4, 8.5. **ESI-HR-MS** (positive mode): *m/z* calculated for $[M+H]^+$ ($C_{17}H_{19}O_5$): 303.1227, found: 303.1227.

4’-[(4-Methanesulphonyloxy-2-oxa)butyl]-4,5’,8-trimethylpsoralen (3a)[2]:

^{1}H NMR (400 MHz, Chloroform-*d*) δ = 7.61 (s, 1H), 6.24 (d, *J* = 1.1 Hz, 1H), 4.70 (s, 2H), 4.41 – 4.36 (m, 2H), 3.79 – 3.74 (m, 2H), 2.98 (s, 3H), 2.56 (s, 3H), 2.52 – 2.49 (m, 6H). **^{13}C NMR** (101 MHz, Chloroform-*d*) δ = 161.6, 155.3, 154.8, 153.5, 149.4, 125.0, 116.5, 113.1, 111.7, 111.5, 109.4, 69.1, 67.7, 63.6, 37.8, 19.5, 12.5, 8.6. **ESI-HR-MS** (positive mode): *m/z* calculated for $[M+H]^+$ ($C_{18}H_{21}O_7S$): 381.1003, found: 381.1008.

4’-[(4-Azido-2-oxa)butyl]-4,5’,8-trimethylpsoralen (4a)[2]:

^{1}H NMR (400 MHz, Chloroform-*d*) δ = 7.65 (s, 1H), 6.26 – 6.22 (m, 1H), 4.69 (s, 2H), 3.69 – 3.64 (m, 2H), 3.43 – 3.38 (m, 2H), 2.57 (s, 3H), 2.50 (s, 6H). **^{13}C NMR** (101 MHz, Chloroform-*d*) δ = 161.7, 155.1, 154.9, 153.6, 149.5, 125.0, 116.5, 113.0, 111.9, 111.7, 109.3, 68.7, 63.7, 51.0, 19.5, 12.5, 8.6. **ESI-HR-MS** (positive mode): *m/z* calculated for $[M+H]^+$ ($C_{17}H_{18}N_3O_4$): 328.1292, found: 328.1293.

4’-[(4-Amino-2-oxa)butyl]-4,5’,8-trimethylpsoralen (5a)[2]:

^{1}H NMR (400 MHz, Chloroform-*d*) δ = 7.59 (s, 1H), 6.26 – 6.19 (m, 1H), 4.65 (s, 2H), 3.53 (t, *J* = 5.1 Hz, 2H), 2.90 (t, *J* = 5.0 Hz, 2H), 2.56 (s, 3H), 2.49 (d, *J* = 2.0 Hz, 6H). **^{13}C NMR** (101 MHz, Chloroform-*d*) δ = 161.7, 155.0, 154.9, 153.4, 149.4, 125.3, 116.4, 113.0, 112.1, 111.6, 109.3, 72.3, 63.4, 42.1, 19.5, 12.5, 8.6. **ESI-HR-MS** (positive mode): *m/z* calculated for $[M+H]^+$ ($C_{17}H_{20}NO_4$): 302.1387, found: 302.1388.

4’-[(7-Hydroxy-2,5-oxa)heptyl]-4,5’,8-trimethylpsoralen (2b)[2]:

^{1}H NMR (400 MHz, Chloroform-*d*) δ = 7.61 (s, 1H), 6.23 (d, *J* = 1.1 Hz, 1H), 4.69 (s, 2H), 3.74 – 3.66 (m, 4H), 3.65 – 3.57 (m, 4H), 2.56 (s, 3H), 2.49 (d, *J* = 1.3 Hz, 6H). **^{13}C NMR** (101 MHz, Chloroform-*d*) δ = 161.7, 155.2, 154.8, 153.5, 149.4, 125.2, 116.4, 113.0, 111.8, 111.7, 109.3, 72.6, 70.6, 69.1, 63.5, 61.9, 19.5, 12.5, 8.6. **ESI-HR-MS** (positive mode): *m/z* calculated for $[M+H]^+$ ($C_{19}H_{23}O_6$): 347.1489, found: 347.1482.

4’-[(7-Methanesulphonyloxy -2,5-oxa)heptyl]-4,5’,8-trimethylpsoralen (3b)[2]:

^{1}H NMR (400 MHz, Chloroform-*d*) δ = 7.59 (s, 1H), 6.23 (s, 1H), 4.68 (s, 2H), 4.38 – 4.33 (m, 2H), 3.78 – 3.74 (m, 2H), 3.69 – 3.67 (m, 2H), 3.64 – 3.61 (m, 2H), 3.00 (s, 3H), 2.56 (s, 3H), 2.49 (s, 6H). **^{13}C NMR** (101 MHz, Chloroform-*d*) δ = 161.6, 155.2, 154.8, 153.4, 149.4, 125.2, 116.4, 113.0, 111.8, 111.7, 109.3, 70.9, 69.2, 69.1, 69.0, 63.5, 37.8, 19.5, 12.5, 8.6. **ESI-HR-MS** (positive mode): *m/z* calculated for $[M+H]^+$ ($C_{20}H_{25}O_8S$): 425.1265, found: 425.1263.

4’-[(7-Azido-2,5-oxa)heptyl]-4,5’,8-trimethylpsoralen (4b)[2]:

^{1}H NMR (400 MHz, Chloroform-*d*) δ = 7.61 (s, 1H), 6.25 – 6.19 (m, 1H), 4.69 (s, 2H), 3.69 – 3.62 (m, 6H), 3.42 – 3.33 (m, 2H), 2.55 (s, 3H), 2.49 (s, 6H). **^{13}C NMR** (101 MHz, Chloroform-*d*) δ = 161.7, 155.2, 154.8, 153.5, 149.3, 125.2, 116.3, 112.9, 111.9, 111.8, 109.2, 70.9, 70.2, 69.2, 63.6, 50.8, 19.5, 12.5, 8.6. **ESI-HR-MS** (positive mode): *m/z* calculated for $[M+H]^+$ ($C_{19}H_{22}N_3O_5$): 372.1554, found: 372.1556.

4’-[(7-Amino-2,5-oxa)heptyl]-4,5’,8-trimethylpsoralen (5b)[2]:

^{1}H NMR (400 MHz, Chloroform-*d*) δ = 7.62 (s, 1H), 6.25 – 6.22 (m, 1H), 4.69 (s, 2H), 3.63 (s, 4H), 3.52 (t, *J* = 5.2 Hz, 2H), 2.88 (t, *J* = 5.2 Hz, 2H), 2.56 (s, 3H), 2.51 – 2.48 (m, 6H). **^{13}C NMR** (101 MHz, Chloroform-*d*) δ = 161.7, 155.2, 154.8, 153.5, 149.4, 125.2, 116.3, 113.0, 111.9, 111.8, 109.3, 73.0, 70.5, 69.0, 63.5, 41.7, 19.6, 12.5, 8.6. **ESI-HR-MS** (positive mode): *m/z* calculated for $[M+Na]^+$ ($C_{19}H_{23}NNaO_5$): 368.1468, found: 368.1466.

4’-[(10-Hydroxy-2,5,8-oxa)decyl]-4,5’,8-trimethylpsoralen (2c):

The compound was prepared adjusting the published procedure for the synthesis of the analogues with shorter ethylene glycol-based linkers[2]. 4'-Chloromethyl-4,5',8-trimethylpsoralen (**1**) (1.00 g, 3.61 mmol, 1.00 eq) and triethylene glycol (38.0 mL, 0.29 mol, 80.00 eq) were heated in acetone (22 mL) to 50-60°C and stirred at this temperature for around 48 h. In the meantime, the white suspension turned into a clear brown solution. The

acetone and triethylene glycol were then partially removed on the rotary evaporator. The residue was diluted with DCM. The organic phase was washed several times with water, brine, dried over Na_2SO_4 and concentrated under reduced pressure. The crude product was diluted with a small amount of DCM and purified twice by flash column chromatography (gradient: DCM/MeOH 100/0 to 93/7) to give the slightly impure product (an excess of the triethylene glycol present) in the form of a dark-brown oil (1.08 g, 77%). **^{1}H NMR** (400 MHz, Chloroform-*d*) δ = 7.64 (s, 1H), 6.23 (d, *J* = 1.1 Hz, 1H), 4.68 (s, 2H), 3.72 – 3.66 (m, 6H), 3.64 – 3.58 (m, 6H), 2.56 (s, 3H), 2.50 (d, *J* = 1.1 Hz, 3H), 2.49 (s, 3H). **^{13}C NMR** (101 MHz, Chloroform-*d*) δ = 161.7, 155.2, 154.8, 153.6, 149.4, 125.2, 116.3, 112.9, 2 x 111.9, 109.2, 72.6, 70.8, 70.7, 70.5, 69.0, 63.5, 61.9, 19.5, 12.5, 8.6. **ESI-HR-MS** (positive mode): *m/z* calculated for $[M+Na]^+$ ($C_{21}H_{26}NaO_7$): 413.1571, found: 413.1566.

4’-[(10-Methanesulphonyloxy-2,5,8-oxa)decyl]-4,5’,8-trimethylpsoralen (3c):

The compound was prepared adjusting the published procedure for the synthesis of the analogues with shorter ethylene glycol-based linkers[2]. 4'-[(10-Hydroxy-2,5,8-oxa)decyl]-4,5',8-trimethylpsoralen (**2c**) (0.70 g, 1.79 mmol, 1.00 eq) was dissolved in DCM (5.6 mL) under argon atmosphere. Triethylamine (0.40 mL, 2.87 mmol, 1.60 eq) was then added and the reaction mixture was flushed with argon and cooled down to 0°C. Subsequently, methanesulphonyl chloride (0.19 mL, 2.51 mmol, 1.40 eq) was added dropwise. When the addition was completed, the reaction mixture was stirred for 15 minutes at around 0°C and then was left to warm up to room temperature overnight. The reaction mixture was diluted with DCM, washed with water (three times), brine, dried over anhydrous Na_2SO_4 and concentrated under reduced pressure. The crude product was diluted with a small amount of DCM and purified by flash column chromatography (gradient: DCM/MeOH 100/0 to 90/10) to give the mesylated product as an amber oil (0.79 g; 94%). **^{1}H NMR** (400 MHz, Chloroform-*d*) δ = 7.61 (s, 1H), 6.24 (d, *J* = 1.2 Hz, 1H), 4.68 (s, 2H), 4.36 – 4.33 (m, 2H), 3.75 – 3.73 (m, 2H), 3.66 – 3.64 (m, 6H), 3.62 – 3.61 (m, 2H), 3.03 (s, 3H), 2.57 (s, 3H), 2.50 – 2.49 (m, 6H). **^{13}C NMR** (101 MHz, Chloroform-*d*) δ = 161.7, 155.2, 154.8, 153.5, 149.4, 125.3, 116.3, 113.0, 111.9, 111.8, 109.3, 70.9, 70.8, 70.7, 69.2, 69.2, 69.0, 63.5, 37.8, 19.5, 12.5, 8.6. **ESI-HR-MS** (positive mode): *m/z* calculated for $[M+H]^+$ ($C_{22}H_{29}O_9S$): 469.1527, found: 469.1530.

4’-[(10-Azido-2,5,8-oxa)decyl]-4,5’,8-trimethylpsoralen (4c):

The compound was prepared adjusting the published procedure for the synthesis of the analogues with shorter ethylene glycol-based linkers[2]. 4'-[(10-Methanesulphonyloxy-2,5,8-oxa)decyl]-4,5',8-trimethylpsoralen (**3c**) (1.10 g, 2.35 mmol, 1.00 eq) and sodium azide (0.31 g, 4.70 mmol, 2.00 eq) were refluxed in ethanol (12 mL) for 20 h. Then, another portion of sodium azide (0.15 g, 2.31 mmol, 0.98 eq) was added and the reaction mixture was refluxed for 24 h. The reaction mixture was then cooled down to room temperature and washed with cold water (three times), brine, dried over Na_2SO_4 and concentrated on the rotary evaporator. The crude product was diluted with a small amount of DCM and purified by flash column chromatography (gradient: DCM/MeOH 100/0 to 93/7) to give an amber oil as the slightly impure product (0.84 g, 86%). **^{1}H NMR** (400 MHz, Chloroform-*d*) δ = 7.63 (s, 1H), 6.24 (d, *J* = 1.1 Hz, 1H), 4.69 (s, 2H), 3.69 – 3.67 (m, 4H), 3.65 – 3.62 (m, 6H), 3.37 – 3.34 (m, 2H), 2.57 (s, 3H), 2.50 (d, *J* = 1.2 Hz, 3H), 2.49 (s, 3H). **^{13}C NMR** (101 MHz, Chloroform-*d*) δ = 161.7, 155.1, 154.9, 153.5, 149.4, 125.3, 116.3, 112.9, 112.0, 111.9, 109.3, 71.0, 70.9, 70.8, 70.2, 69.1, 63.5, 50.8, 19.5, 12.5, 8.6. **ESI-HR-MS** (positive mode): *m/z* calculated for $[M+Na]^+$ ($C_{21}H_{25}N_3NaO_6$): 438.1636, found: 438.1629.

4’-[(10-Amino-2,5,8-oxa)decyl]-4,5’,8-trimethylpsoralen (5c):

The compound was prepared adjusting the published procedure for the synthesis of the analogues with shorter ethylene glycol-based linkers[2]. 4'-[(10-Azido-2,5,8-oxa)decyl]-4,5',8-trimethylpsoralen (**4c**) (0.70 g, 1.68 mmol, 1.00 eq) was dissolved in dry THF (4.0 mL). Triphenylphosphine (0.53 g, 2.02 mmol, 1.20 eq) and two drops of water were added to the stirred solution. After stirring at room temperature overnight, the reaction mixture was concentrated under reduced pressure. The residue was dissolved in DCM and extracted with 1% aqueous HCl solution (four times). Combined acidic layers were carefully treated with 5 M aqueous NaOH solution until the basic pH (pH~10) was reached. The water layer was extracted with DCM (four times). Combined organic layers were washed with water, brine, dried over Na_2SO_4, and concentrated on the rotary evaporator to give the free amine. The crude product was diluted with DCM and purified by flash column chromatography (gradient:

DCM/MeOH 100/0 to 70/30). After drying on the high vacuum pump, the desired final product was obtained in the form of a dark-yellow oil (0.51 g, 78%). **^{1}H NMR** (400 MHz, Chloroform-*d*) δ = 7.62 (s, 1H), 6.23 – 6.22 (m, 1H), 4.70 (s, 2H), 3.67 – 3.60 (m, 8H), 3.51 (t, *J* = 5.2 Hz, 2H), 2.87 (t, *J* = 5.2 Hz, 2H), 2.55 (s, 3H), 2.49 (d, *J* = 1.1 Hz, 3H), 2.48 (s, 3H). **^{13}C NMR** (101 MHz, Chloroform-*d*) δ = 161.7, 155.2, 154.8, 153.5, 149.3, 125.2, 116.3, 112.9, 111.9, 111.9, 109.2, 72.7, 70.9, 70.7, 70.4, 69.0, 63.4, 41.6, 19.6, 12.5, 8.6. **ESI-HR-MS** (positive mode): *m/z* calculated for $[M+Na]^+$ ($C_{21}H_{27}NNaO_6$): 412.1731, found: 412.1734.

4'-[(13-Hydroxy-2,5,8,11-oxa)tridecyl]-4,5',8-trimethylpsoralen (2d):

The compound was prepared adjusting the published procedure for the synthesis of the analogues with shorter ethylene glycol-based linkers[2]. 4'-Chloromethyl-4,5',8-trimethylpsoralen (**1**) (1.00 g, 3.61 mmol, 1.00 eq) and tetraethylene glycol (56 mL, 0.33 mol, 90.00 eq) were heated in acetone (30 mL) to 50-60°C and stirred at this temperature overnight. In the meantime, the white suspension turned into a clear, light brown solution. The acetone and tetraethylene glycol were partially removed on the rotary evaporator. The residue was diluted with chloroform. The organic phase was washed several times with water, brine, dried over Na_2SO_4 and concentrated under reduced pressure. The crude product was dried on the high-vacuum pump. The ^{1}H NMR spectrum showed that the starting material was still present. The crude and tetraethylene glycol (25 mL, 0.15 mol, 40.18 eq) were heated in acetone (15 mL) to 50-60°C and stirred at this temperature overnight. The reaction mixture was concentrated on the rotary evaporator and the residue was diluted with DCM. The organic phase was washed with water (four times), brine (two times), dried over Na_2SO_4 and concentrated. The crude product was dried overnight on the high-vacuum pump. Afterwards, it was diluted with DCM and purified twice by flash column chromatography (gradient: DCM/MeOH 100/0 to 90/10) to give the desired product with a small excess of the tetraethylene glycol (dark-brown oil; 1.34 g, 87%). **^{1}H NMR** (400 MHz, Chloroform-*d*) δ = 7.63 (s, 1H), 6.23 (d, *J* = 1.1 Hz, 1H), 4.69 (s, 2H), 3.71 – 3.69 (m, 2H), 3.66 – 3.61 (m, 12H), 3.60 – 3.57 (m, 2H), 2.56 (s, 3H), 2.50 (d, *J* = 1.2 Hz, 3H), 2.49 (s, 3H). **^{13}C NMR** (101 MHz, Chloroform-*d*) δ = 161.7, 155.2, 154.8, 153.6, 149.4, 125.3, 116.3, 112.9, 112.0, 111.9, 109.2, 72.6, 70.9, 70.8, 2 x 70.7, 70.4, 69.1, 63.5, 61.9, 19.6, 12.5, 8.6. **ESI-HR-MS** (positive mode): *m/z* calculated for $[M+Na]^+$ ($C_{23}H_{30}NaO_8$): 457.1833, found: 457.1826.

4'-[(13-Methanesulphonyloxy-2,5,8,11-oxa)tridecyl]-4,5',8-trimethylpsoralen (3d):

The compound was prepared adjusting the published procedure for the synthesis of the analogues with shorter ethylene glycol-based linkers[2]. 4'-[(13-Hydroxy-2,5,8,11-oxa)tridecyl]-4,5',8-trimethylpsoralen (**2d**) (1.30 g, 2.99 mmol, 1.00 eq) was dissolved in DCM (10 mL) under argon atmosphere. Triethylamine (0.67 mL, 4.79 mmol, 1.60 eq) was then added and the reaction mixture was flushed with argon and cooled down to 0°C. Then, methanesulphonyl chloride (0.32 mL, 4.19 mmol, 1.40 eq) was added dropwise. When the addition was completed, the reaction mixture was stirred for 15 minutes at around 0°C and then was left to warm up to room temperature overnight. The reaction mixture was diluted with DCM, washed with water (three times), brine (two times), dried over Na_2SO_4 and concentrated on the rotary evaporator to give the mesylated product as a dark-brown oil (1.50 g, quant.). The crude product was used in the next step without further purification. **^{1}H NMR** (400 MHz, Chloroform-*d*) δ = 7.62 (s, 1H), 6.23 (d, *J* = 1.3 Hz, 1H), 4.69 (s, 2H), 4.36 – 4.34 (m, 2H), 3.74 – 3.72 (m, 2H), 3.64 – 3.61 (m, 12H), 3.06 (s, 3H), 2.56 (s, 3H), 2.49 (d, *J* = 1.2 Hz, 3H), 2.49 (s, 3H). **^{13}C NMR** (101 MHz, Chloroform-*d*) δ = 161.7, 155.2, 154.8, 153.5, 149.4, 125.3, 116.3, 112.9, 112.0, 111.9, 109.2, 70.9, 3 x 70.7, 70.6, 69.4, 69.1, 69.0, 63.4, 37.9, 19.5, 12.5, 8.6. **ESI-HR-MS** (positive mode): *m/z* calculated for $[M+H]^+$ ($C_{24}H_{33}O_{10}S$): 513.1789, found: 513.1783.

4'-[(13-Azido-2,5,8,11-oxa)tridecyl]-4,5',8-trimethylpsoralen (4d):

The compound was prepared adjusting the published procedure for the synthesis of the analogues with shorter ethylene glycol-based linkers[2]. 4'-[(13-Methanesulphonyloxy-2,5,8,11-oxa)tridecyl-4,5',8-trimethylpsoralen (**3d**) (1.40 g, 2.73 mmol, 1.00 eq) and sodium azide (0.36 g, 5.46 mmol, 2.00 eq) were refluxed in ethanol (14 mL) for 20 h. Then, another portion of sodium azide (0.18 g, 2.73 mmol, 1.00 eq) was added and the reaction mixture was refluxed for 18 h. The reaction mixture was cooled down to room temperature and washed with cold water (three times), brine, dried over Na_2SO_4 and concentrated on the rotary evaporator. The crude product was

diluted with a small amount of DCM and purified by flash column chromatography (gradient: DCM/MeOH 100/0 to 93/7) to give a dark-brown oil as the desired product (1.09 g, 87%). **^{1}H NMR** (400 MHz, Chloroform-*d*) δ = 7.63 (s, 1H), 6.24 (d, *J* = 1.1 Hz, 1H), 4.69 (s, 2H), 3.67 – 3.62 (m, 14H), 3.39 – 3.33 (m, 2H), 2.57 (s, 3H), 2.50 (d, *J* = 1.1 Hz, 3H), 2.49 (s, 3H). **^{13}C NMR** (101 MHz, Chloroform-*d*) δ = 161.7, 155.1, 154.9, 153.5, 149.4, 125.3, 116.3, 112.9, 112.0, 111.9, 109.2, 70.9, 4 x 70.8, 70.2, 69.1, 63.5, 50.8, 19.6, 12.5, 8.6. **ESI-HR-MS** (positive mode): *m/z* calculated for $[M+Na]^+$ ($C_{23}H_{29}N_3NaO_7$): 482.1898, found: 482.1891.

4’-[(13-Amino-2,5,8,11-oxa)tridecyl]-4,5’,8-trimethylpsoralen (5d):

The compound was prepared adjusting the published procedure for the synthesis of the analogues with shorter ethylene glycol-based linkers[2]. 4'-(13-Azido-2,5,8,11-oxa)tridecyl]-4,5',8-trimethylpsoralen (**4d**) (0.90 g, 1.96 mmol, 1.00 eq) was dissolved in dry THF (4.0 mL). Triphenylphosphine (0.62 g; 2.35 mmol, 1.20 eq) and two drops of water were added to the stirred solution. After stirring at room temperature overnight, the reaction mixture was concentrated on the rotary evaporator to give a yellow residue. The residue was dissolved in DCM and extracted with 1% aqueous HCl solution (four times). Combined acidic layers were carefully treated with 5 M aqueous NaOH solution until basic pH (pH~10) was reached. The water layer was extracted with DCM (five times). Combined organic layers were washed with water, brine, dried over Na_2SO_4 and concentrated under reduced pressure to give the free amine as the final product (brown oil; 0.53 g; 63%). **^{1}H NMR** (400 MHz, Chloroform-*d*) δ = 7.63 (s, 1H), 6.24 (s, 1H), 4.69 (s, 2H), 3.68 – 3.60 (m, 12H), 3.49 (t, *J* = 5.2 Hz, 2H), 2.81 (t, *J* = 5.1 Hz, 2H), 2.57 (s, 3H), 2.50 (s, 3H), 2.49 (s, 3H). **^{13}C NMR** (101 MHz, Chloroform-*d*) δ = 161.7, 155.2, 154.9, 153.6, 149.4, 125.2, 116.4, 113.0, 112.0, 111.9, 109.3, 73.2, 70.9, 3 x 70.7, 70.4, 69.1, 63.5, 41.8, 19.6, 12.5, 8.6. **ESI-HR-MS** (positive mode): *m/z* calculated for $[M+Na]^+$ ($C_{23}H_{31}NNaO_7$): 456.1993, found: 456.1983.

4'-Aminomethyl-4,5',8-trimethylpsoralen (6)[3]:

^{1}H NMR (400 MHz, Methanol-d_4) δ = 7.86 (s, 1H), 6.26 (d, *J* = 1.2 Hz, 1H), 4.06 (s, 2H), 2.56 (s, 3H), 2.52 (s, 3H), 2.50 (s, 3H). **^{13}C NMR** (101 MHz, Methanol-d_4) δ = 167.5, 163.5, 156.5, 155.9, 150.3, 126.1, 117.4, 113.3, 2 x 113.0, 109.8, 34.7, 19.4, 12.0, 8.3. **ESI-HR-MS** (positive mode): *m/z* calculated for $[M+H]^+$ ($C_{15}H_{16}NO_3$): 258.1125, found: 258.1128.

Fully protected *O*^{4}-triazolyluridine phosphoramidite (7)[4]:

^{1}H NMR (400 MHz, Chloroform-*d*) δ = 9.23 (d, *J* = 4.1 Hz, 1H), 8.86 (dd, *J* = 17.9, 7.2 Hz, 1H), 8.07 (d, *J* = 6.3 Hz, 1H), 7.44 (ddd, *J* = 20.2, 8.2, 1.4 Hz, 2H), 7.37 – 7.27 (m, 7H), 6.86 (dt, *J* = 8.5, 3.8 Hz, 4H), 6.45 (dd, *J* = 64.3, 7.2 Hz, 1H), 5.89 – 5.79 (m, 1H), 4.43 (ddd, *J* = 28.7, 8.7, 4.4 Hz, 3H), 3.82 – 3.80 (m, 6H), 3.78 – 3.41 (m, 6H), 2.60 – 2.54 (m, 1H), 2.42 (t, *J* = 6.3 Hz, 1H), 1.17 – 1.08 (m, 9H), 0.98 (d, *J* = 6.8 Hz, 3H), 0.93 (d, *J* = 4.3 Hz, 9H), 0.31 (d, *J* = 4.4 Hz, 3H), 0.18 (s, 3H). **^{31}P NMR** (162 MHz, Chloroform-*d*) δ = 151.2, 148.7.

2'-*O*-propargyl-*N*^{4}-benzoyladenosine phosphoramidite (8)[5]:

^{1}H NMR (400 MHz, Chloroform-*d*) δ = 8.87 (s, 1H), 8.68 (d, *J* = 8.9 Hz, 1H), 8.20 (d, *J* = 15 Hz, 1H), 7.96 (d, *J* = 7.3 Hz, 2H), 7.55 (t, *J* = 7.4 Hz, 1H), 7.48 (t, *J* = 7.5 Hz, 2H), 7.36 (t, *J* = 6.9 Hz, 2H,), 7.28 – 7.23 (m, 4H), 7.19 (s, 10H), 6.76 – 6.71 (m, 4H), 6.15 (t, *J* = 6.1 Hz, 1H), 5.02 (t, *J* = 5.5 Hz, 1H), 4.59 – 4.56 (m, 1H), 4.35 (d, *J* = 3.3 Hz, 1H), 4.30 – 4.21 (m, 2H), 4.21 (d, *J* = 2.3 Hz, 1H), 3.71 (d, *J* = 2.2 Hz, 6H), 3.58 – 3.51 (m, 4H), 3.41 (q, *J* = 7.0 Hz, 1H), 3.29 – 3.24 (m, 1H), 2.6 (dt, *J* = 4.8 Hz, *J* = 1.5 Hz, 1H), 2.32 (t, *J* = 6.4 Hz, 1H), 2.25 (t, *J* = 2.4 Hz, 1H), 2.22 (t, *J* = 2.4 Hz, 1H), 1.51 (s, 4H), 1.47 (s, 1H), 1.22 – 1.19 (m, 3H), 1.16 – 1.12 (m, 10H), 1.00 (d, *J* = 6.8 Hz, 2H). **^{31}P NMR** (162 MHz, Chloroform-*d*) δ = 150.96, 150.40.

4'-azidomethyltrioxsalen (9)[1]:

^{1}H NMR (400 MHz, Chloroform-*d*) δ = 7.52 (s, 1H), 6.23 (s, 1H), 4.44 (s, 2H), 2.55 (s, 3H), 2.52 (s, 3H), 2.49 (s, 3H). **^{13}C NMR** (101 MHz, Chloroform-*d*) δ = 161.2, 155.5, 154.5, 153.0, 149.4, 124.3, 116.3, 113.0, 111.0, 109.7, 109.4, 44.5, 19.3, 12.2, 8.43. **ESI-HR-MS** (positive mode): *m/z* calculated for $[M+H]^+$ ($C_{15}H_{14}N_3O_3$): 284.1026, found: 284.1030.

II.2. Post-synthetic functionalization of the ORNs with the CLICK chemistry

A CuAAC reaction between the alkyne-modified oligoribonucleotides (ORN-**1** and ORN-**3**; alkyne functionality was introduced into the oligonucleotide sequence using a fully protected 2'-*O*-propargyl adenosine phosphoramidite (**8**)) and 4'-azidomethyltrioxsalen (**9**) was performed as described previously with minor modifications[1, 6]. After the solid-phase synthesis, the CPG containing the alkynyl-modified RNA was suspended in 300 µL of water/PBS (pH 7.4) (1:1) mixture. Subsequently, 4'-azidomethyltrioxsalen (**9**) (20 eq, 1 µmol in 60 µL of DMF), TBTA (10 eq, 500 nmol in 20 µL of DMF), Na-ascorbate (10 eq, 500 nmol, 10 µL of a solution containing 10 mg of Na-ascorbate in 1 mL of water) and $CuSO_4$ x $5H_2O$ (1 eq, 50 nmol, 10 µL of a solution containing 12.5 mg of $CuSO_4$ x $5H_2O$ in 10 mL of water) were added to the suspension in this order. All solutions were freshly prepared before use. The reaction mixture was shaken (1400 rpm) overnight at 45°C in a ThermoMixer C (Eppendorf). The CPG was filtered off and washed three times with 0.5 mL of each: DMF, 0.1 N aqueous EDTA, DMF, ACN, chloroform and dried under vacuum. Afterwards, the post-synthetically modified oligoribonucleotides were deprotected and purified according to the standard procedure described in the Experimental section of the Manuscript.

II.3. Biophysical characterization of the probes

II.3.1. Thermal stability studies

UV melting profiles were recorded on a Cary 300 instrument (Varian) equipped with a thermocontroller. Oligonucleotides were dissolved in 200 µL of the buffer containing 100 mM NaCl, 10 mM Na-phosphate buffer and 0.1 mM Na_2EDTA (pH 7.0) so that the final concentration of each strand of the duplex was 2 µM. Samples were transferred to quartz cuvettes and 100 µL of mineral oil was added on the top of the samples to prevent the evaporation of the solutions during measurements. Samples were annealed by heating from 20°C to 90°C (5°C/min) and cooling down to 20°C. Melting curves were obtained by the measurement of the A_{260} absorption during the heating step (heating to 90°C, 0.5°C/min), which was repeated three times. Melting temperature was calculated as the average value from the maxima of the first derivatives of 3 consecutive melting curves. Representative melting curves are shown in Figure S2a.

II.3.2. CD spectroscopy

Oligonucleotides were dissolved in 250 µL of the buffer containing 100 mM NaCl, 10 mM Na-phosphate buffer and 0.1 mM Na_2EDTA (pH 7.0) so that the final concentration of each strand of the duplex was 3 µM. Oligonucleotides were annealed by heating to 95°C and then cooled down to room temperature over 2 h. CD spectra were recorded between 200 and 320 nm at 20°C on a Jasco J-10 spectropolarimeter (50 nm/min, 0.5 nm data pitch, 5 nm bandwidth, light path 1 nm). 4 scans were averaged. The data were smoothed using a Savitzky–Golay filter (convolution width 25).

II.4. Luciferase assay

HEK293T cells (ATCC® CRL-3216™, Wesel, DE) were seeded in Dulbecco's Modified Eagle Medium (Gibco, Invitrogen, Basel, CH) supplemented with 10% Fetal Bovine Serum (Gibco, Invitrogen, Basel) in a 96-well plate. After 8 h, cells were transfected with RNA samples, in technical triplicates, using Lipofectamine 2000 (cat. no. 11668019, ThermoFisher Scientific, Basel, CH), according to the manufacturer protocol. RNA transfection was performed with increasing concentrations (0, 2.5, 10, 40 nM) of the native miR-124 (ORN-**4**), siRenilla acting against *Renilla* luciferase (siRenilla), trioxsalen-modified miR-124 probes (ORN-**4b** to ORN-**4e** and ORN-**5a** to ORN-**5d**) or the Negative Control RNA (siRND2; double-stranded RNAs carrying randomized base pairs[7]). After 24 h from the RNA transfection, cells were transfected with 20 ng/well of the reporter plasmid, using JetPEI (10110N, Polyplus, Transfection, Illkirch, FR) according to the manufacturer's protocol. After 48 h from the plasmid transfection, luciferase readout was conducted according to the manufacturer's protocol (Dual-Glo Luciferase Assay System, Promega) with 30 µL/well Dual-Glo® Luciferase Reagent diluted in 1:1 ratio with water, and 15 µL/well of Dual-Glo® Stop & Glo® Reagent. Luminescence readout was performed on a microtiter plate

reader (Mithras LB940, Berthold Technologies). Readout values were normalized to the firefly luciferase counts and additionally to the corresponding values obtained from 0 nM treatment.

III. NMR spectra and LC-MS chromatograms

III.1. NMR Spectra

^{1}H and ^{13}C NMR (Chloroform-*d*) spectra of the compound **2c**

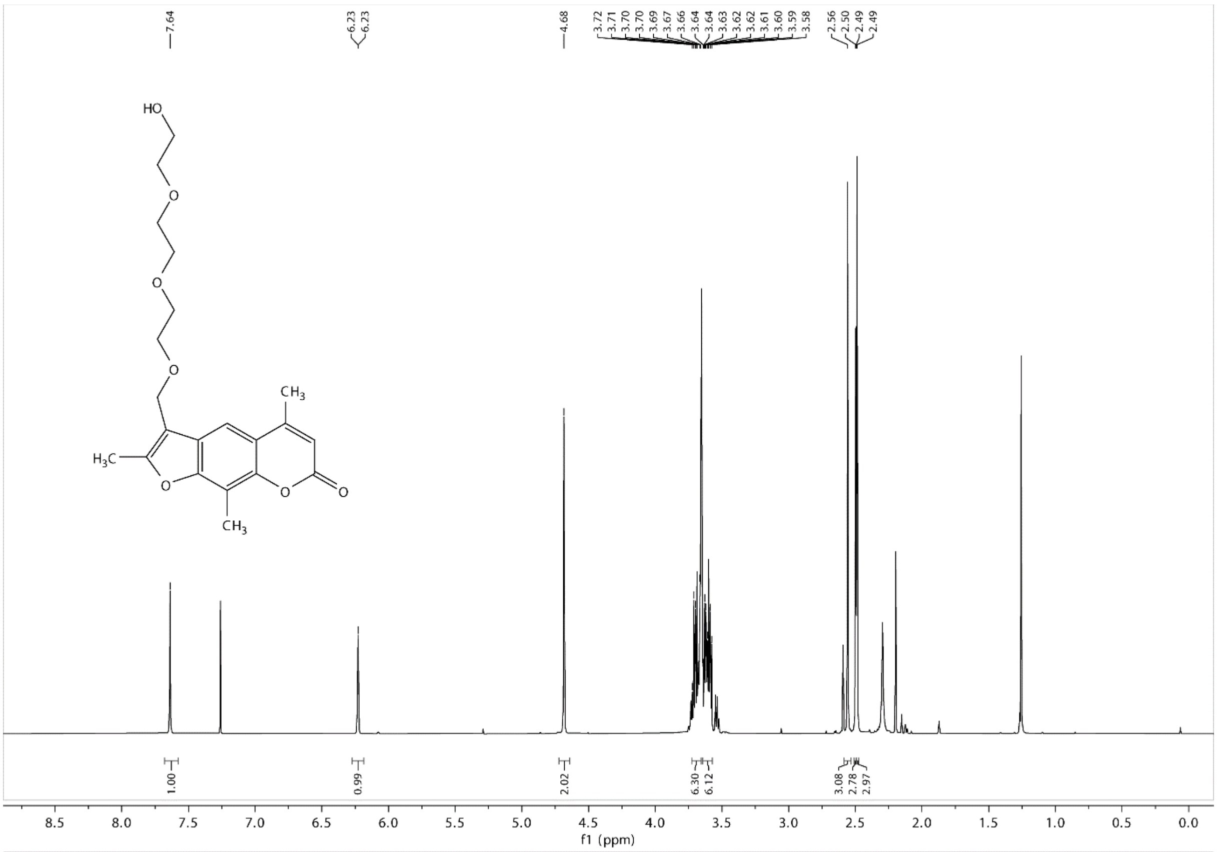

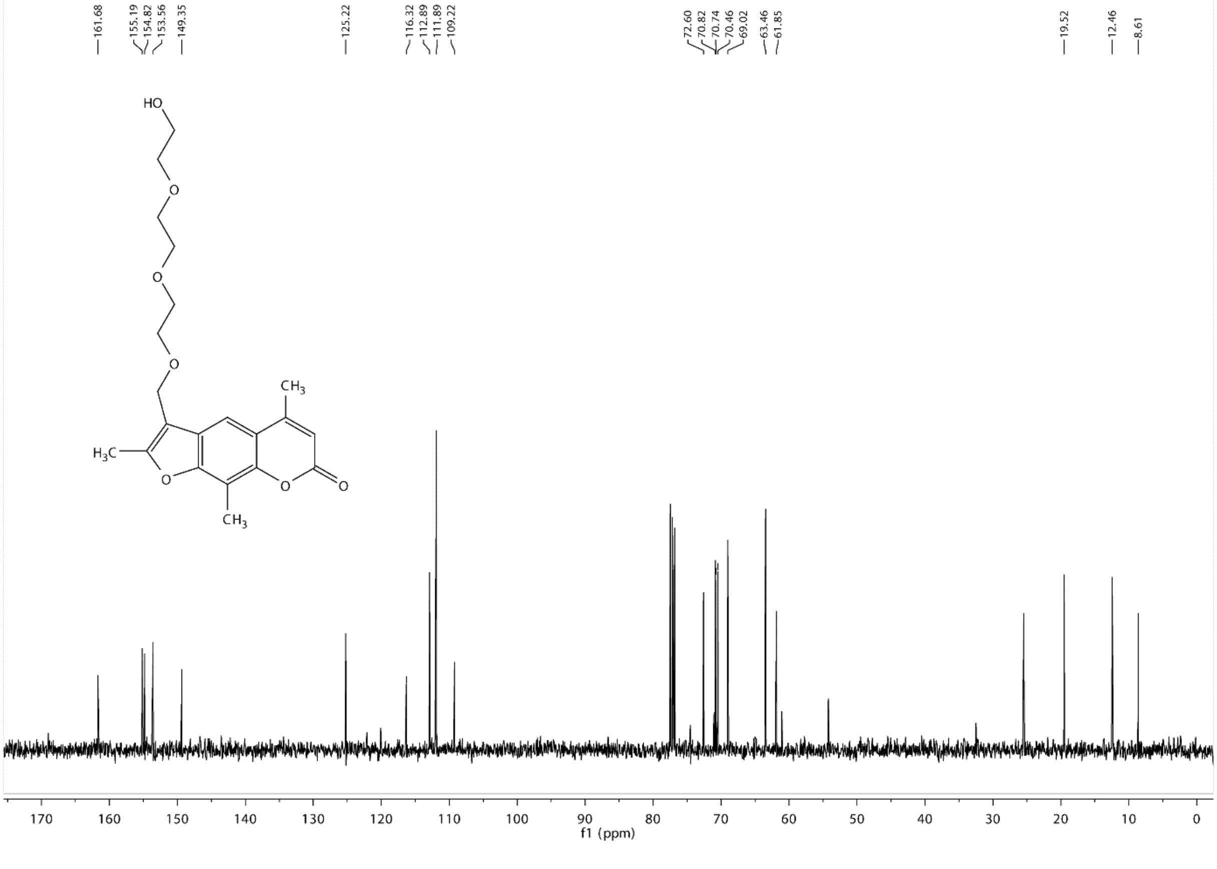

^{1}H and ^{13}C NMR (Chloroform-*d*) spectra of the compound **3c**

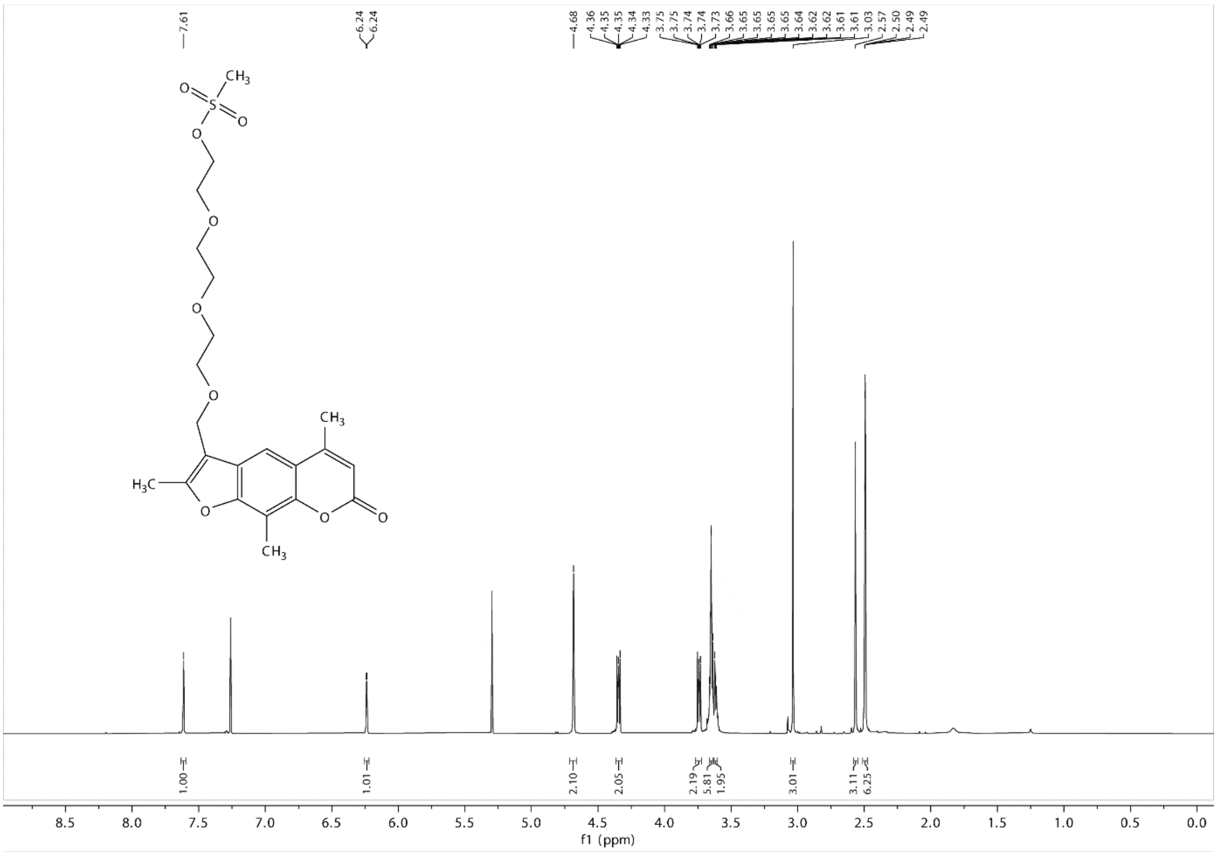

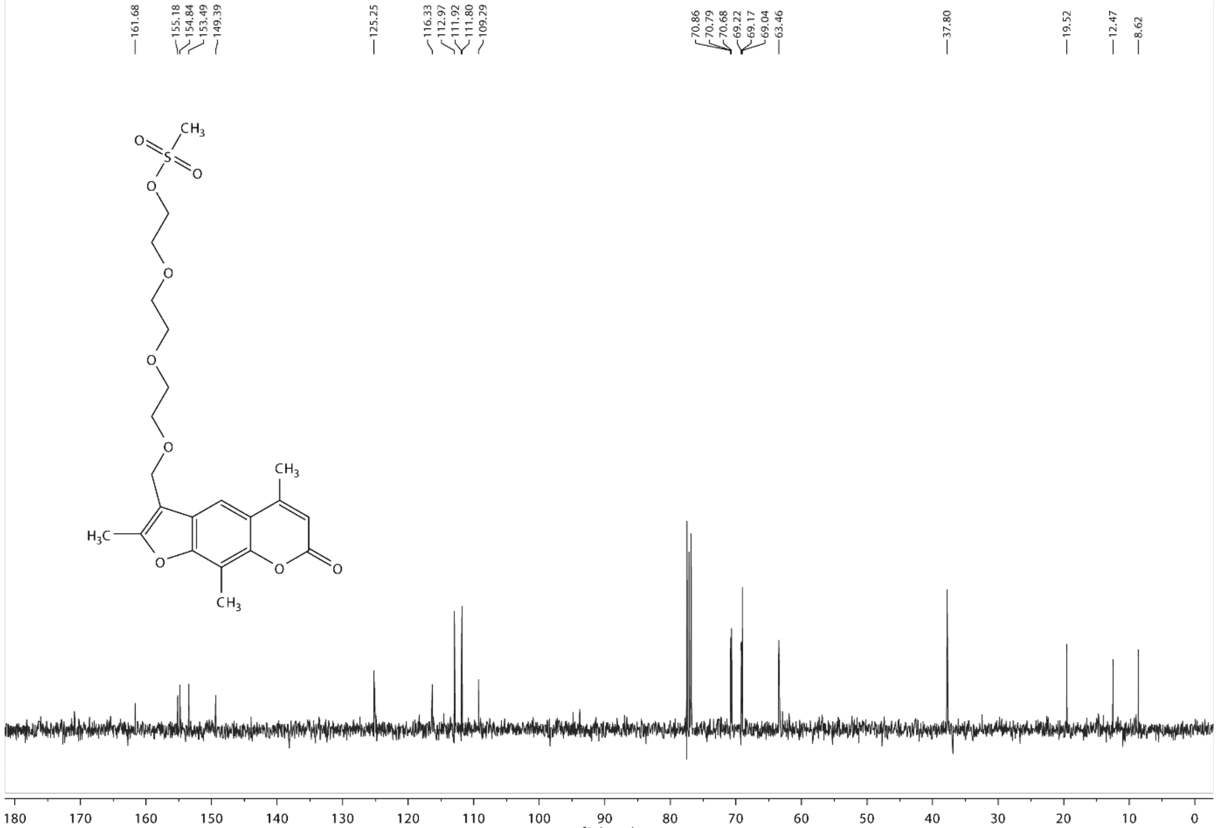

^{1}H and ^{13}C NMR (Chloroform-*d*) spectra of the compound **4c**

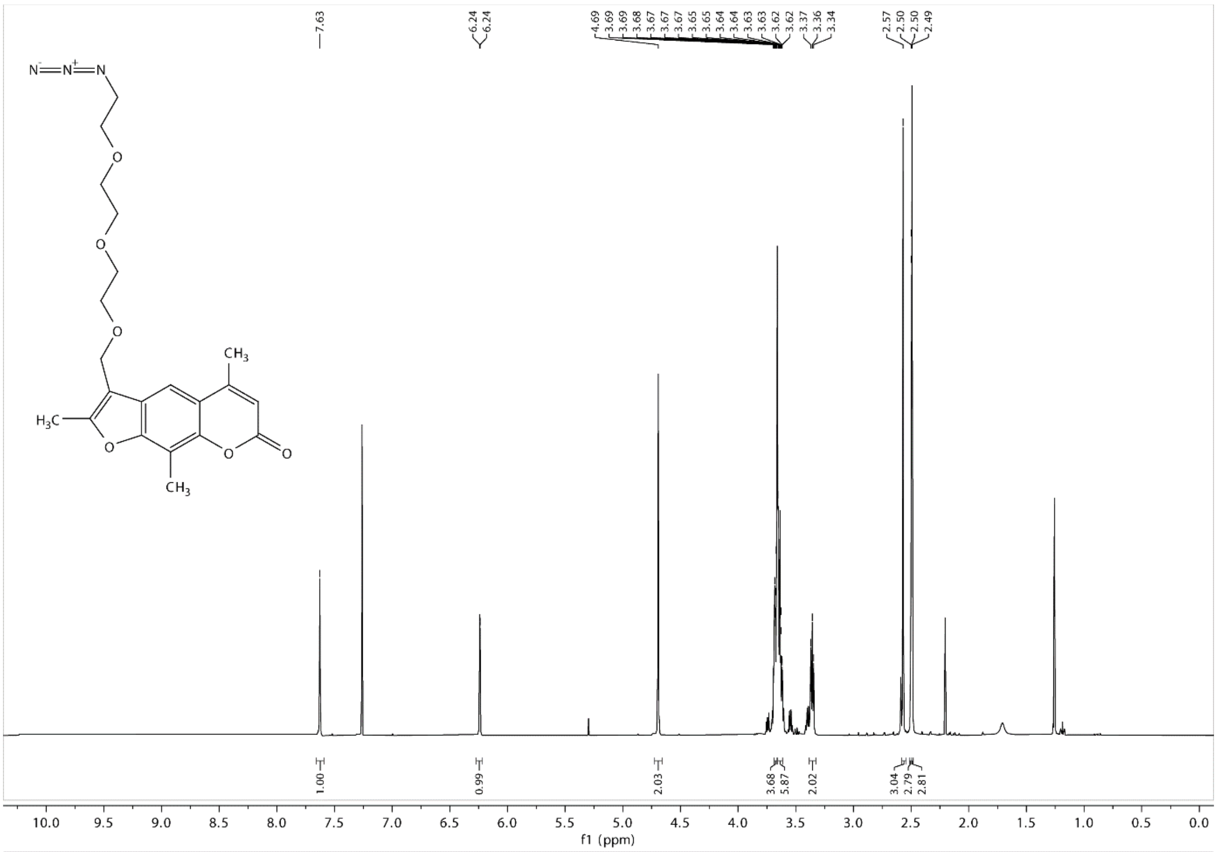

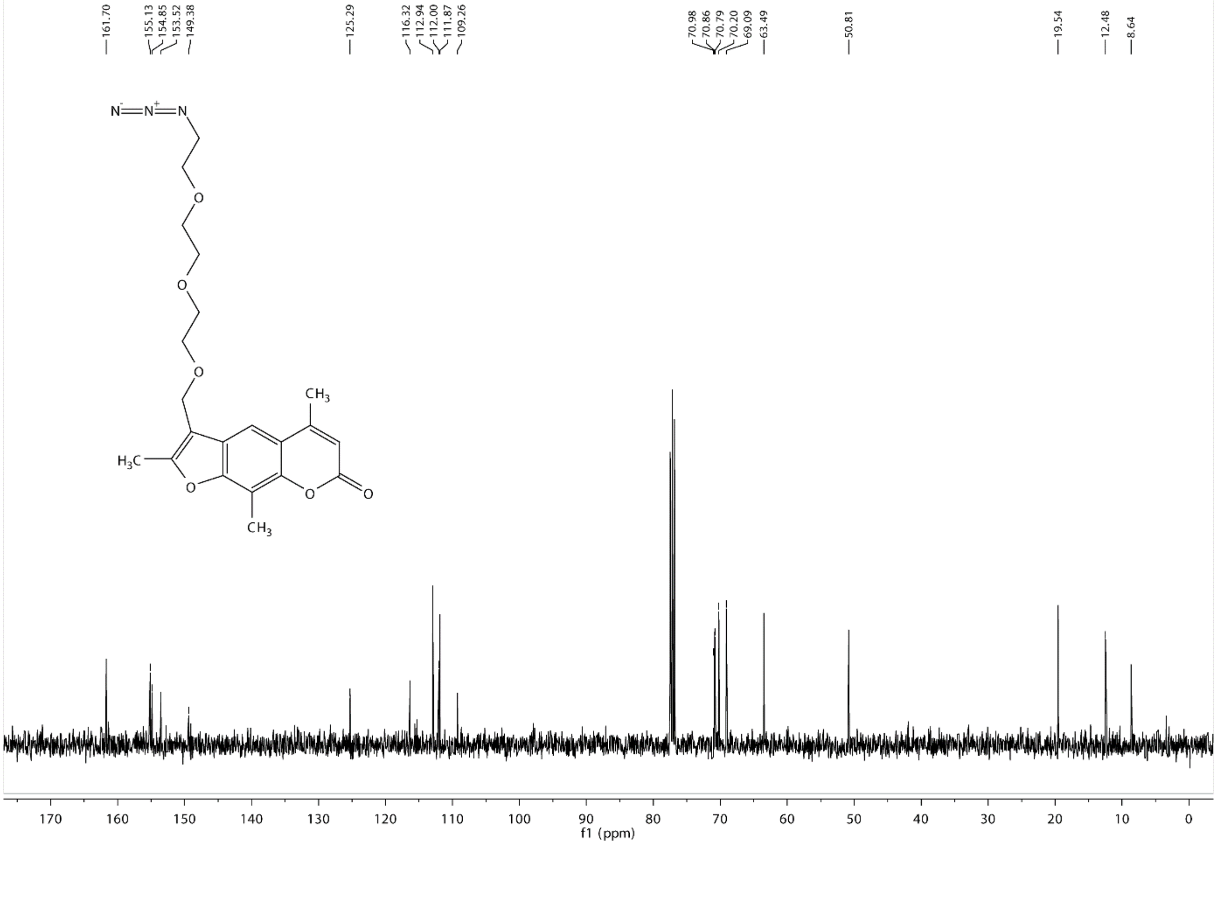

^{1}H and ^{13}C NMR (Chloroform-*d*) spectra of the compound **5c**

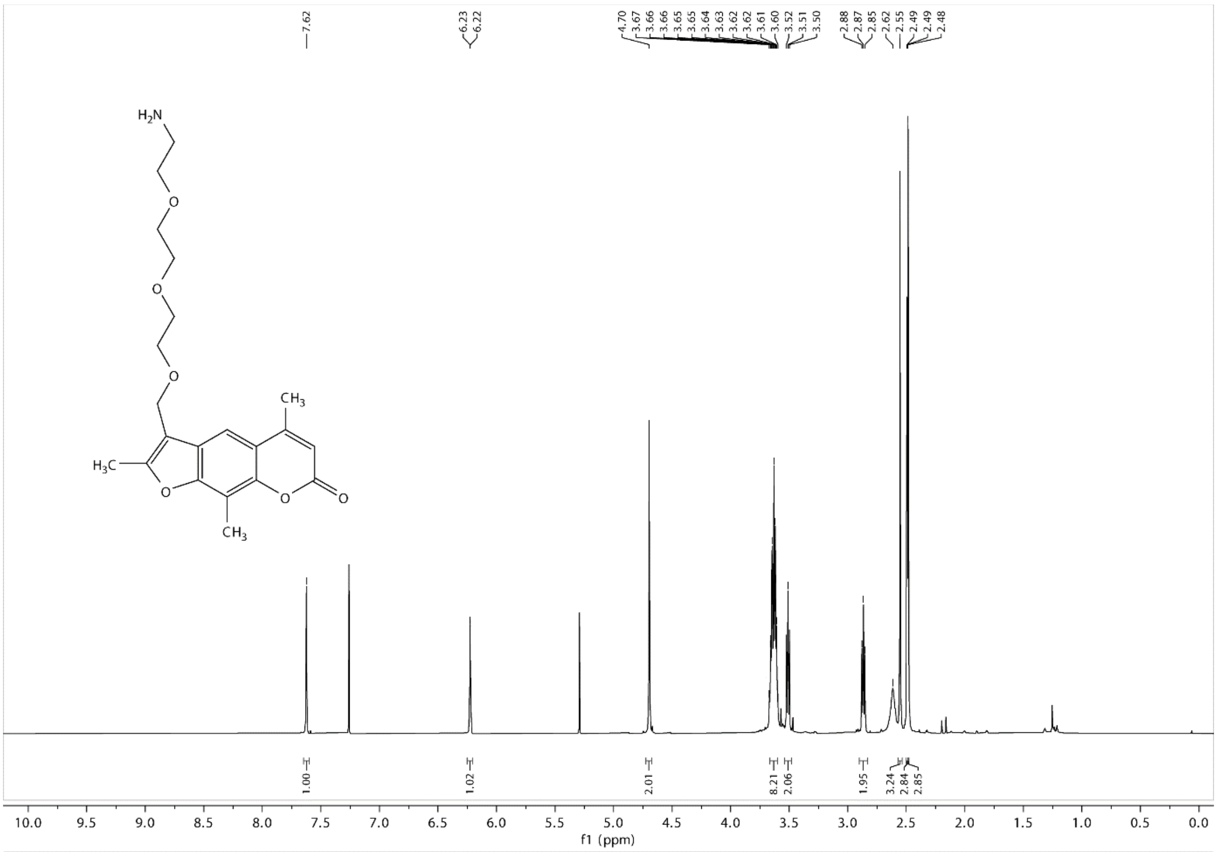

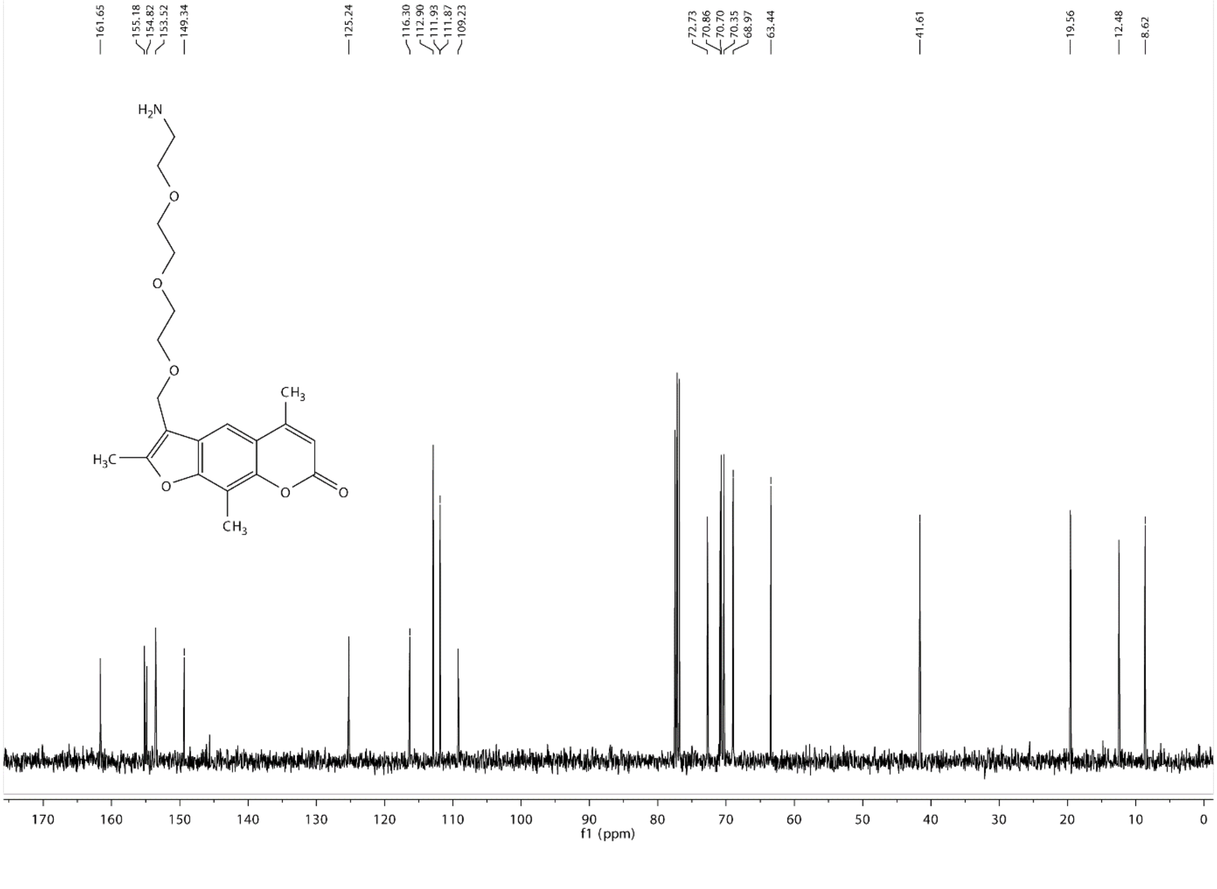

^{1}H and ^{13}C NMR (Chloroform-*d*) spectra of the compound **2d**

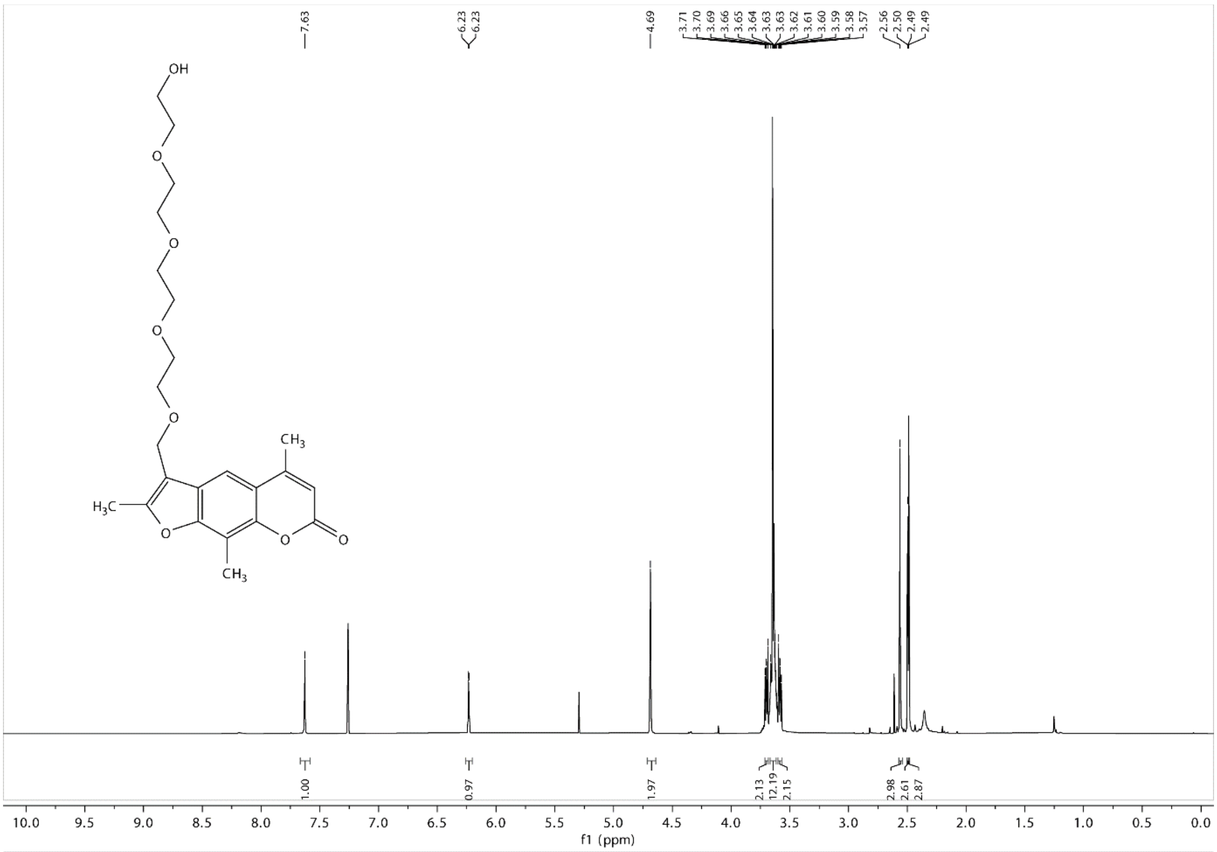

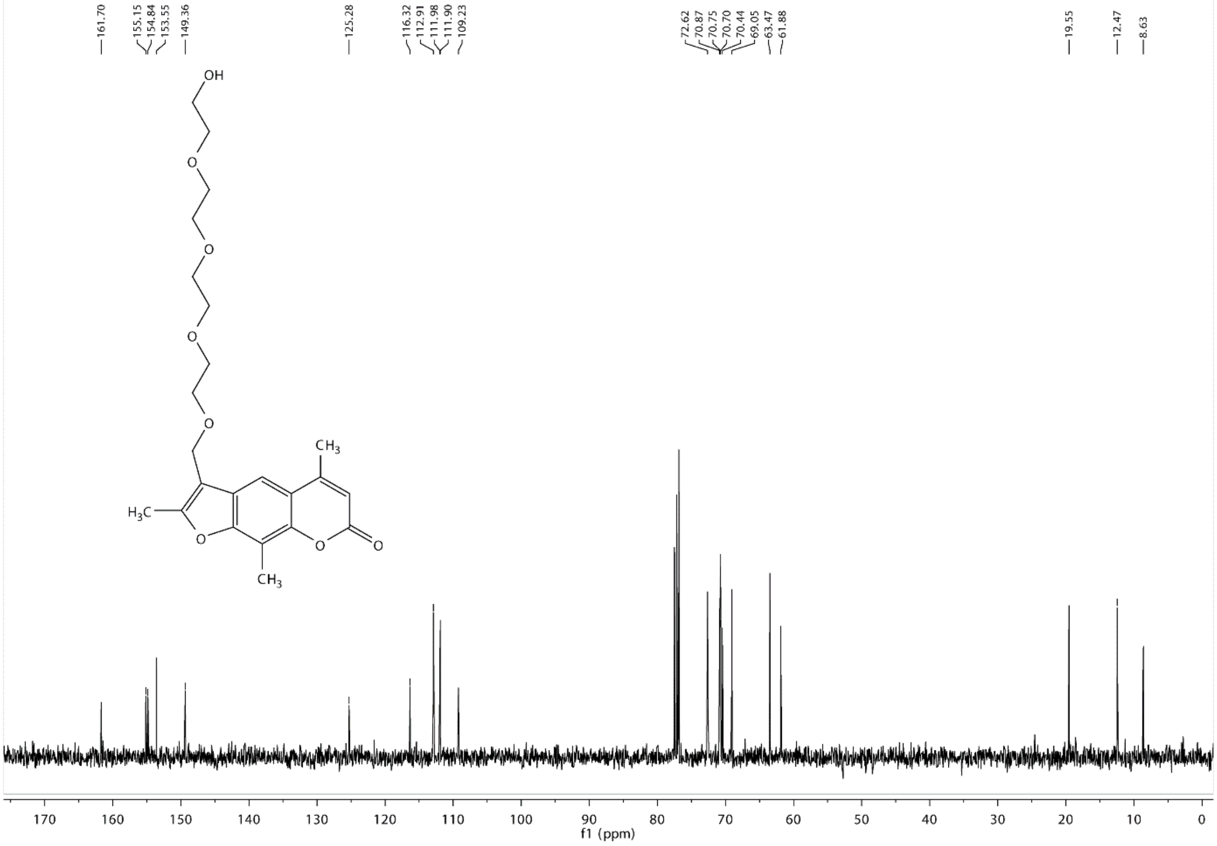

^{1}H and ^{13}C NMR (Chloroform-*d*) spectra of the compound **3d**

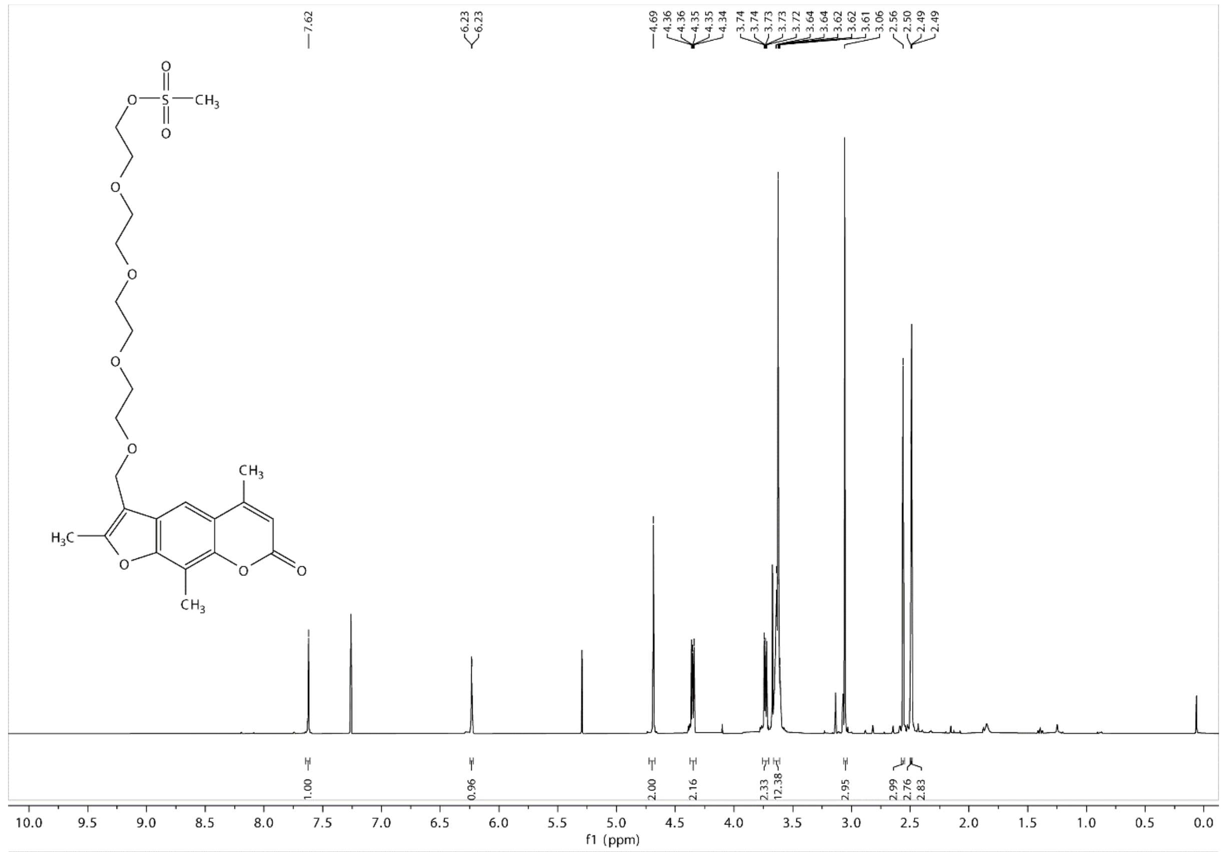

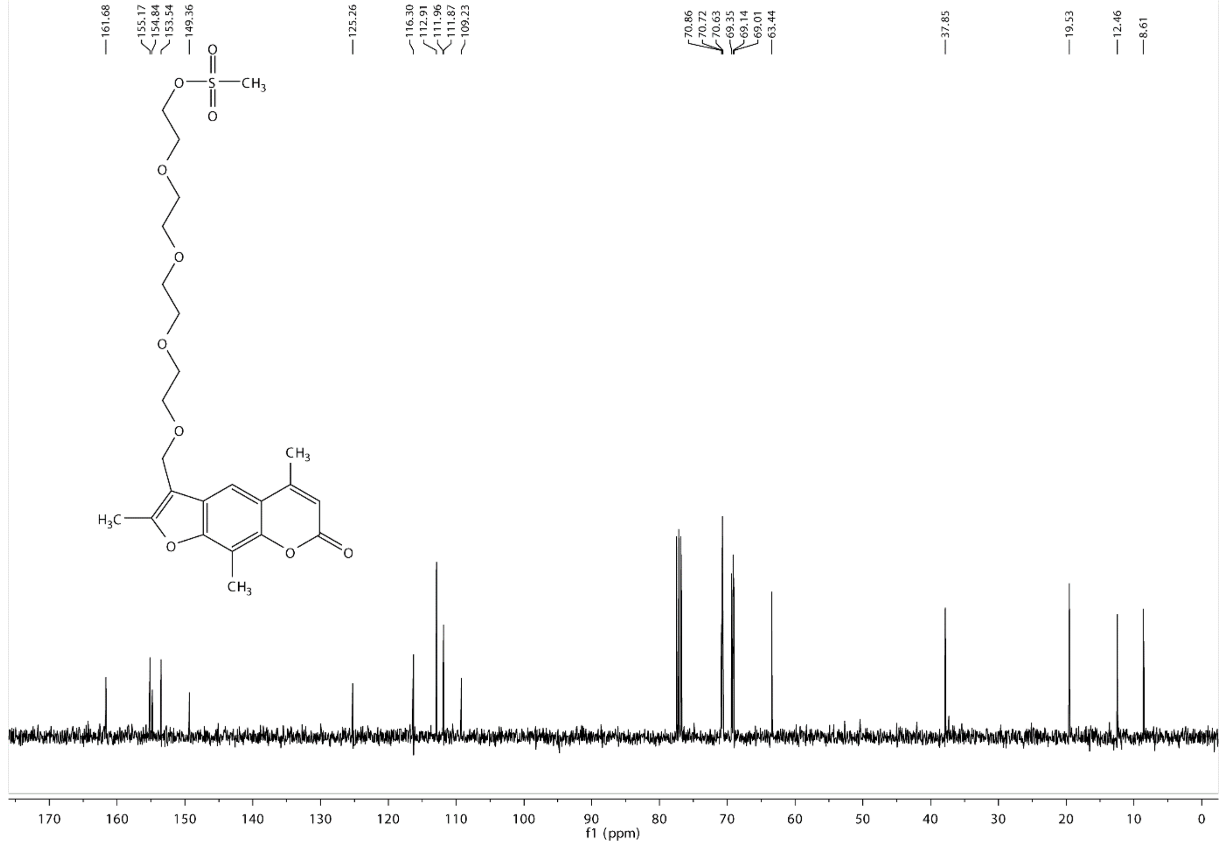

^{1}H and ^{13}C NMR (Chloroform-*d*) spectra of the compound **4d**

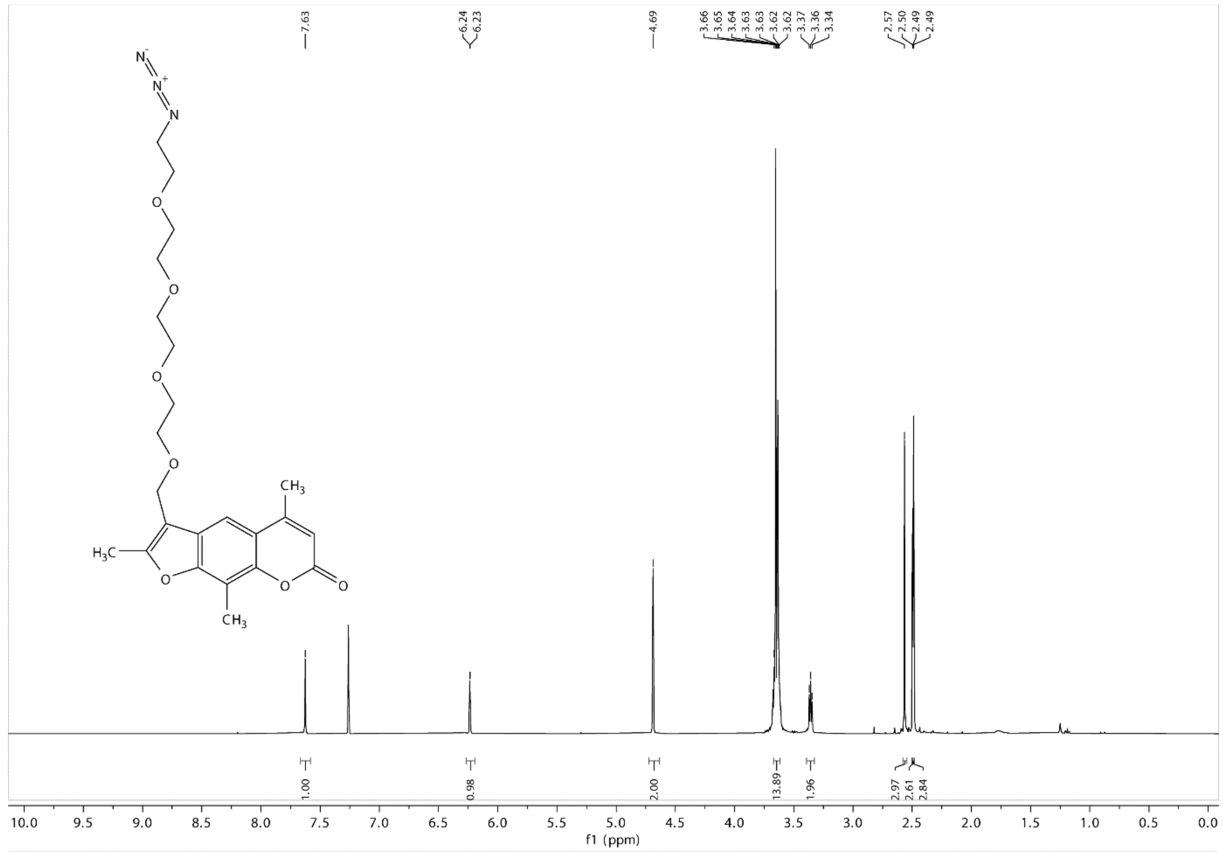

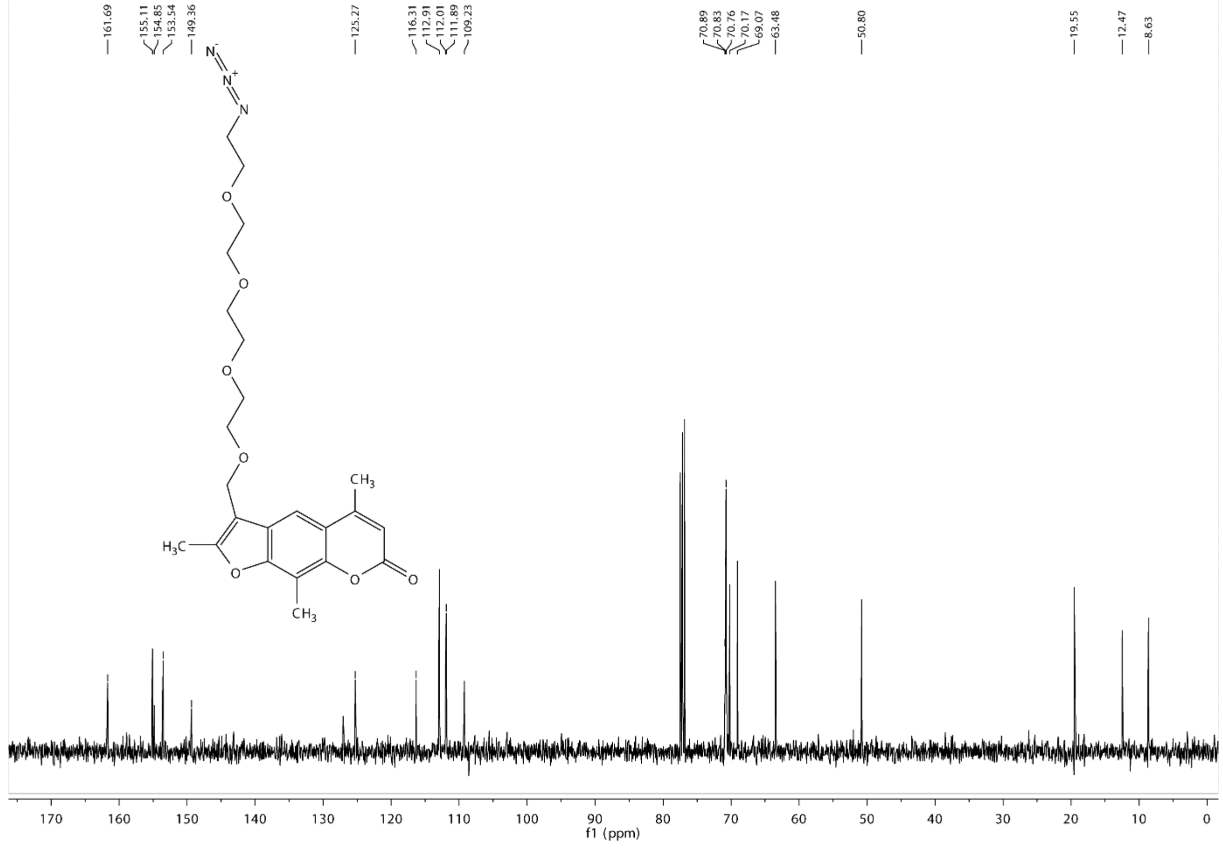

^{1}H and ^{13}C NMR (Chloroform-*d*) spectra of the compound **5d**

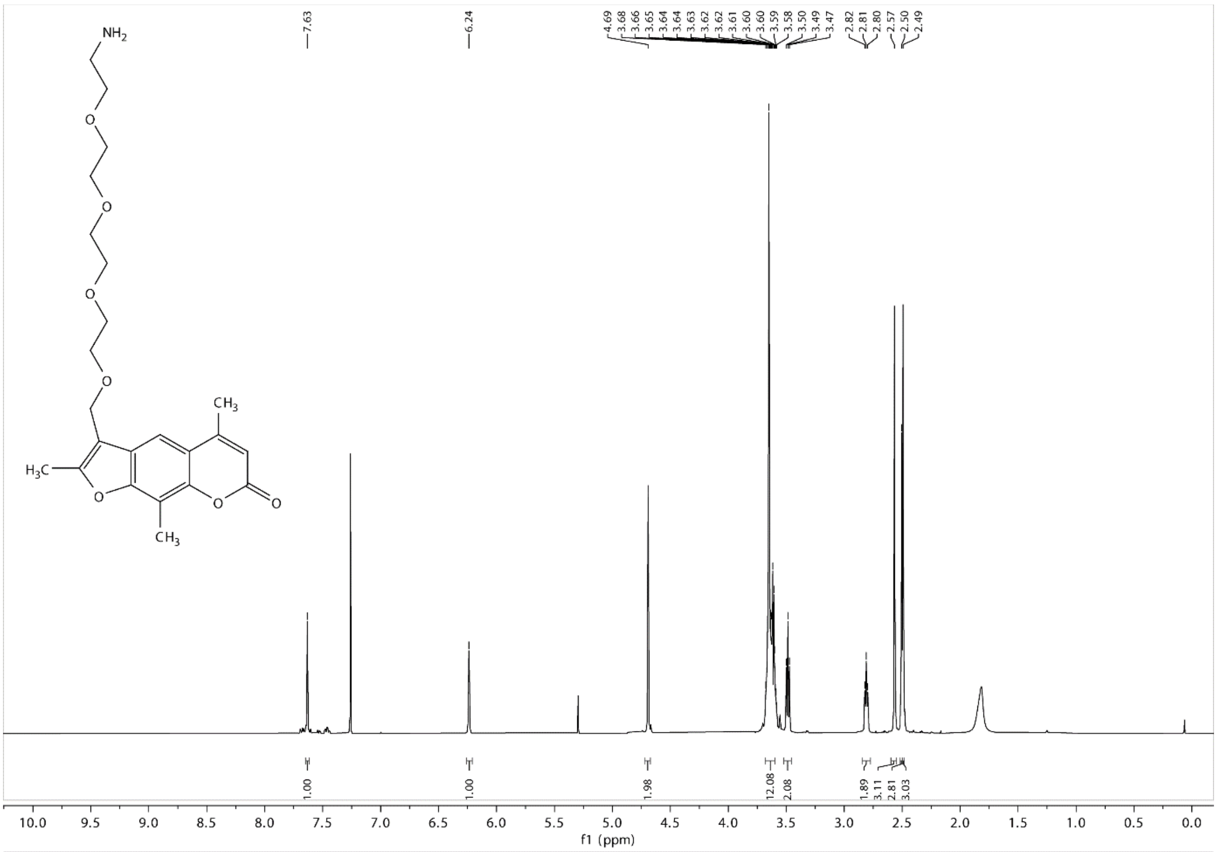

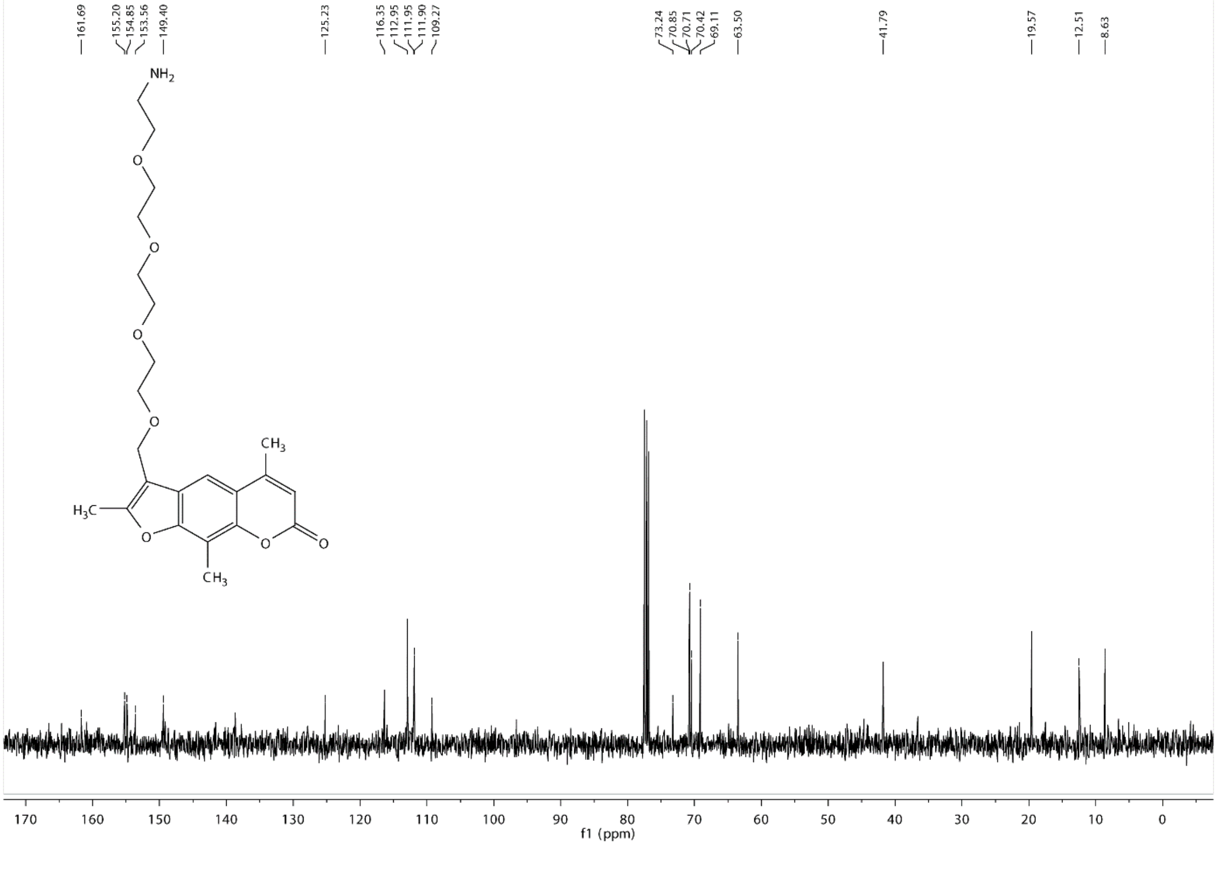

III.2. LC-MS chromatograms of ORNs

Table S1: Characterization of the ORNs used in this study. **Y**: adenosine modified with trioxsalen attached at the 2'-*O*-position; **X**: cytidine with the trioxsalen attached at the N^4 position, the subscript (0-4) indicates the number of ethylene glycol (EG) units in the linker.

Entry	ORN	Sequence (5' to 3')	Length	Mass calc.	Mass found	Δm (%)	UV Purity [%]	Synthesis scale
		**miR-124**						
1	ORN-1	UAAGGCACGCGGUG**Y**AUGCC	20	6766.4	6765.5	0.01	>98	50 nmol
2	ORN-2	UCACCGCGUGCCUUA	15	4687.9	4687.1	0.02	>98	1 µM
3	ORN-3	UA**Y**GGCACGCGGUGAAUGCC	20	6766.4	6765.4	0.01	>98	50 nmol
4	ORN-4	UAAGGCACGCGGUGAAUGCC	20	6445.0	6444.7	0.00	>95	50 nmol
5	ORN-4a	UAAGG$\mathbf{X}_0$ACGCGGUGAAUGCC	20	6685.3	6684.2	0.02	>98	1 µM
6	ORN-4b	UAAGG$\mathbf{X}_1$ACGCGGUGAAUGCC	20	6729.3	6728.3	0.01	>98	1 µM
7	ORN-4c	UAAGG$\mathbf{X}_2$ACGCGGUGAAUGCC	20	6773.4	6772.3	0.02	>98	1 µM
8	ORN-4d	UAAGG$\mathbf{X}_3$ACGCGGUGAAUGCC	20	6817.4	6816.4	0.01	>98	1 µM
9	ORN-4e	UAAGG$\mathbf{X}_4$ACGCGGUGAAUGCC	20	6861.5	6860.4	0.02	>98	1 µM
10	ORN-5a	UAAGGCA$\mathbf{X}_1$GCGGUGAAUGCC	20	6729.3	6728.6	0.01	>95	1 µM
11	ORN-5b	UAAGGCA$\mathbf{X}_2$GCGGUGAAUGCC	20	6773.4	6772.7	0.01	>98	1 µM
12	ORN-5c	UAAGGCA$\mathbf{X}_3$GCGGUGAAUGCC	20	6817.4	6816.6	0.01	>98	1 µM
13	ORN-5d	UAAGGCA$\mathbf{X}_4$GCGGUGAAUGCC	20	6861.5	6860.8	0.01	>98	1 µM
		**miR-10b**						
14	ORN-6a	UAC$\mathbf{X}_1$CUGUAGAACCGAAUUUGUG	23	7592.7	7592.0	0.01	>95	1 µM
15	ORN-6b	UAC$\mathbf{X}_2$CUGUAGAACCGAAUUUGUG	23	7636.8	7635.9	0.01	>98	1 µM
16	ORN-6c	UAC$\mathbf{X}_3$CUGUAGAACCGAAUUUGUG	23	7680.8	7680.1	0.01	>95	1 µM
17	ORN-6d	UAC$\mathbf{X}_4$CUGUAGAACCGAAUUUGUG	23	7724.9	7724.1	0.01	>98	1 µM
18	ORN-7	CGGUUCUACAGGGUA	15	4791.9	4791.3	0.01	>95	1 µM
		**miR-146a**						
19	ORN-8a	UGAGAA$\mathbf{X}_1$UGAAUUCCAUGGGUU	22	7327.6	7326.8	0.01	>90	1 µM
20	ORN-8b	UGAGAA$\mathbf{X}_2$UGAAUUCCAUGGGUU	22	7371.7	7370.8	0.01	>90	1 µM
21	ORN-8c	UGAGAA$\mathbf{X}_3$UGAAUUCCAUGGGUU	22	7415.7	7414.9	0.01	>87	1 µM
22	ORN-8d	UGAGAA$\mathbf{X}_4$UGAAUUCCAUGGGUU	22	7459.8	7458.9	0.01	>84	1 µM
23	ORN-9	GGAAUUCAGUUCUCA	15	4736.9	4736.3	0.01	>95	1 µM
		**miR-208a**						
24	ORN-10a	AUAAGA$\mathbf{X}_1$GAGCAAAAAGCUUGU	22	7380.7	7380.2	0.01	>98	1 µM
25	ORN-10b	AUAAGA$\mathbf{X}_2$GAGCAAAAAGCUUGU	22	7424.8	7424.2	0.01	>98	1 µM
26	ORN-10c	AUAAGA$\mathbf{X}_3$GAGCAAAAAGCUUGU	22	7468.8	7468.0	0.01	>98	1 µM
27	ORN-10d	AUAAGA$\mathbf{X}_4$GAGCAAAAAGCUUGU	22	7512.9	7512.2	0.01	>98	1 µM
28	ORN-11	UUUUGCUCGUCUUAU	22	4628.7	4628.1	0.01	>90	1 µM
		**miR-155-3p**						
29	ORN-12a	CUCCUACAUAUUAG$\mathbf{X}_1$AUUAACA	22	7175.5	7174.7	0.01	>86	1 µM
30	ORN-12b	CUCCUACAUAUUAG$\mathbf{X}_2$AUUAACA	22	7219.6	7218.8	0.01	>90	1 µM
31	ORN-12c	CUCCUACAUAUUAG$\mathbf{X}_3$AUUAACA	22	7263.6	7262.7	0.01	>86	1 µM
32	ORN-12d	CUCCUACAUAUUAG$\mathbf{X}_4$AUUAACA	22	7307.7	7306.9	0.01	>98	1 µM
33	ORN-13	GCUAAUAUGUAGGAG	15	4840.0	4839.3	0.01	>94	1 µM

III.2.1. miR-124 probes

ORN-1

Additional Info : Peak(s) manually integrated

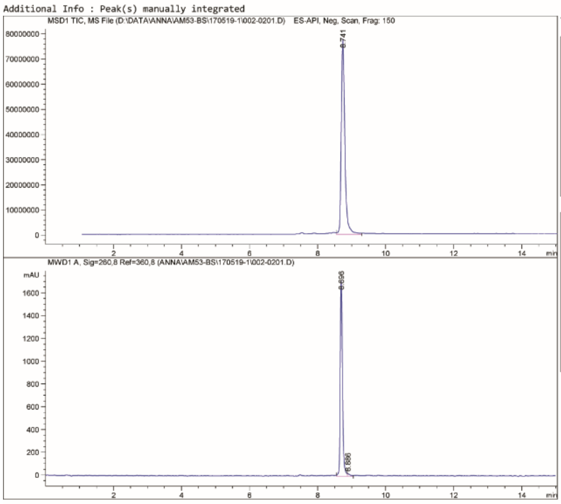

Deconvolution of Spectrum # 1 @ 8.721 - 8.796 min

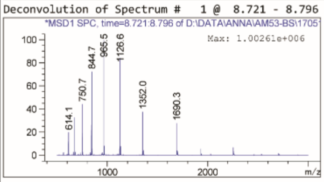

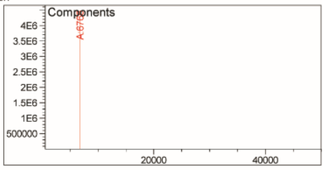

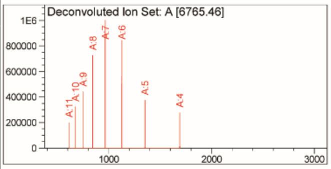

Signal 2: MWD1 A, Sig=260,8 Ref=360,8

Peak #	RetTime [min]	Type	Width [min]	Area [mAU*s]	Height [mAU]	Area %
1	8.696	VV	0.0725	8024.78906	1718.24048	98.3169
2	8.886	VV	0.1028	137.37480	22.26745	1.6831

Totals : 8162.16386 1740.50793

Component	Molecular Weight	Absolute Abundance	Relative Abundance
A	6765.46	4157702	100.00

*** End of Report ***

ORN-2

Additional Info : Peak(s) manually integrated

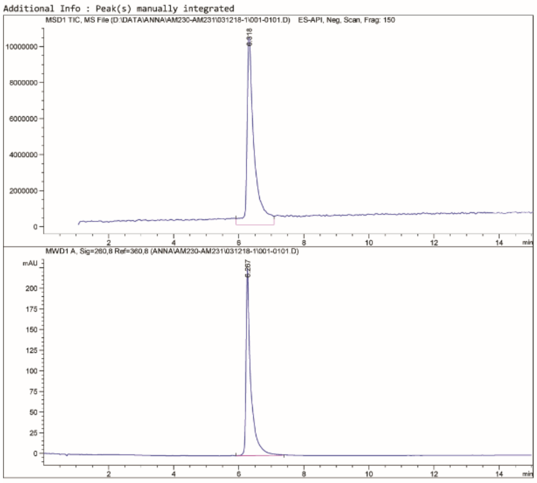

Deconvolution of Spectrum # 1 @ 6.280 - 6.379 min

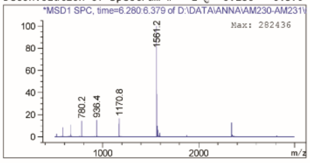

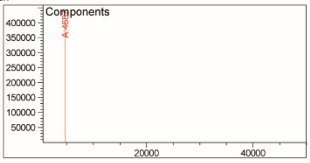

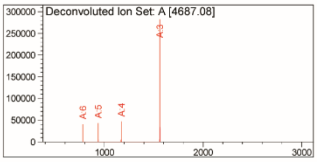

Signal 2: MWD1 A, Sig=260,8 Ref=360,8

Peak #	RetTime [min]	Type	Width [min]	Area [mAU*s]	Height [mAU]	Area %
1	6.267	VV	0.1456	2366.91577	227.14522	100.0000

Totals : 2366.91577 227.14522

Component	Molecular Weight	Absolute Abundance	Relative Abundance
A	4687.08	410143	100.00

*** End of Report ***

ORN-3

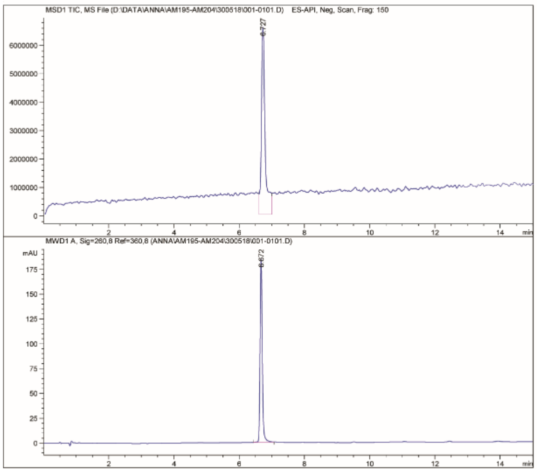

Deconvolution of Spectrum # 1 @ 6.668 - 6.768 min

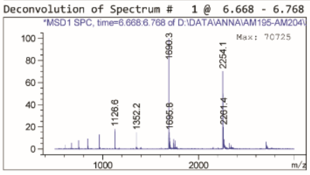

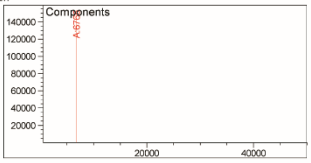

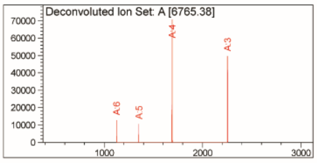

Signal 2: MWD1 A, Sig=260,8 Ref=360,8

Peak #	RetTime [min]	Type	Width [min]	Area [mAU*s]	Height [mAU]	Area %
1	6.672	VB	0.0722	859.97015	185.19011	100.0000

Totals : 859.97015 185.19011

Component	Molecular Weight	Absolute Abundance	Relative Abundance
A	6765.38	142267	100.00

*** End of Report ***

ORN-4

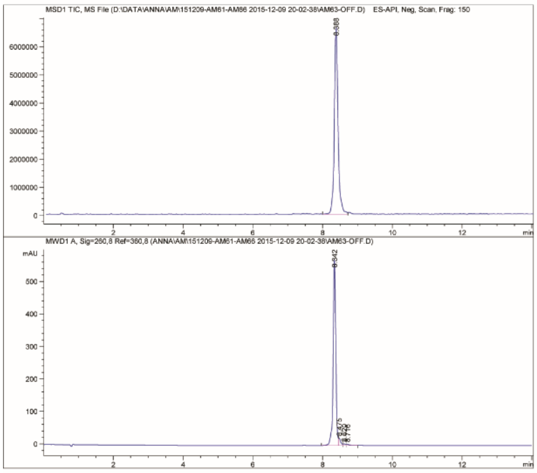

Deconvolution of Spectrum # 1 @ 8.345 - 8.445 min

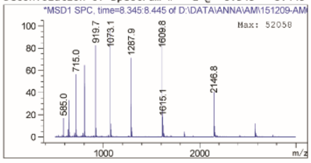

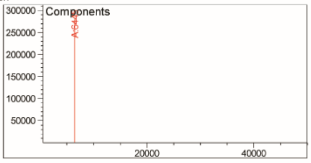

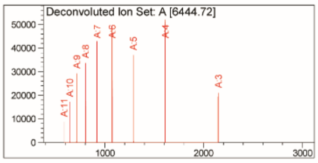

Signal 2: MWD1 A, Sig=260,8 Ref=360,8

Peak #	RetTime [min]	Type	Width [min]	Area [mAU*s]	Height [mAU]	Area %
1	8.342	VV	0.0782	2971.09619	575.47180	95.5399
2	8.475	VV	0.0620	91.83524	22.30883	2.9531
3	8.620	VV	0.0744	23.94691	4.62190	0.7700
4	8.716	VB	0.0896	22.91724	3.63323	0.7369
Totals :				3109.79559	606.03577	

Component	Molecular Weight	Absolute Abundance	Relative Abundance
A	6444.72	279376	100.00

*** End of Report ***

ORN-4a

Additional Info : Peak(s) manually integrated

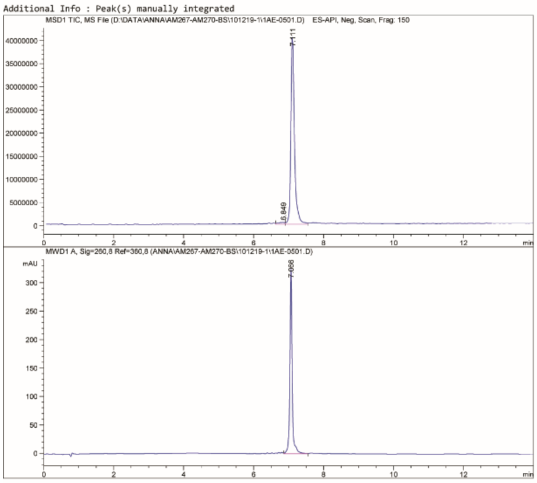

Deconvolution of Spectrum # 1 @ 7.051 - 7.126 min

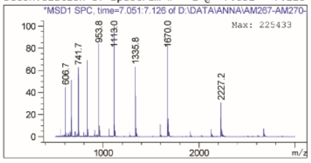

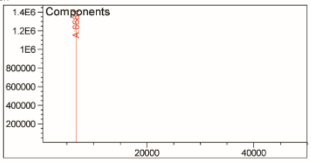

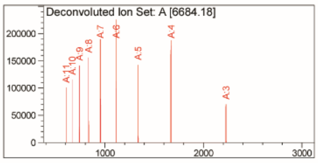

Signal 2: MWD1 A, Sig=260,8 Ref=360,8

Peak #	RetTime [min]	Type	Width [min]	Area [mAU*s]	Height [mAU]	Area %
1	7.066	VV	0.0691	1536.24744	325.18970	100.0000
Totals :				1536.24744	325.18970	

Component	Molecular Weight	Absolute Abundance	Relative Abundance
A	6684.18	1320326	100.00

*** End of Report ***

ORN-4b

Additional Info : Peak(s) manually integrated

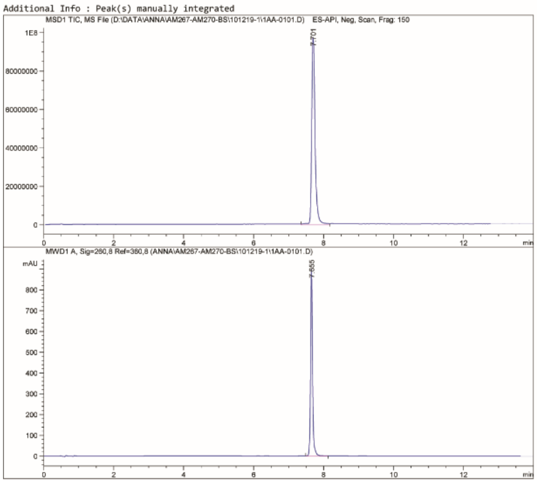

Deconvolution of Spectrum # 1 @ 7.641 - 7.741 min

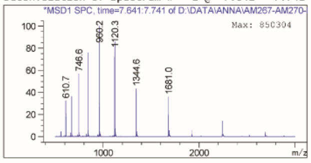

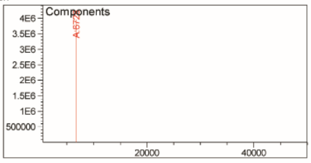

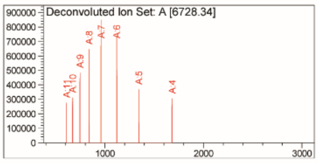

Signal 2: MWD1 A, Sig=260,8 Ref=360,8

Peak #	RetTime [min]	Type	Width [min]	Area [mAU*s]	Height [mAU]	Area %
1	7.655	VB	0.0621	3711.53418	900.64197	100.0000
Totals :				3711.53418	900.64197	

Component	Molecular Weight	Absolute Abundance	Relative Abundance
A	6728.34	3949301	100.00

*** End of Report ***

ORN-4c

Additional Info : Peak(s) manually integrated

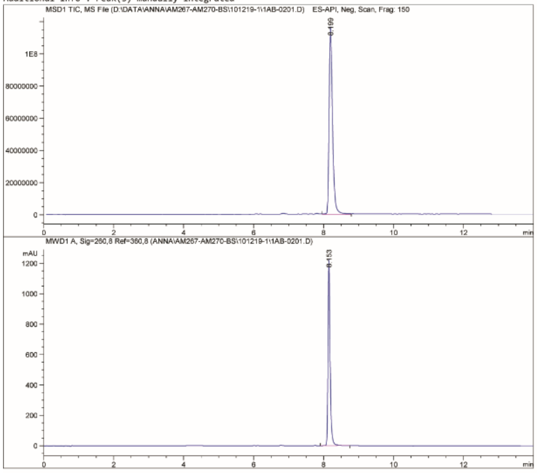

Deconvolution of Spectrum # 1 @ 8.172 - 8.247 min

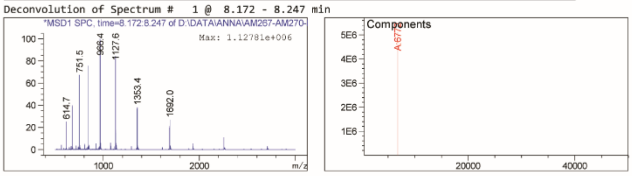

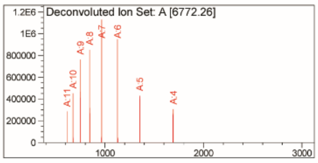

Signal 2: MWD1 A, Sig=260,8 Ref=360,8

Peak #	RetTime [min]	Type	Width [min]	Area [mAU*s]	Height [mAU]	Area %
1	8.153	VB	0.0629	5157.27393	1230.21228	100.0000

Totals : 5157.27393 1230.21228

Component	Molecular Weight	Absolute Abundance	Relative Abundance
A	6772.26	5111062	100.00

*** End of Report ***

ORN-4d

Additional Info : Peak(s) manually integrated

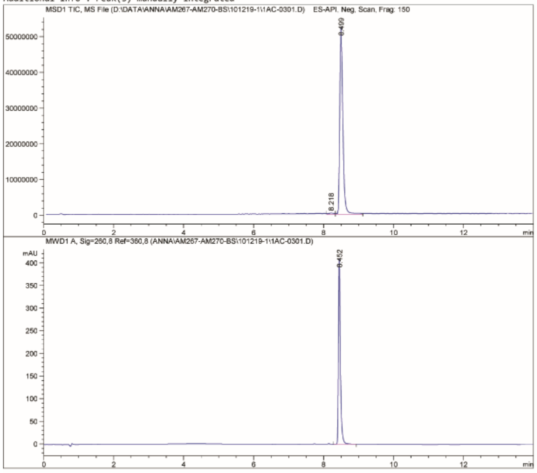

Deconvolution of Spectrum # 1 @ 8.446 - 8.546 min

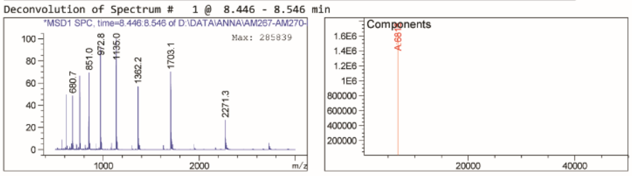

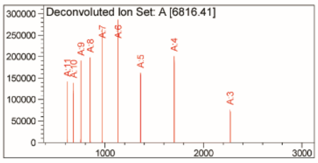

Signal 2: MWD1 A, Sig=260,8 Ref=360,8

Peak #	RetTime [min]	Type	Width [min]	Area [mAU*s]	Height [mAU]	Area %
1	8.452	VB	0.0599	1621.26416	412.39468	100.0000

Totals : 1621.26416 412.39468

Component	Molecular Weight	Absolute Abundance	Relative Abundance
A	6816.41	1648395	100.00

*** End of Report ***

ORN-4e

Additional Info : Peak(s) manually integrated

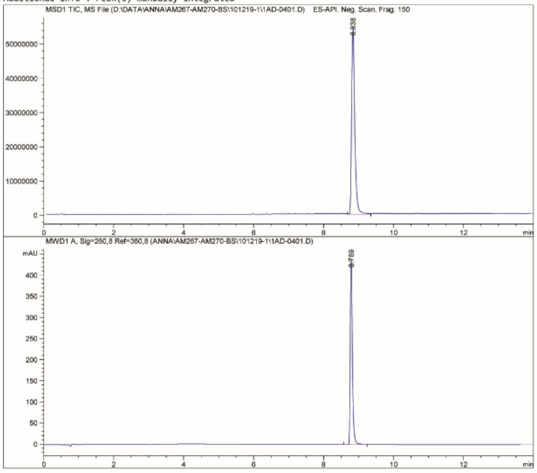

Deconvolution of Spectrum # 1 @ 8.770 - 8.920 min

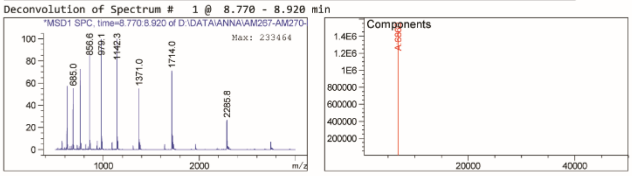

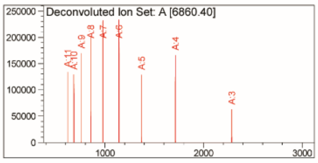

Signal 2: MWD1 A, Sig=260,8 Ref=360,8

Peak #	RetTime [min]	Type	Width [min]	Area [mAU*s]	Height [mAU]	Area %
1	8.789	VB	0.0617	1804.46399	441.13565	100.0000

Totals : 1804.46399 441.13565

Component	Molecular Weight	Absolute Abundance	Relative Abundance
A	6860.40	1445699	100.00

*** End of Report ***

ORN-5a

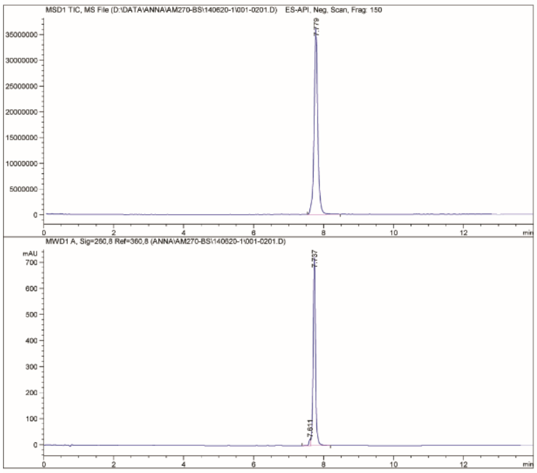

Deconvolution of Spectrum # 1 @ 7.749 - 7.798 min

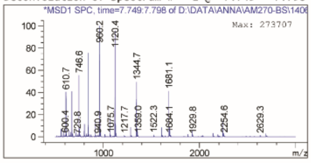

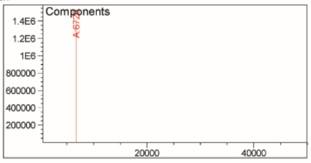

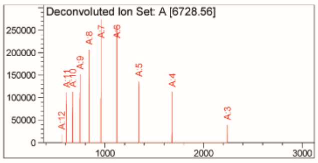

Signal 2: MWD1 A, Sig=260,8 Ref=360,8

Peak #	RetTime [min]	Type	Width [min]	Area [mAU*s]	Height [mAU]	Area %
1	7.611	BV	0.0482	73.22569	24.22280	2.1543
2	7.737	VB	0.0699	3325.84839	720.46552	97.8457
Totals :				3399.07407	744.68832	

Component	Molecular Weight	Absolute Abundance	Relative Abundance
A	6728.56	1418854	100.00

*** End of Report ***

ORN-5b

Additional Info : Peak(s) manually integrated

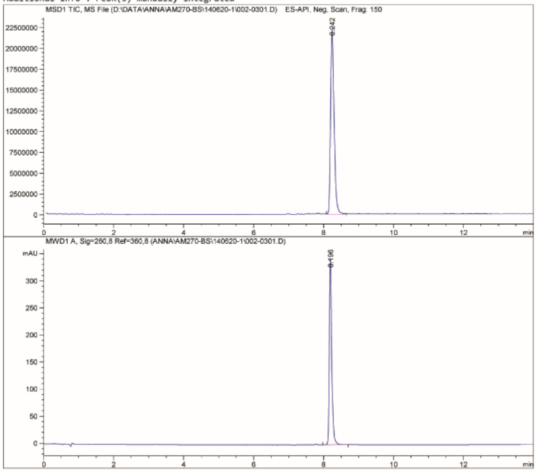

Deconvolution of Spectrum # 1 @ 8.172 - 8.297 min

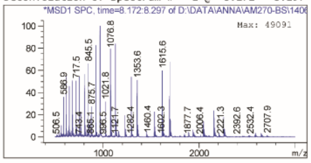

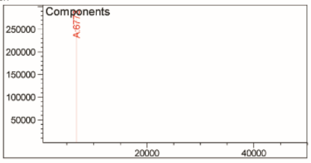

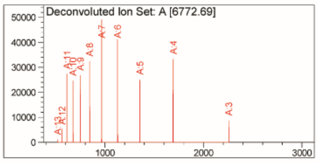

Signal 2: MWD1 A, Sig=260,8 Ref=360,8

Peak #	RetTime [min]	Type	Width [min]	Area [mAU*s]	Height [mAU]	Area %
1	8.196	BB	0.0682	1600.24524	344.32343	100.0000
Totals :				1600.24524	344.32343	

Component	Molecular Weight	Absolute Abundance	Relative Abundance
A	6772.69	271425	100.00

*** End of Report ***

ORN-5c

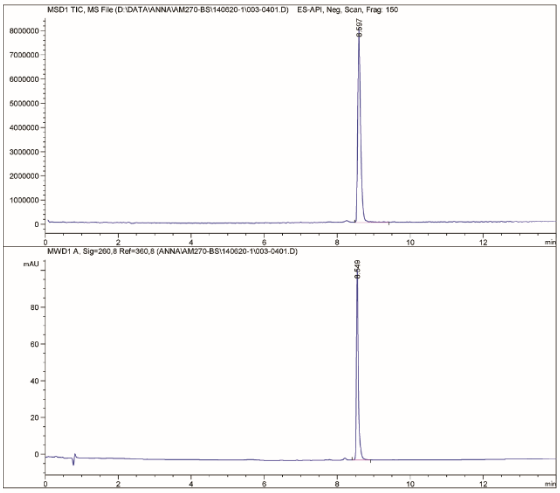

Deconvolution of Spectrum # 1 @ 8.546 - 8.621 min

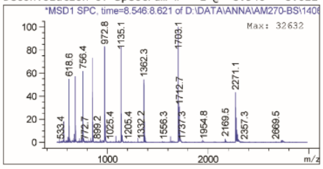

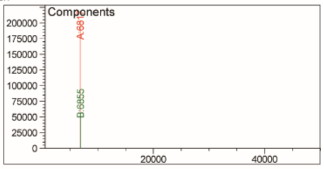

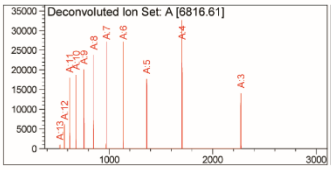

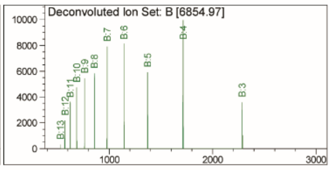

Component	Molecular Weight	Absolute Abundance	Relative Abundance
A	6816.61	204315	100.00
B	6854.97	56453	27.63

*** End of Report ***

Signal 2: MWD1 A, Sig=260,8 Ref=360,8

Peak #	RetTime [min]	Type	Width [min]	Area [mAU*s]	Height [mAU]	Area %
1	8.549	BB	0.0581	392.44409	103.89192	100.0000
Totals :				392.44409	103.89192	

ORN-5d

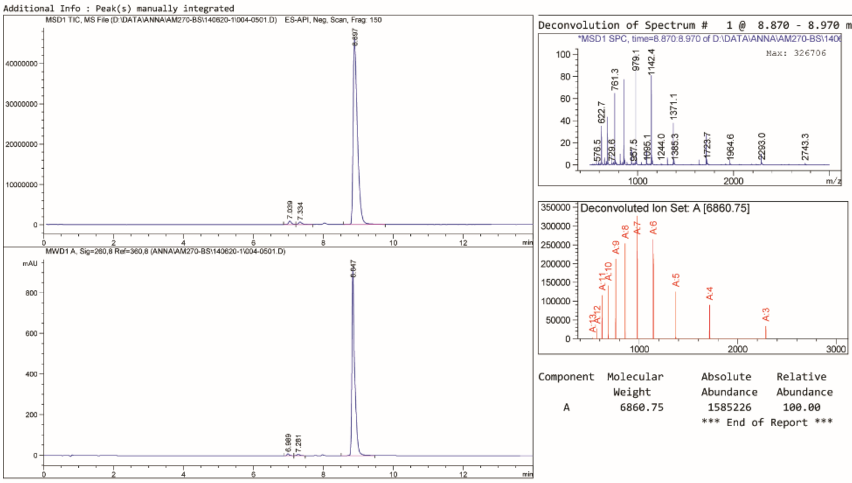

Signal 2: MWD1 A, Sig=260,8 Ref=360,8

Peak #	RetTime [min]	Type	Width [min]	Area [mAU*s]	Height [mAU]	Area %
1	6.989	VV	0.0696	47.08147	9.53201	0.8839
2	7.281	VV	0.1141	49.83645	6.60215	0.9356
3	8.847	VB	0.0799	5229.59424	925.45569	98.1805

Totals : 5326.51217 941.58986

Component	Molecular Weight	Absolute Abundance	Relative Abundance
A	6860.75	1585226	100.00

*** End of Report ***

III.2.2. miR-10b probes

ORN-6a

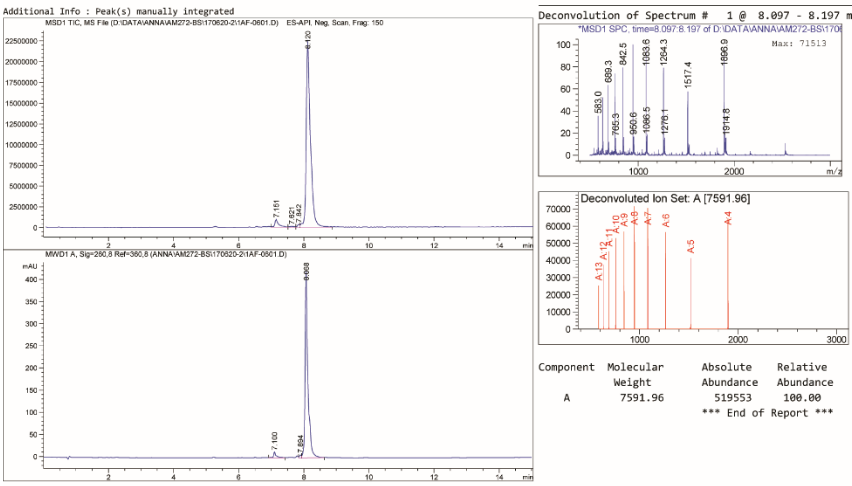

Signal 2: MWD1 A, Sig=260,8 Ref=360,8

Peak #	RetTime [min]	Type	Width [min]	Area [mAU*s]	Height [mAU]	Area %
1	7.100	VV	0.0869	84.01404	13.07767	3.2055
2	7.894	VV	0.0613	26.57286	6.55721	1.0139
3	8.068	VV	0.0835	2510.36938	421.19711	95.7807

Totals : 2620.95628 440.83200

Component	Molecular Weight	Absolute Abundance	Relative Abundance
A	7591.96	519553	100.00

*** End of Report ***

ORN-6b

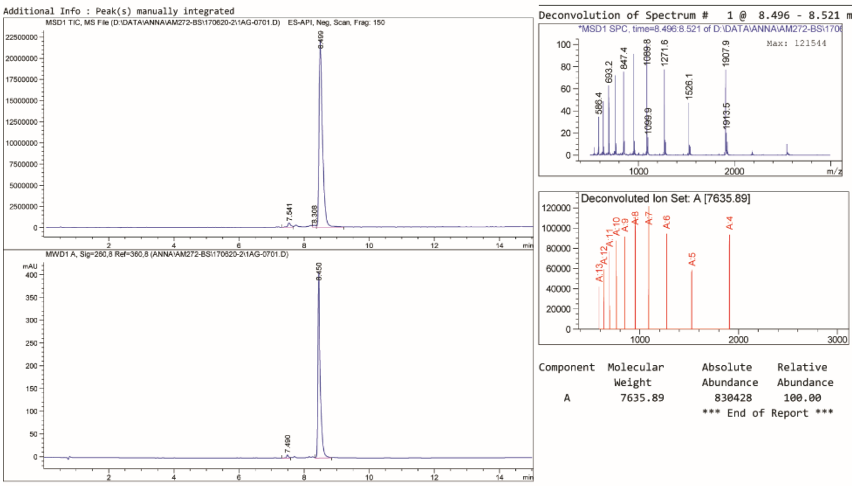

Signal 2: MWD1 A, Sig=260,8 Ref=360,8

Peak #	RetTime [min]	Type	Width [min]	Area [mAU*s]	Height [mAU]	Area %
1	7.490	VV	0.0617	30.20381	7.08492	1.5098
2	8.450	VV	0.0699	1970.37866	411.26752	98.4902

Totals : 2000.58247 418.35244

Component	Molecular Weight	Absolute Abundance	Relative Abundance
A	7635.89	830428	100.00

*** End of Report ***

ORN-6c

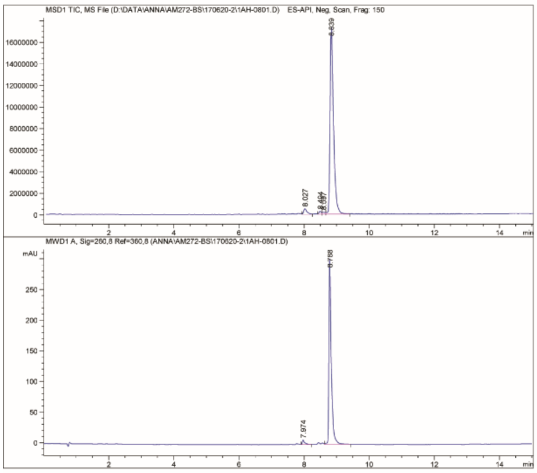

Deconvolution of Spectrum # 1 @ 8.820 - 8.895 min

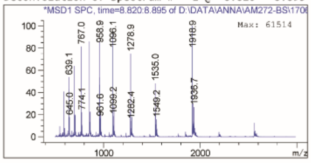

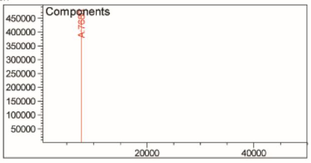

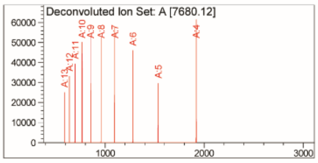

Signal 2: MWD1 A, Sig=260,8 Ref=360,8

Peak #	RetTime [min]	Type	Width [min]	Area [mAU*s]	Height [mAU]	Area %
1	7.974	VB	0.0733	36.37839	7.15133	2.3429
2	8.788	VB	0.0722	1516.33679	303.80469	97.6571
Totals :				1552.71518	310.95602	

Component	Molecular Weight	Absolute Abundance	Relative Abundance
A	7680.12	445257	100.00

*** End of Report ***

ORN-6d

Additional Info : Peak(s) manually integrated

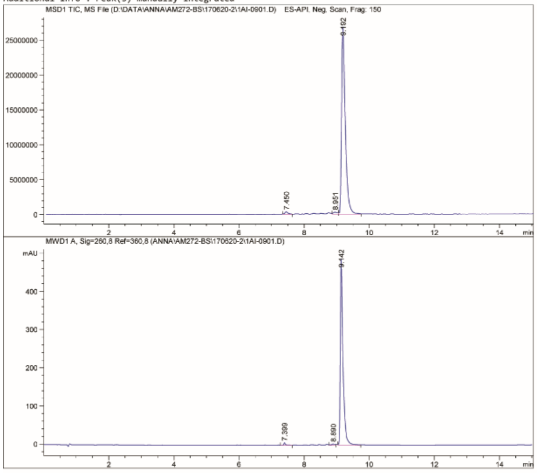

Deconvolution of Spectrum # 1 @ 9.169 - 9.269 min

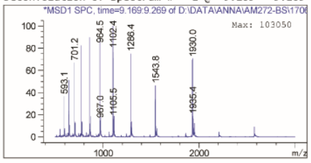

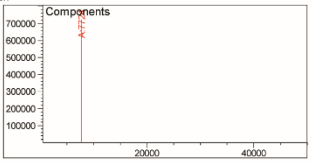

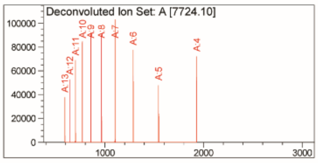

Signal 2: MWD1 A, Sig=260,8 Ref=360,8

Peak #	RetTime [min]	Type	Width [min]	Area [mAU*s]	Height [mAU]	Area %
1	7.399	VV	0.0629	27.25560	6.24112	0.9922
2	8.890	VV	0.1153	26.50154	3.11286	0.9647
3	9.142	VV	0.0783	2693.32251	488.80478	98.0431
Totals :				2747.07964	498.15876	

Component	Molecular Weight	Absolute Abundance	Relative Abundance
A	7724.10	722895	100.00

*** End of Report ***

ORN-7

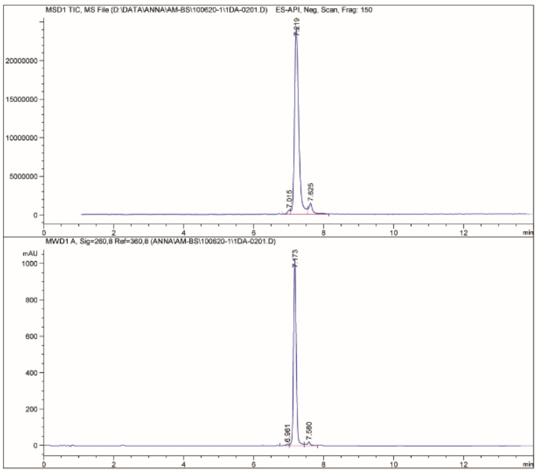

Deconvolution of Spectrum # 1 @ 7.178 - 7.277 min

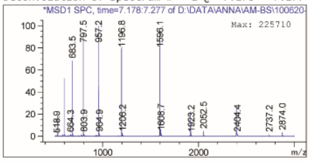

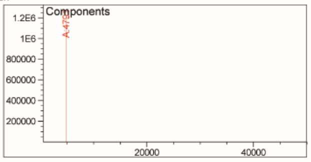

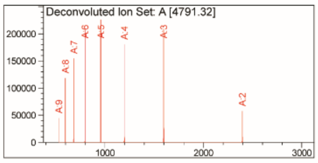

Signal 2: MWD1 A, Sig=260,8 Ref=360,8

Peak #	RetTime [min]	Type	Width [min]	Area [mAU*s]	Height [mAU]	Area %
1	6.961	VV	0.0829	51.31551	8.68632	0.9088
2	7.173	VV	0.0817	5437.85889	1027.95996	96.3031
3	7.580	VV	0.1002	157.43617	21.30200	2.7882
Totals :				5646.61057	1057.94828	

Component	Molecular Weight	Absolute Abundance	Relative Abundance
A	4791.32	1185089	100.00

*** End of Report ***

III.2.3. miR-146a probes

For this series, small amounts of unidentified faster-running side peaks accompanied the product peaks. It was not possible to remove these by double purification and cross-linking experiments were performed with them present in the mixture.

ORN-8a

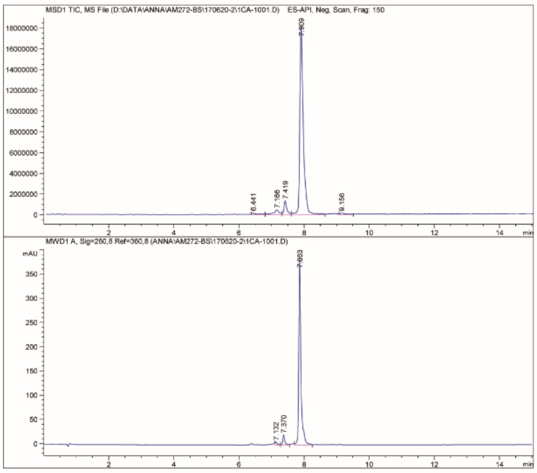

Deconvolution of Spectrum # 1 @ 7.848 - 7.973 min

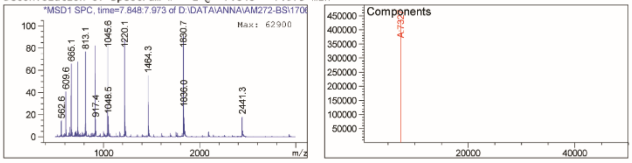

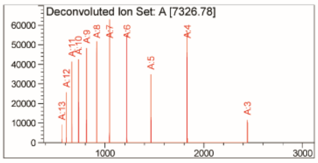

Signal 2: MWD1 A, Sig=260,8 Ref=360,8

Peak #	RetTime [min]	Type	Width [min]	Area [mAU*s]	Height [mAU]	Area %
1	7.132	VV	0.0711	32.61694	6.22303	1.6894
2	7.370	VV	0.0677	96.41830	20.94441	4.9941
3	7.863	VV	0.0687	1801.61267	384.25986	93.3165

Totals : 1930.64791 411.42730

Component	Molecular Weight	Absolute Abundance	Relative Abundance
A	7326.78	436723	100.00

*** End of Report ***

ORN-8b

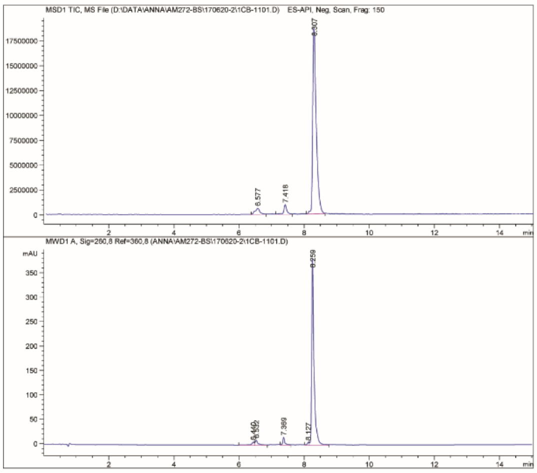

Deconvolution of Spectrum # 1 @ 8.247 - 8.372 min

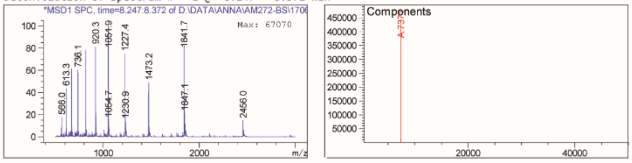

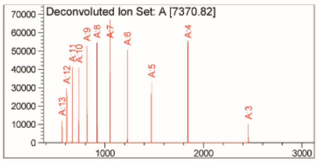

Signal 2: MWD1 A, Sig=260,8 Ref=360,8

Peak #	RetTime [min]	Type	Width [min]	Area [mAU*s]	Height [mAU]	Area %
1	6.440	BV	0.0817	38.41062	6.43217	1.8746
2	6.532	VV	0.0884	62.60902	9.81588	3.0555
3	7.369	VV	0.0699	76.74365	16.00655	3.7453
4	8.127	VV	0.0680	23.39929	5.05826	1.1420
5	8.259	VV	0.0682	1847.88733	383.65778	90.1826

Totals : 2049.04991 420.97062

Component	Molecular Weight	Absolute Abundance	Relative Abundance
A	7370.82	443790	100.00

*** End of Report ***

ORN-8c

Additional Info : Peak(s) manually integrated

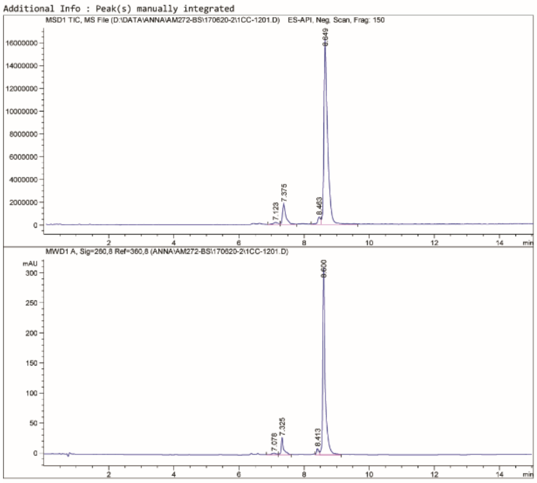

Deconvolution of Spectrum # 1 @ 8.621 - 8.720 min

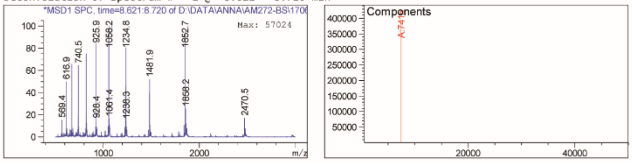

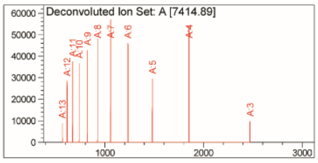

Signal 2: MWD1 A, Sig=260,8 Ref=360,8

Peak #	RetTime [min]	Type	Width [min]	Area [mAU*s]	Height [mAU]	Area %
1	7.078	BV	0.1316	32.46283	3.16553	1.7491
2	7.325	VV	0.0768	155.24025	28.82179	8.3643
3	8.413	VV	0.0697	50.54649	10.59477	2.7234
4	8.600	VV	0.0746	1617.73169	311.27939	87.1631

Totals : 1855.98125 353.86148

Component	Molecular Weight	Absolute Abundance	Relative Abundance
A	7414.89	395858	100.00

*** End of Report ***

ORN-8d

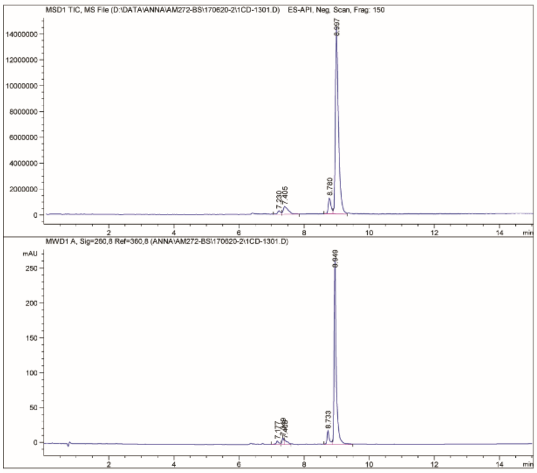

Deconvolution of Spectrum # 1 @ 8.944 - 9.044 min

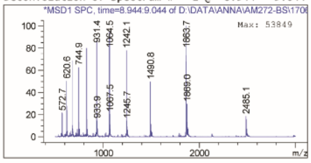

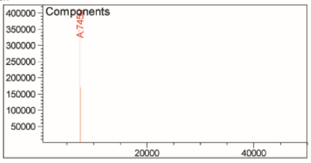

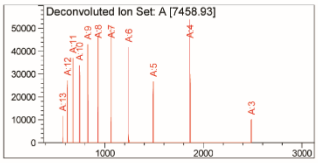

Signal 2: MWD1 A, Sig=260,8 Ref=360,8

Peak #	RetTime [min]	Type	Width [min]	Area [mAU*s]	Height [mAU]	Area %
1	7.177	BV	0.0866	32.79478	5.26700	2.3220
2	7.349	VV	0.0580	37.50160	9.52157	2.6553
3	7.408	VV	0.0939	43.34168	6.17537	3.0688
4	8.733	VV	0.0725	98.71464	19.67773	6.9894
5	8.949	VB	0.0666	1199.98962	266.33249	84.9645

Totals : 1412.34232 306.97416

Component	Molecular Weight	Absolute Abundance	Relative Abundance
A	7458.93	380033	100.00

*** End of Report ***

ORN-9

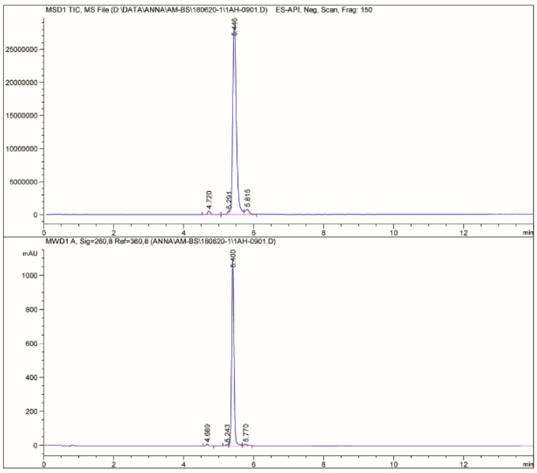

Deconvolution of Spectrum # 1 @ 5.382 - 5.482 min

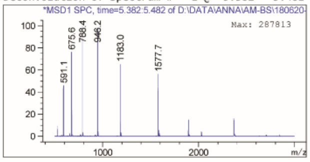

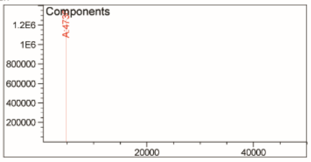

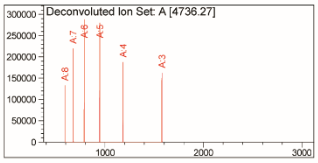

Signal 2: MWD1 A, Sig=260,8 Ref=360,8

Peak #	RetTime [min]	Type	Width [min]	Area [mAU*s]	Height [mAU]	Area %
1	4.669	VB	0.0631	40.28275	9.58104	0.7714
2	5.243	VV	0.0638	34.82380	8.15605	0.6669
3	5.400	VV	0.0718	5075.74609	1100.56287	97.2043
4	5.770	VV	0.1009	70.87978	9.51098	1.3574

Totals : 5221.73243 1127.81094

Component	Molecular Weight	Absolute Abundance	Relative Abundance
A	4736.27	1259001	100.00

*** End of Report ***

III.2.4. miR-208a probes

ORN-10a

Additional Info : Peak(s) manually integrated

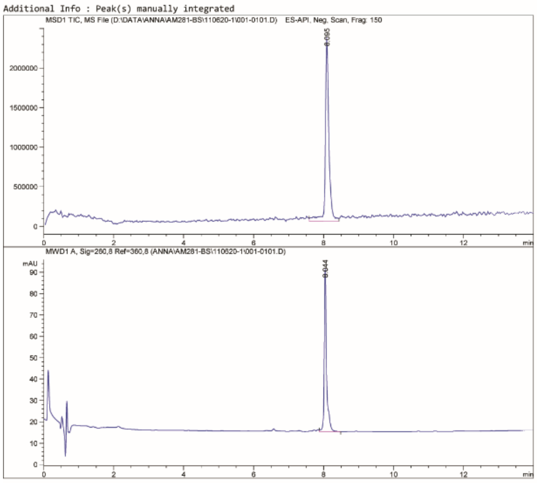

Deconvolution of Spectrum # 1 @ 8.064 - 8.139 min

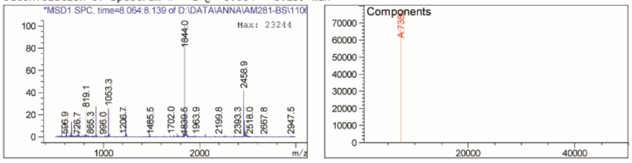

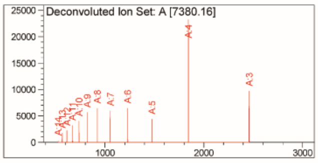

Signal 2: MWD1 A, Sig=260,8 Ref=360,8

Peak #	RetTime [min]	Type	Width [min]	Area [mAU*s]	Height [mAU]	Area %
1	8.044	VB	0.0643	341.52527	76.13747	100.0000
Totals :				341.52527	76.13747	

Component	Molecular Weight	Absolute Abundance	Relative Abundance
A	7380.16	72020	100.00

*** End of Report ***

ORN-10b

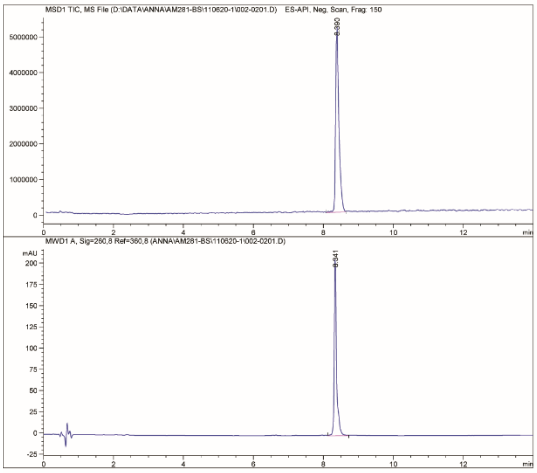

Deconvolution of Spectrum # 1 @ 8.347 - 8.446 min

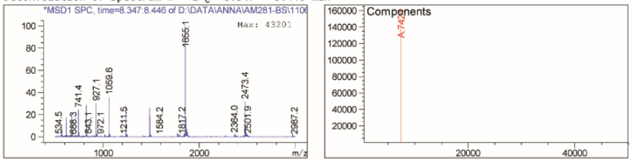

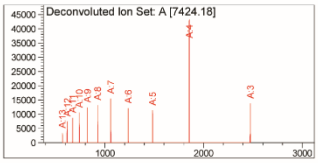

Signal 2: MWD1 A, Sig=260,8 Ref=360,8

Peak #	RetTime [min]	Type	Width [min]	Area [mAU*s]	Height [mAU]	Area %
1	8.341	VB	0.0637	925.18475	208.69975	100.0000
Totals :				925.18475	208.69975	

Component	Molecular Weight	Absolute Abundance	Relative Abundance
A	7424.18	149282	100.00

*** End of Report ***

ORN-10c

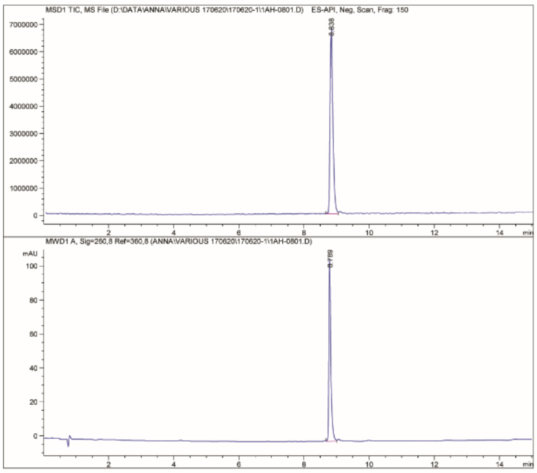

Deconvolution of Spectrum # 1 @ 8.820 - 8.870 min

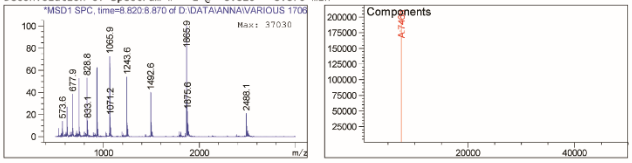

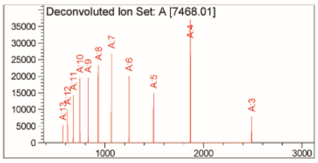

Signal 2: MWD1 A, Sig=260,8 Ref=360,8

Peak #	RetTime [min]	Type	Width [min]	Area [mAU*s]	Height [mAU]	Area %
1	8.789	VV	0.0610	435.61993	108.19009	100.0000
Totals :				435.61993	108.19009	

Component	Molecular Weight	Absolute Abundance	Relative Abundance
A	7468.01	195923	100.00

*** End of Report ***

ORN-10d

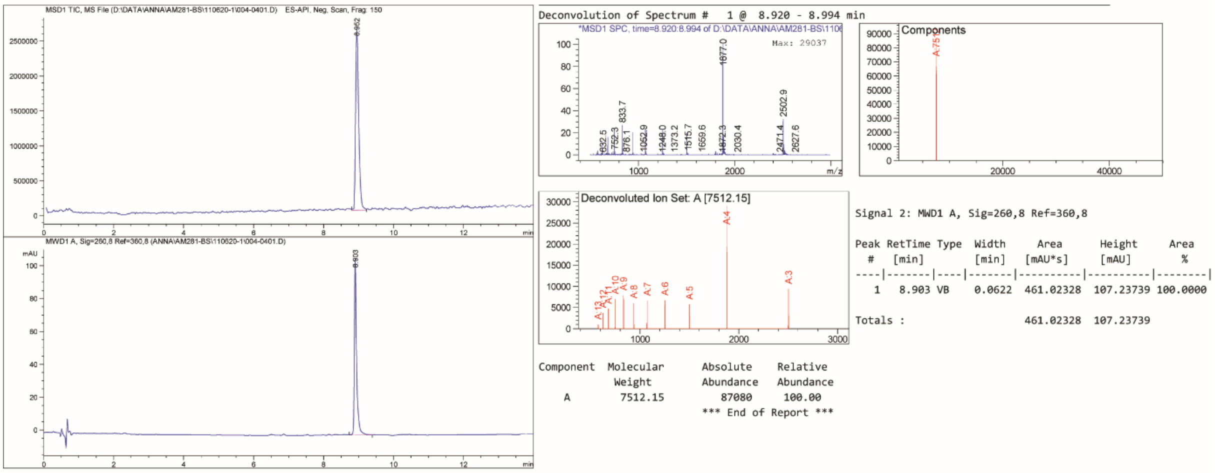

ORN-11

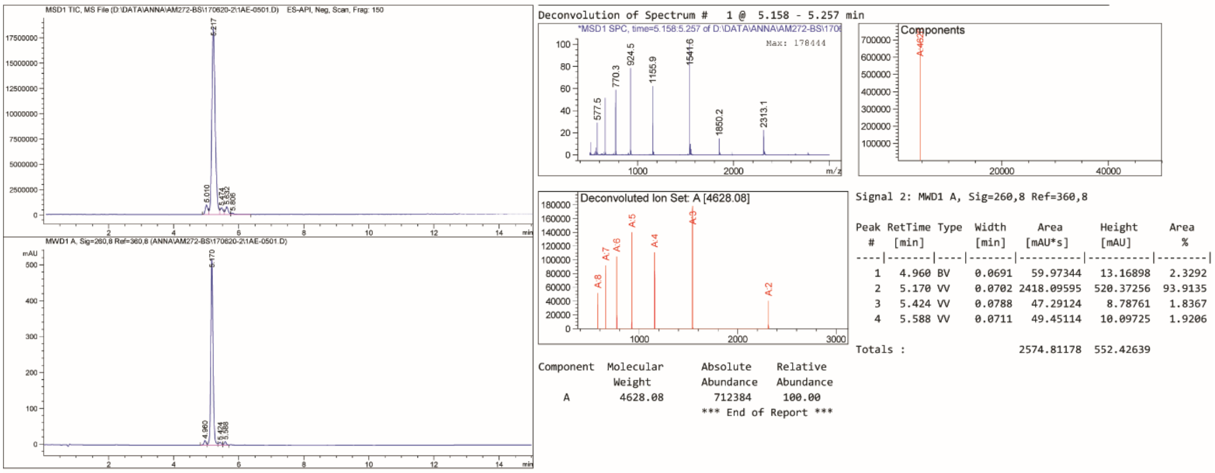

III.2.5. miR-155-3p probes

For this series, small amounts of unidentified side peaks (shoulders) accompanied the product peaks. It was not possible to remove these by double purification and cross-linking experiments were performed with them present in the mixture.

ORN-12a

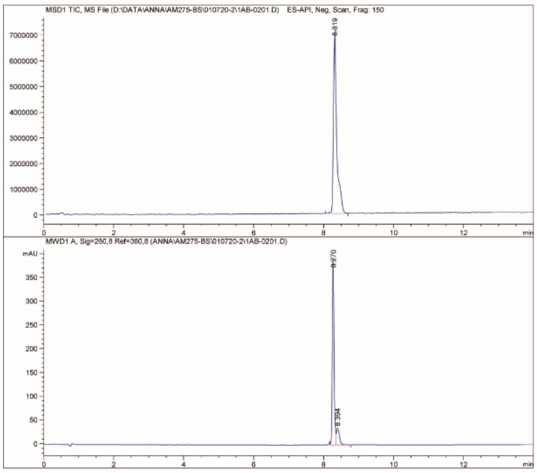

Deconvolution of Spectrum # 1 @ 8.272 - 8.372 min

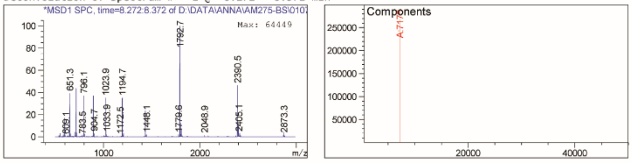

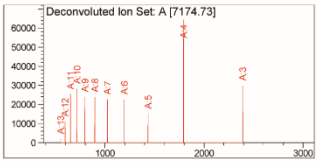

Signal 2: MWD1 A, Sig=260,8 Ref=360,8

Peak #	RetTime [min]	Type	Width [min]	Area [mAU*s]	Height [mAU]	Area %
1	8.270	VV	0.0564	1427.39612	392.65805	86.6213
2	8.394	VB	0.0843	220.46198	35.58944	13.3787
Totals :				1647.85809	428.24749	

Component	Molecular Weight	Absolute Abundance	Relative Abundance
A	7174.73	267943	100.00

*** End of Report ***

ORN-12b

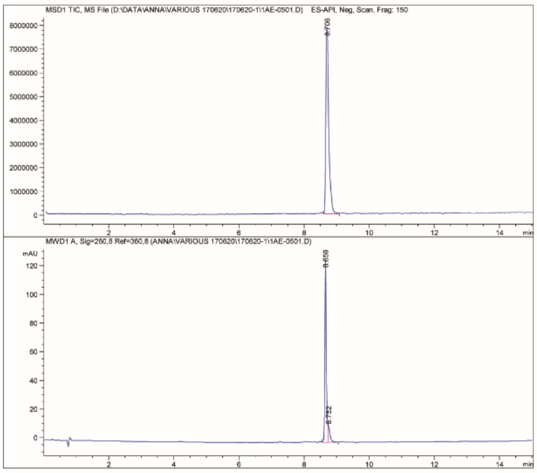

Deconvolution of Spectrum # 1 @ 8.670 - 8.745 min

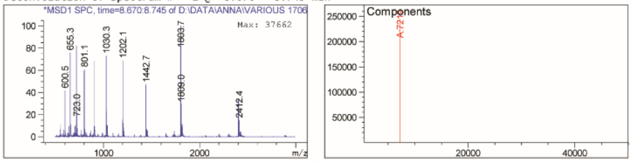

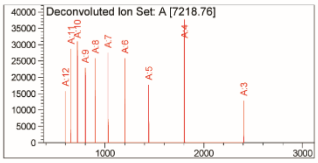

Signal 2: MWD1 A, Sig=260,8 Ref=360,8

Peak #	RetTime [min]	Type	Width [min]	Area [mAU*s]	Height [mAU]	Area %
1	8.659	VV	0.0550	450.85956	128.32999	90.9233
2	8.752	VV	0.0665	45.00866	11.28126	9.0767
Totals :				495.86821	139.61124	

Component	Molecular Weight	Absolute Abundance	Relative Abundance
A	7218.76	243846	100.00

*** End of Report ***

ORN-12c

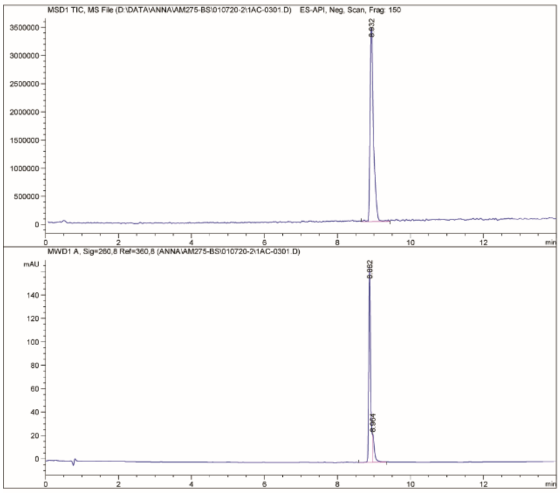

Deconvolution of Spectrum # 1 @ 8.895 - 8.969 min

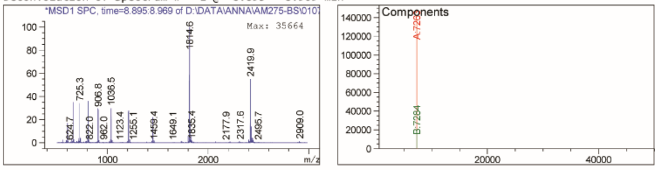

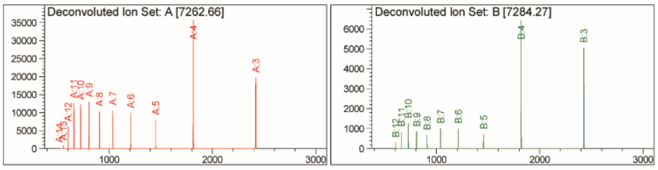

Component	Molecular Weight	Absolute Abundance	Relative Abundance
A	7262.66	137046	100.00
B	7284.27	17251	12.59

*** End of Report ***

Signal 2: MWD1 A, Sig=260,8 Ref=360,8

Peak #	RetTime [min]	Type	Width [min]	Area [mAU*s]	Height [mAU]	Area %
1	8.882	VV	0.0564	602.46820	165.81186	86.3468
2	8.964	VB	0.0666	95.26236	23.82937	13.6532
Totals :				697.73056	189.64123	

ORN-12d

Additional Info : Peak(s) manually integrated

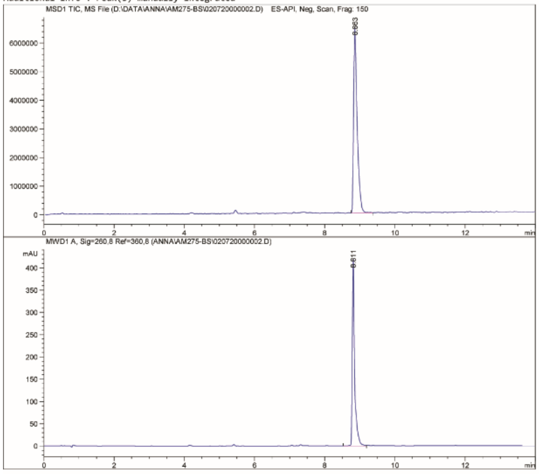

Deconvolution of Spectrum # 1 @ 8.837 - 8.936 min

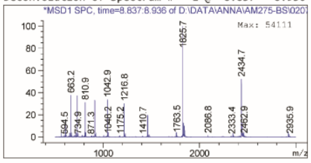

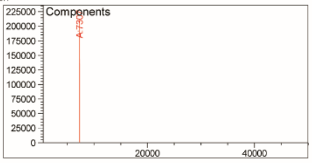

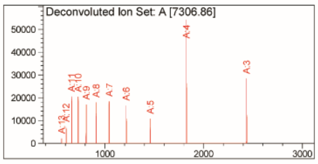

Signal 2: MWD1 A, Sig=260,8 Ref=360,8

Peak #	RetTime [min]	Type	Width [min]	Area [mAU*s]	Height [mAU]	Area %
1	8.811	VV	0.0675	1933.83228	422.04416	100.0000
Totals :				1933.83228	422.04416	

Component	Molecular Weight	Absolute Abundance	Relative Abundance
A	7306.86	211321	100.00

*** End of Report ***

ORN-13

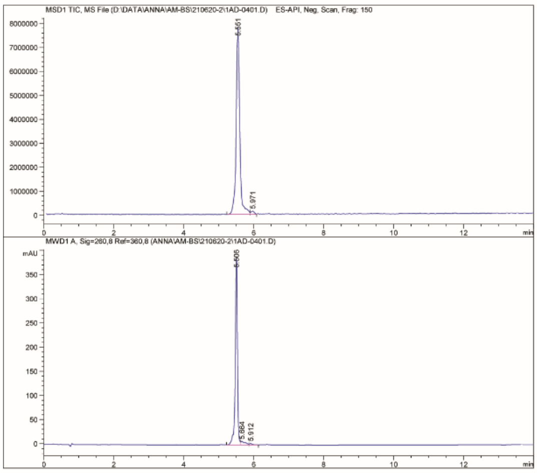

Deconvolution of Spectrum # 1 @ 5.507 - 5.581 min

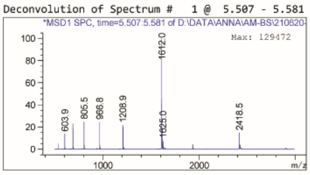

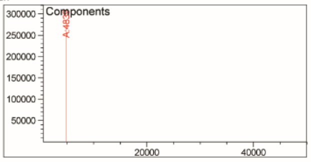

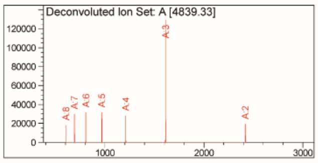

Signal 2: MWD1 A, Sig=260,8 Ref=360,8

Peak #	RetTime [min]	Type	Width [min]	Area [mAU*s]	Height [mAU]	Area %
1	5.506	VV	0.0607	1617.18640	387.15115	94.8858
2	5.664	VV	0.1142	66.13332	7.54742	3.8803
3	5.912	VB	0.0796	21.03070	3.63097	1.2339
Totals :				1704.35042	398.32954	

Component	Molecular Weight	Absolute Abundance	Relative Abundance
A	4839.33	287164	100.00

*** End of Report ***

IV. Chromatograms from the *in vitro* photo-cross-linking experiments

D1

a)

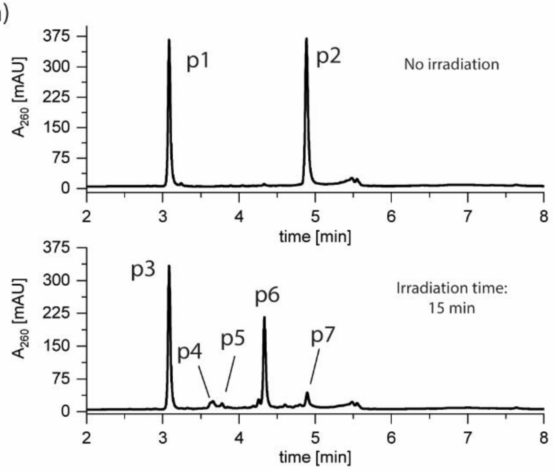

b)

Duplex	ORN	ORN mass	Calc. mass of the CL product
D1	ORN-1	6766.4	11454.3
	ORN-2	4687.9	

c)

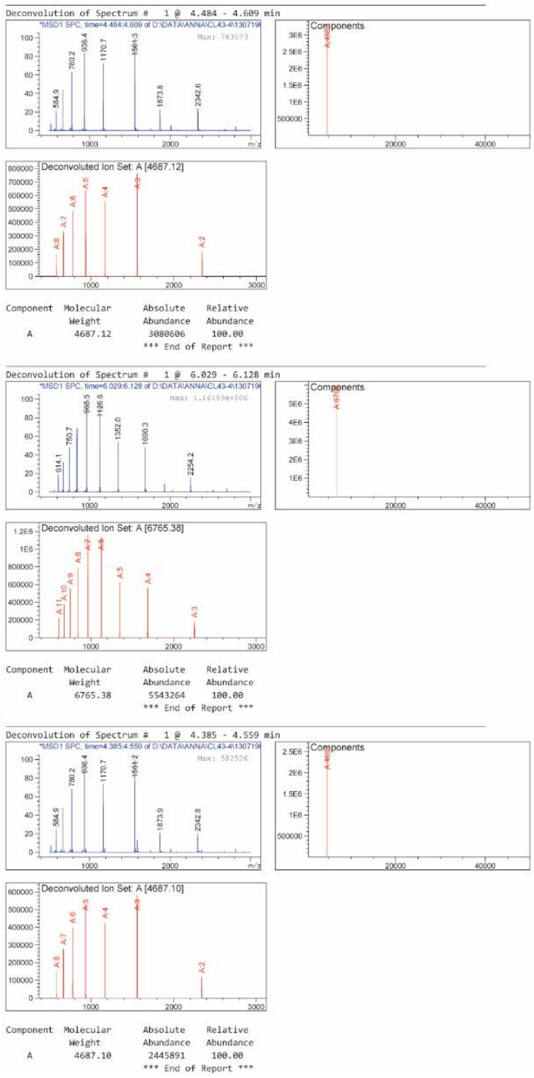

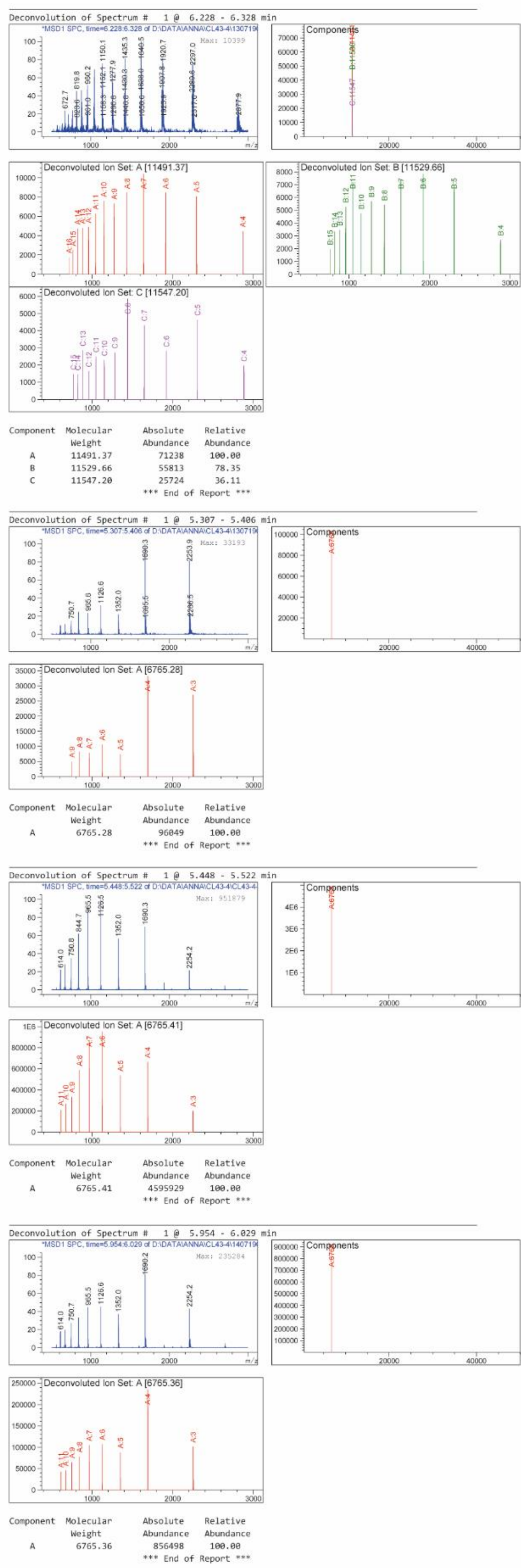

D2

a)

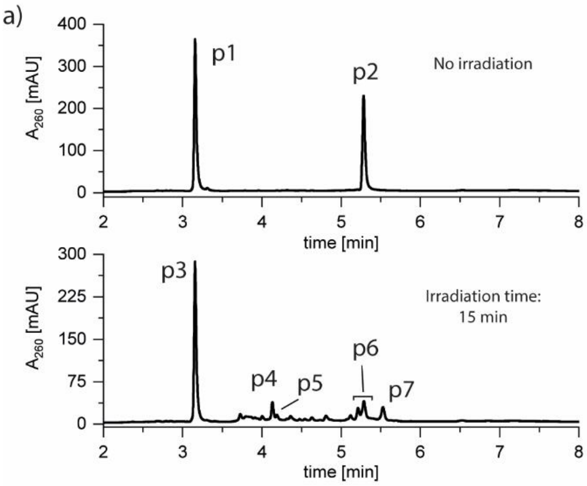

b)

Duplex	ORN	ORN mass	Calc. mass of the CL product
D2	ORN-3	6766.4	11454.3
	ORN-2	4687.9	

c)

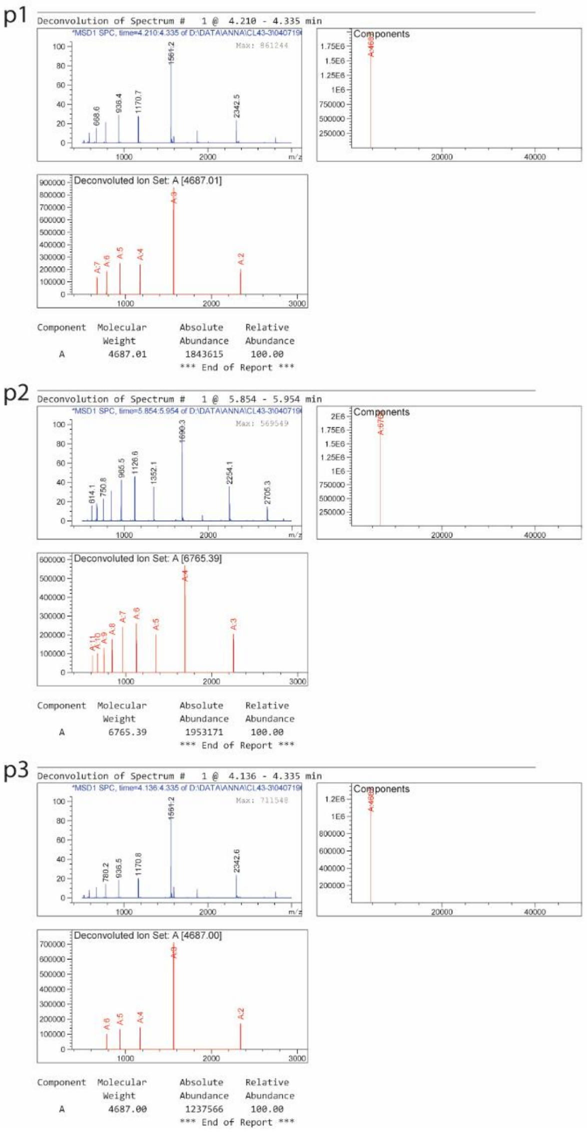

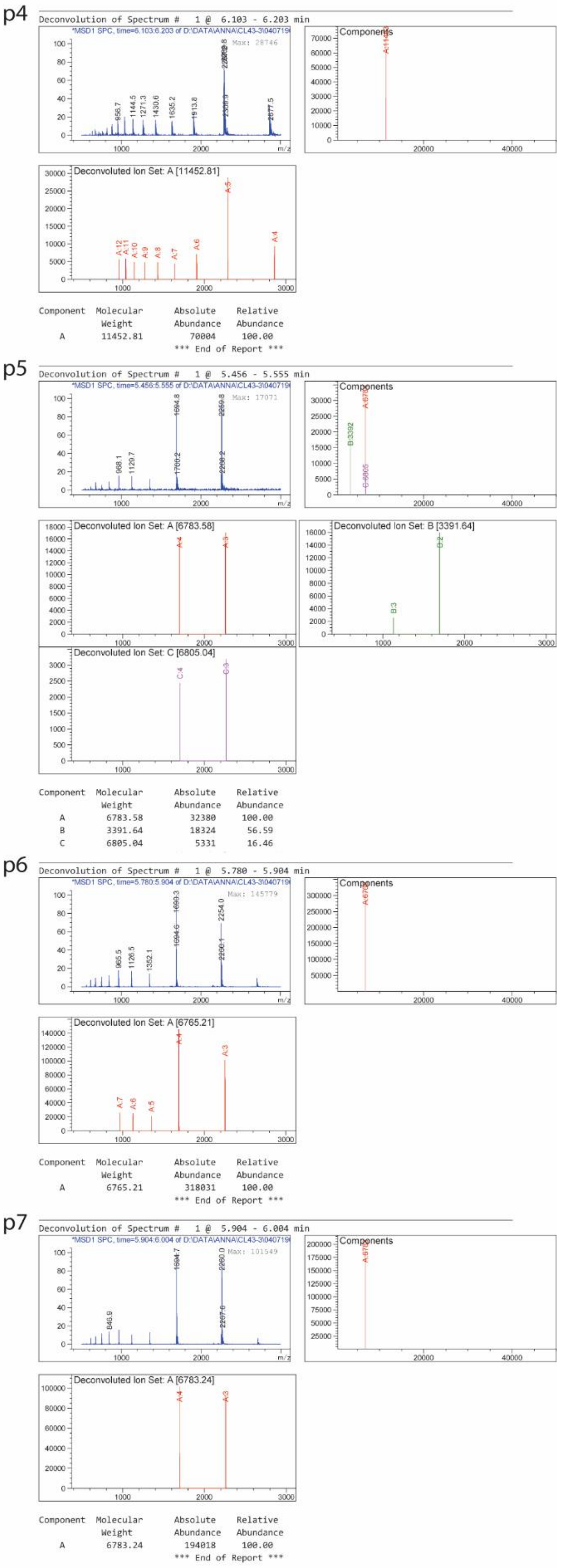

D3

a)

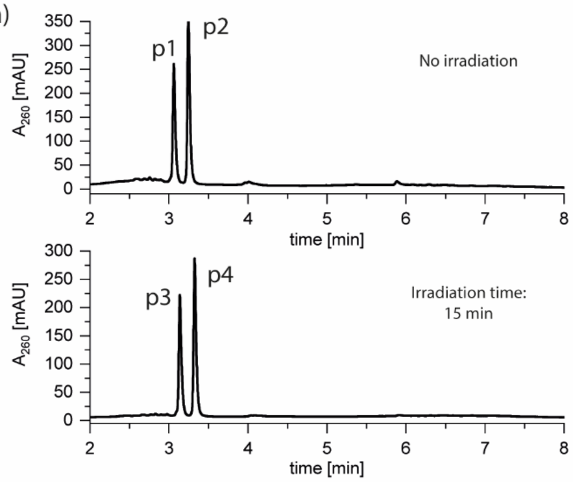

b)

Duplex	ORN	ORN mass	Calc. mass of the CL product
D3	ORN-4	6445.0	11132.9
	ORN-2	4687.9	

c) p1

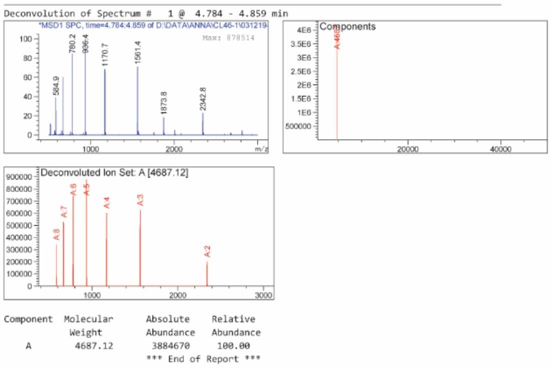

p2

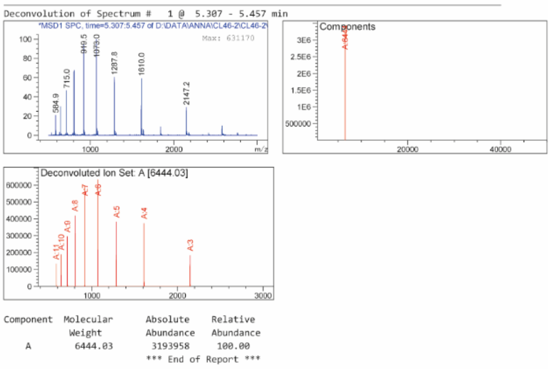

p3

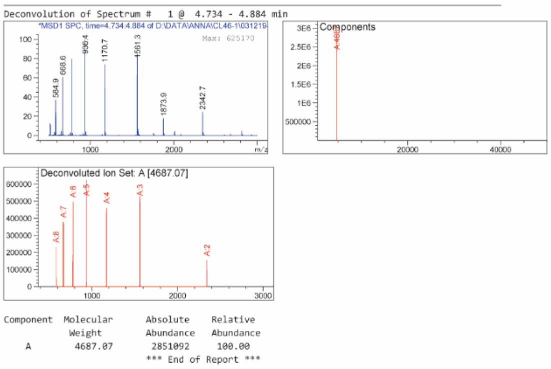

p4

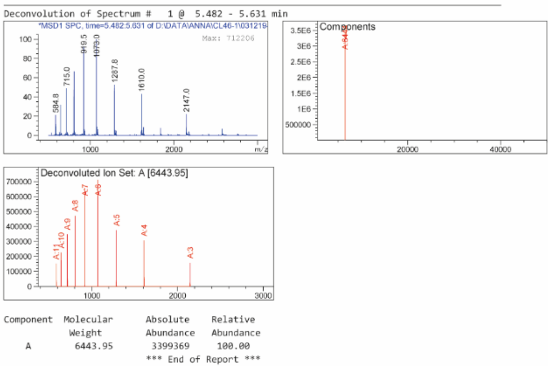

D4

a)

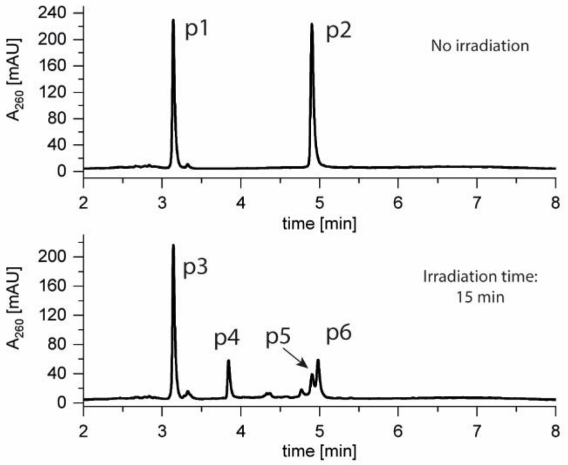

b)

Duplex	ORN	ORN mass	Calc. mass of the CL product
D4	ORN-4a	6685.3	11373.2
	ORN-2	4687.9	

c) p1

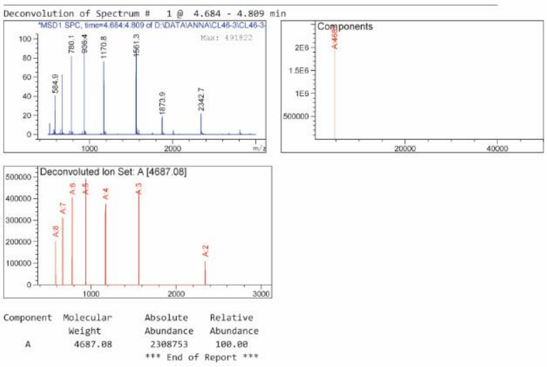

p2

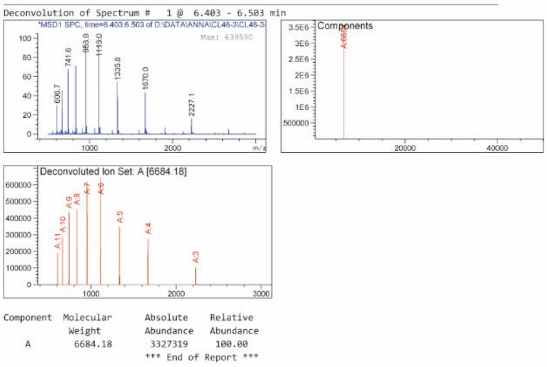

p3

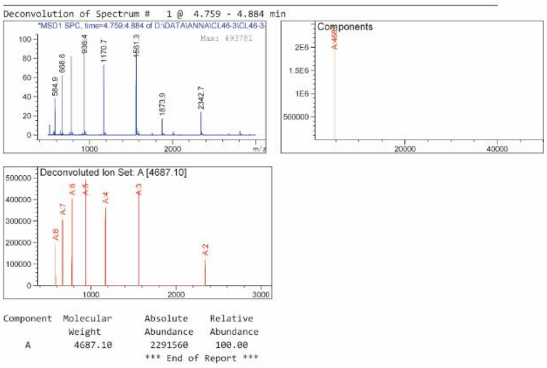

p4

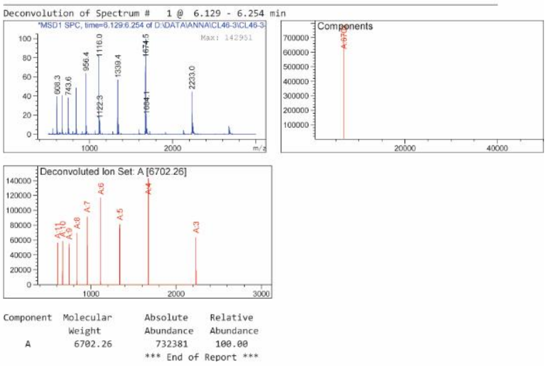

p5

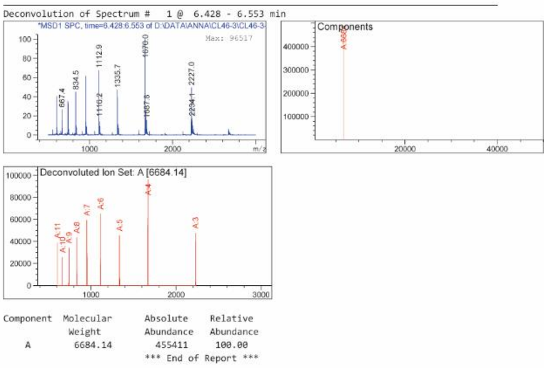

p6

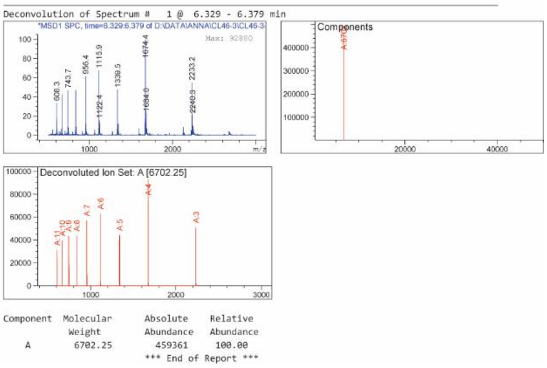

D5

a)

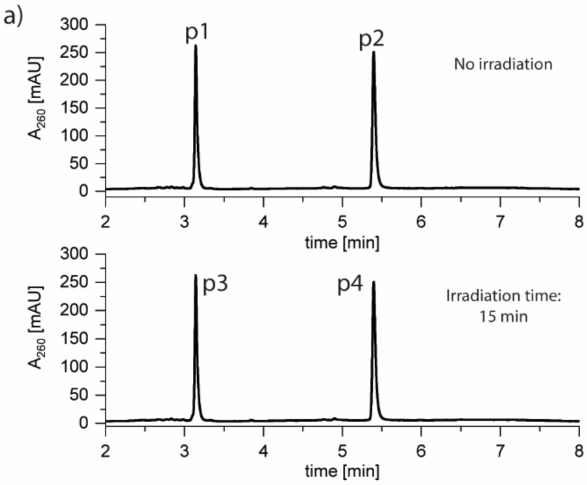

b)

Duplex	ORN	ORN mass	Calc. mass of the CL product
D5	ORN-4b	6729.3	11417.2
	ORN-2	4687.9	

c) p1

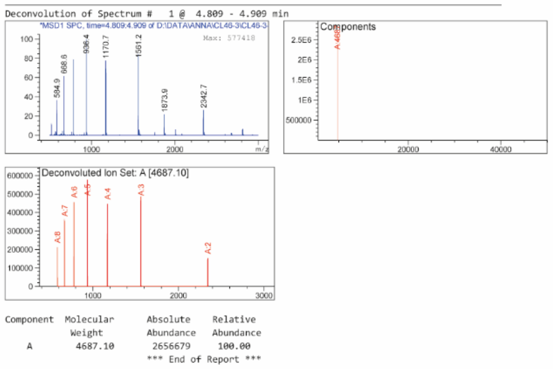

p2 p3 p4

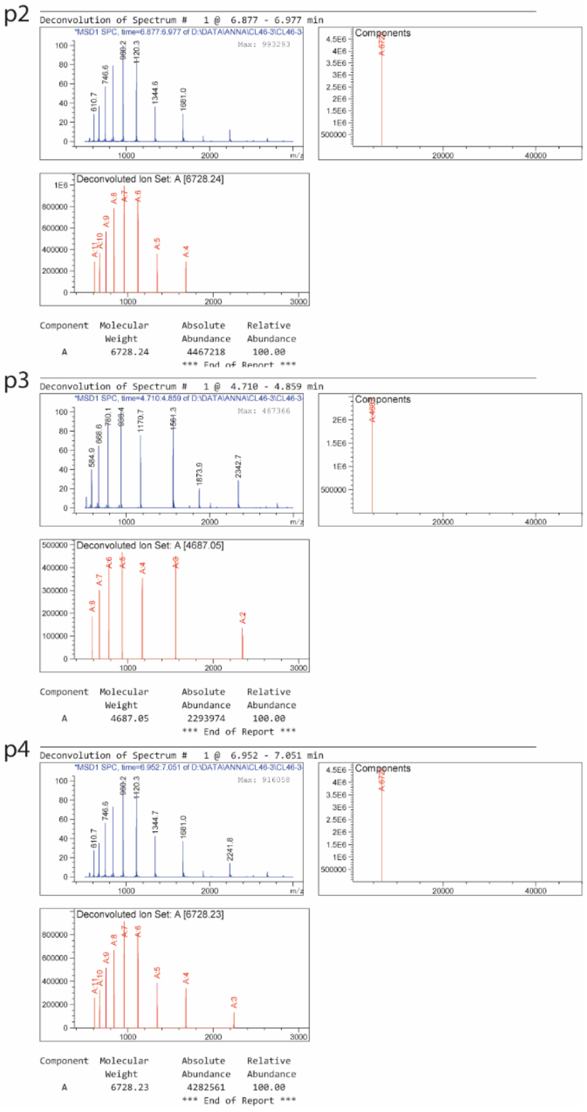

D6

a)

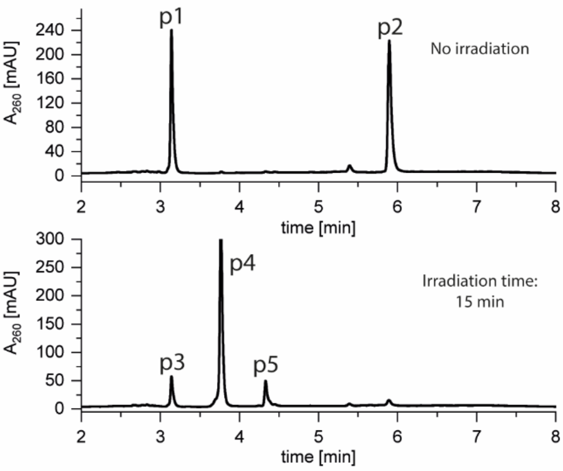

b)

Duplex	ORN	ORN mass	Calc. mass of the CL product
D6	ORN-4c	6773.4	11461.3
	ORN-2	4687.9	

c) p1

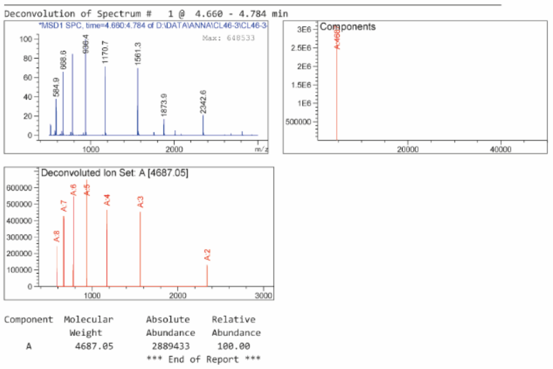

p2

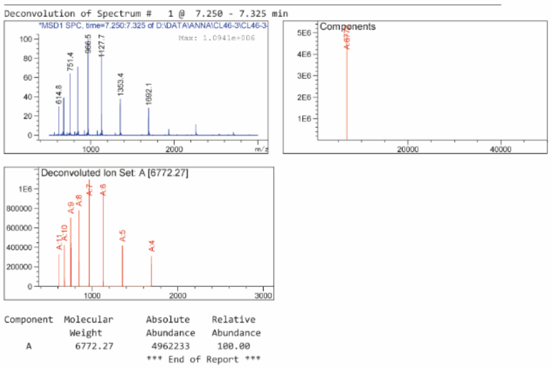

p3

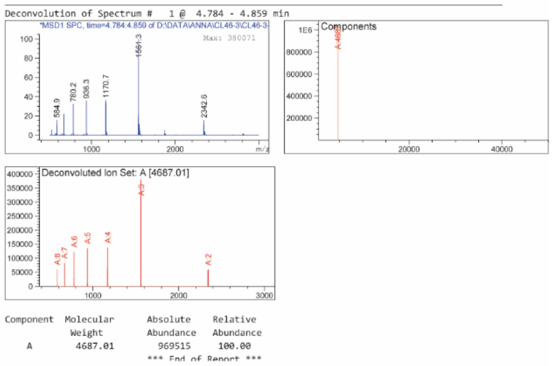

p4

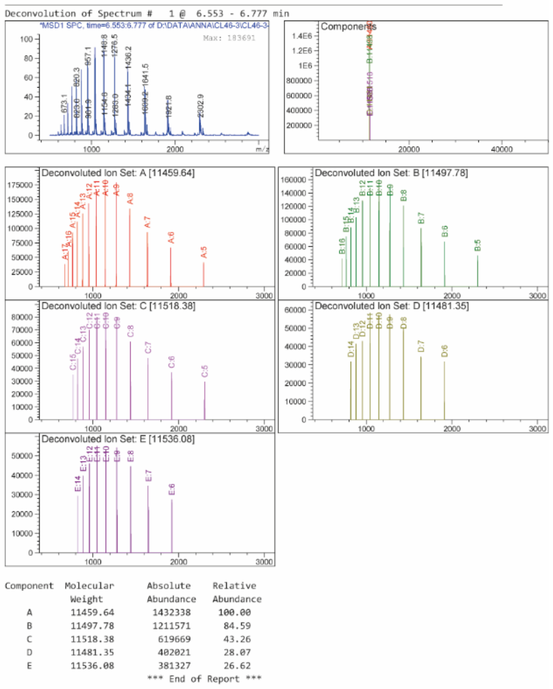

p5

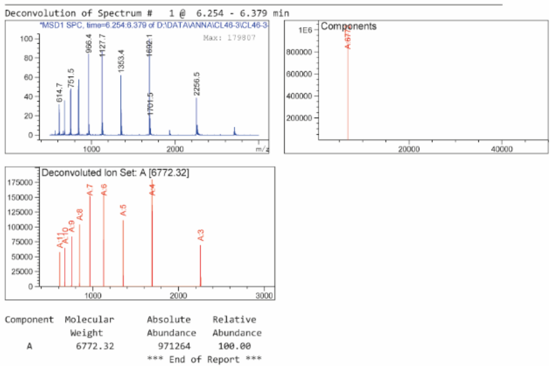

D7

a)

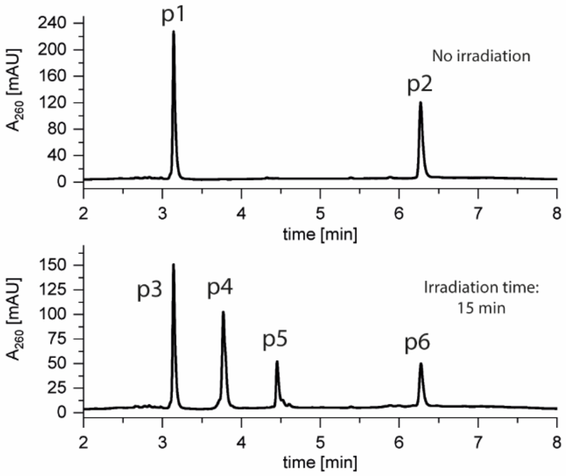

b)

Duplex	ORN	ORN mass	Calc. mass of the CL product
D7	ORN-4d	6817.4	11505.3
	ORN-2	4687.9	

c) p1

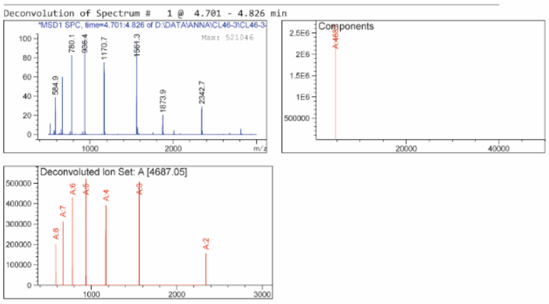

Component	Molecular Weight	Absolute Abundance	Relative Abundance
A	4687.05	2498498	100.00

*** End of Report ***

p2

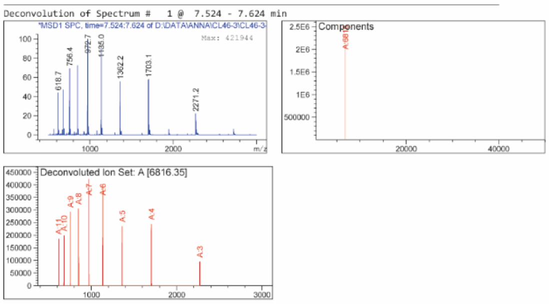

Component	Molecular Weight	Absolute Abundance	Relative Abundance
A	6816.35	2351695	100.00

*** End of Report ***

p3

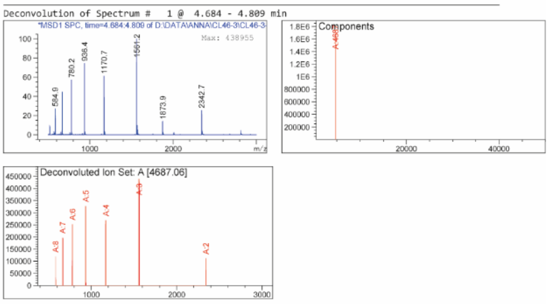

Component	Molecular Weight	Absolute Abundance	Relative Abundance
A	4687.06	1691269	100.00

*** End of Report ***

p4

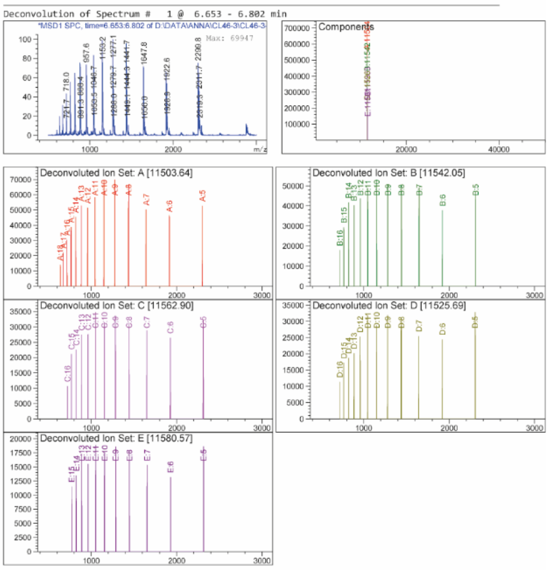

Component	Molecular Weight	Absolute Abundance	Relative Abundance
A	11503.64	664639	100.00
B	11542.05	507106	76.30
C	11562.90	326253	49.09
D	11525.69	296542	44.62
E	11580.57	174708	26.29

*** End of Report ***

p5

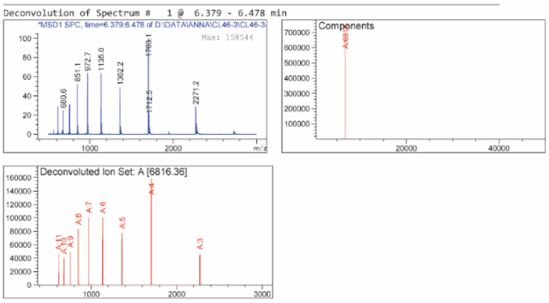

Component	Molecular Weight	Absolute Abundance	Relative Abundance
A	6816.36	697865	100.00

*** End of Report ***

p6

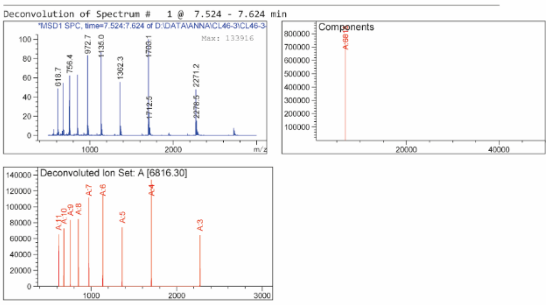

Component	Molecular Weight	Absolute Abundance	Relative Abundance
A	6816.30	799025	100.00

*** End of Report ***

D8

a)

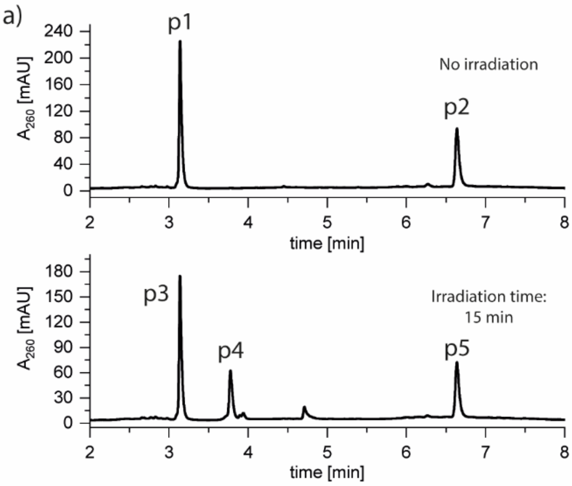

b)

Duplex	ORN	ORN mass	Calc. mass of the CL product
D8	ORN-4e	6861.5	11549.4
	ORN-2	4687.9	

c) p1

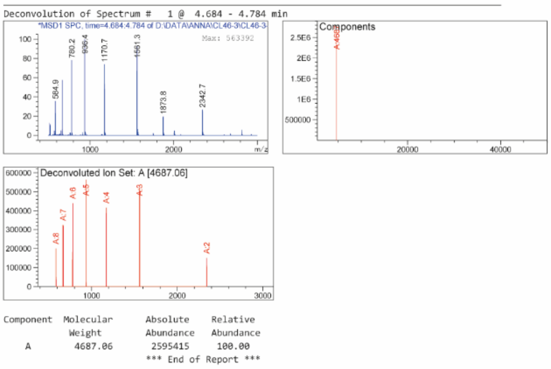

p2

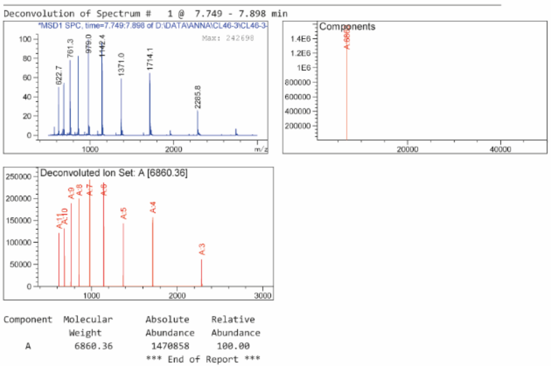

p3

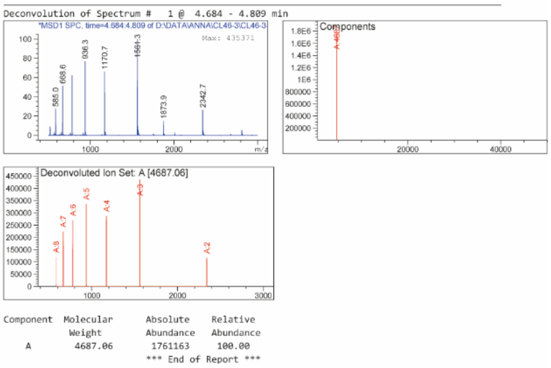

p4

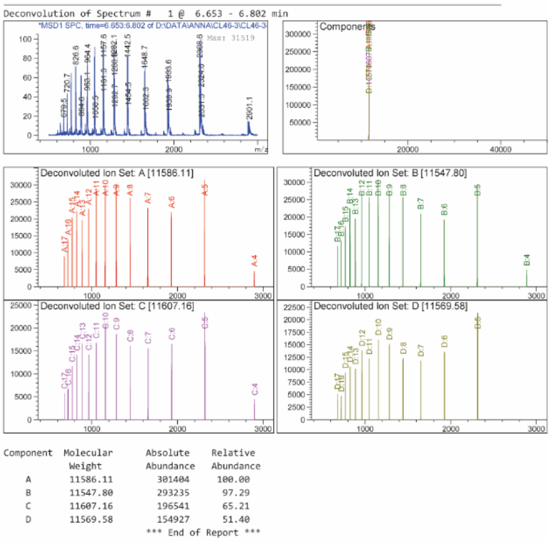

p5

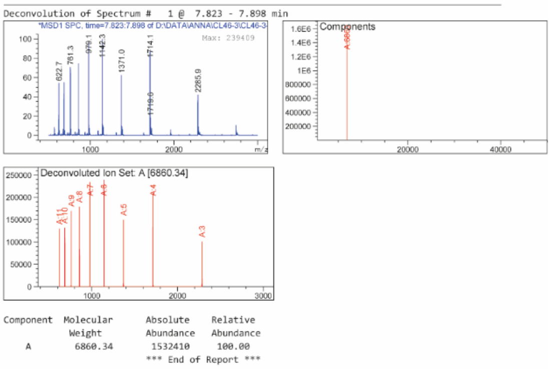

Mixture: ORN-2 and ORN-4a to ORN-4e

a)

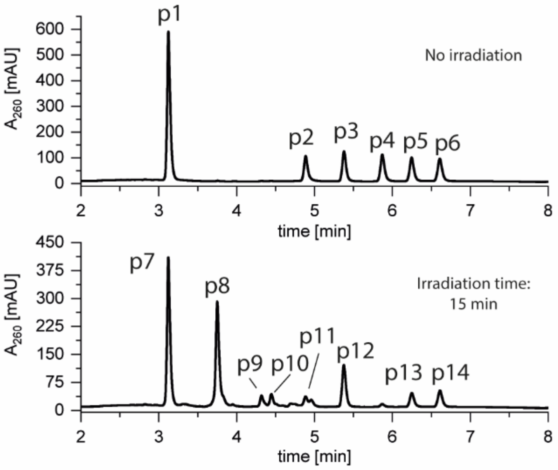

b)

Duplex	ORN	ORN mass	Calc. mass of the CL product
D4	ORN-4a	6685.3	11373.2
	ORN-2	4687.9	
D5	ORN-4b	6729.3	11417.2
	ORN-2	4687.9	
D6	ORN-4c	6773.4	11461.3
	ORN-2	4687.9	
D7	ORN-4d	6817.4	11505.3
	ORN-2	4687.9	
D8	ORN-4e	6861.5	11549.4
	ORN-2	4687.9	

c) p1

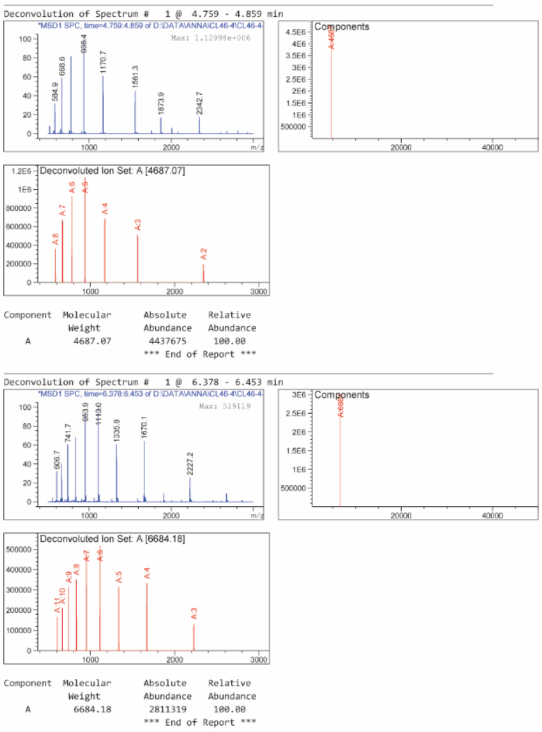

p3

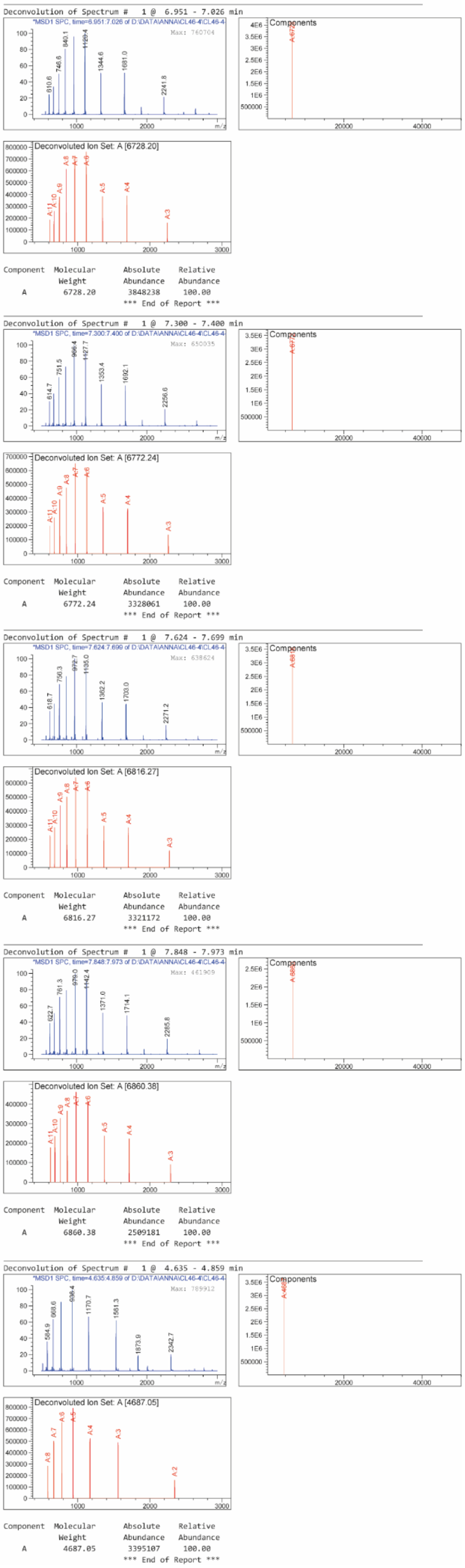

Mixture: ORN-2 and ORN-4a to ORN-4e continued

p8

Deconvolution of Spectrum # 1 @ 6.677 - 6.877 min

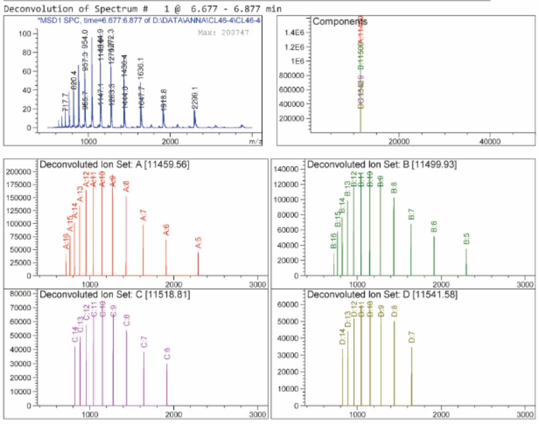

Component	Molecular Weight	Absolute Abundance	Relative Abundance
A	11459.56	1459578	100.00
B	11499.93	1028345	70.45
C	11518.81	475593	32.58
D	11541.58	390454	26.75

*** End of Report ***

p9

Deconvolution of Spectrum # 1 @ 6.204 - 6.329 min

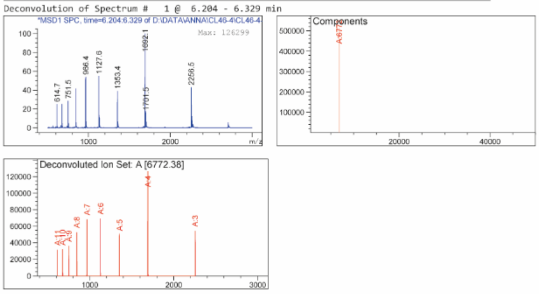

Component	Molecular Weight	Absolute Abundance	Relative Abundance
A	6772.38	513671	100.00

*** End of Report ***

p10

Deconvolution of Spectrum # 1 @ 6.204 - 6.852 min

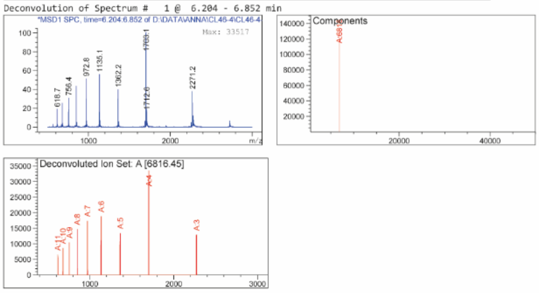

Component	Molecular Weight	Absolute Abundance	Relative Abundance
A	6816.45	134823	100.00

*** End of Report ***

p11

Deconvolution of Spectrum # 1 @ 6.379 - 6.453 min

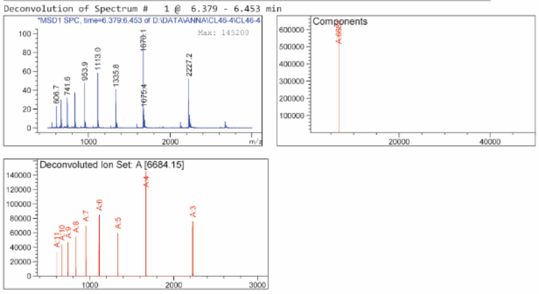

Component	Molecular Weight	Absolute Abundance	Relative Abundance
A	6684.15	608299	100.00

*** End of Report ***

p12

Deconvolution of Spectrum # 1 @ 6.877 - 6.976 min

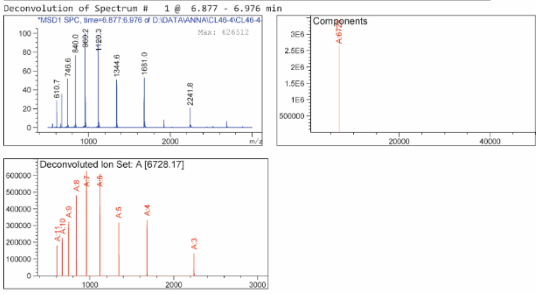

Component	Molecular Weight	Absolute Abundance	Relative Abundance
A	6728.17	3195667	100.00

*** End of Report ***

p13

Deconvolution of Spectrum # 1 @ 7.624 - 7.724 min

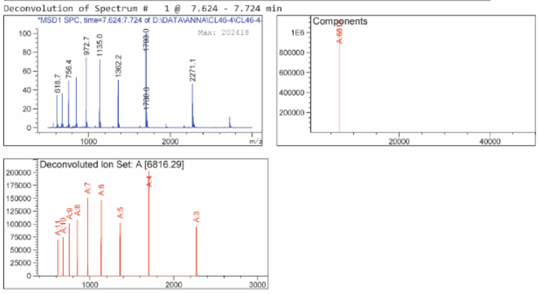

Component	Molecular Weight	Absolute Abundance	Relative Abundance
A	6816.29	1045662	100.00

*** End of Report ***

p14

Deconvolution of Spectrum # 1 @ 7.873 - 7.973 min

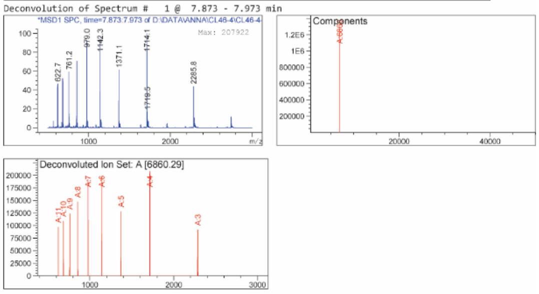

Component	Molecular Weight	Absolute Abundance	Relative Abundance
A	6860.29	1285819	100.00

*** End of Report ***

Mixture: ORN-2 and ORN-5a to ORN-5d

a)

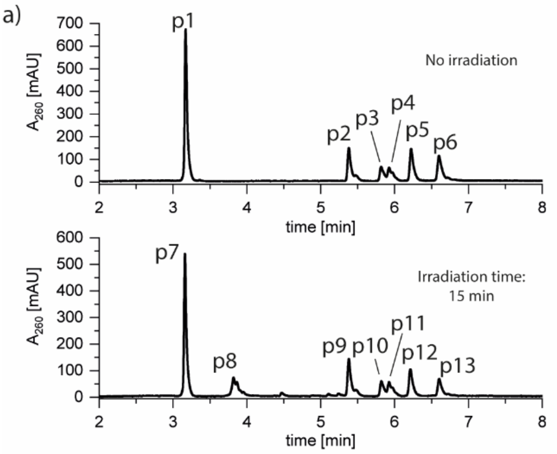

b)

Duplex	ORN	ORN mass	Calc. mass of the CL product
D9	ORN-5a	6729.3	11417.2
	ORN-2	4687.9	
D10	ORN-5b	6773.4	11461.3
	ORN-2	4687.9	
D11	ORN-5c	6817.4	11505.3
	ORN-2	4687.9	
D12	ORN-5d	6861.5	11549.4
	ORN-2	4687.9	

c)

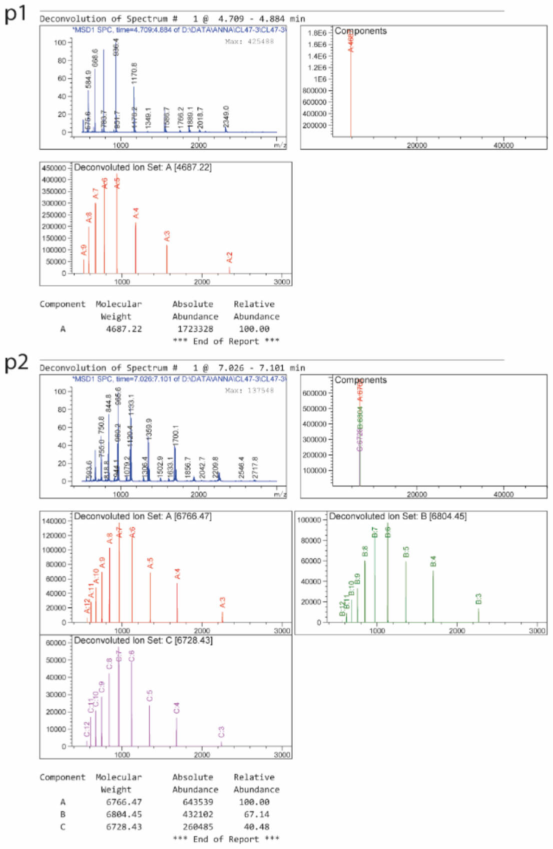

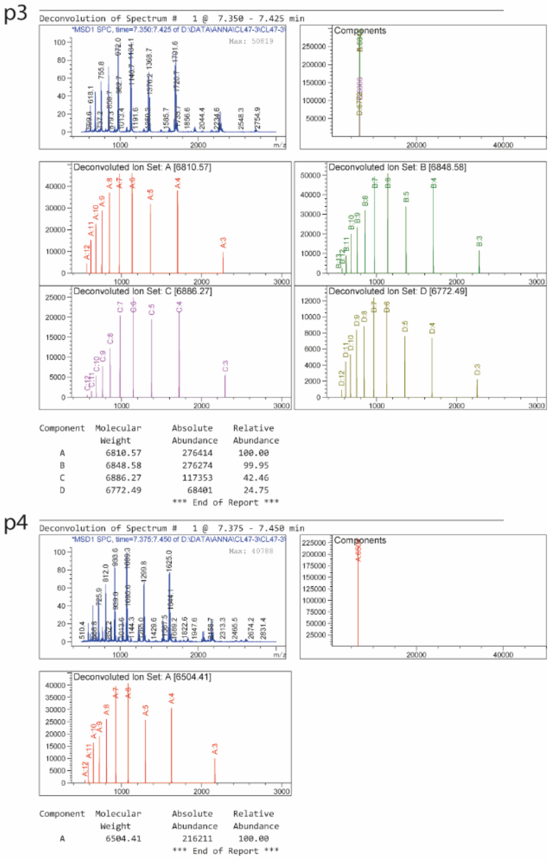

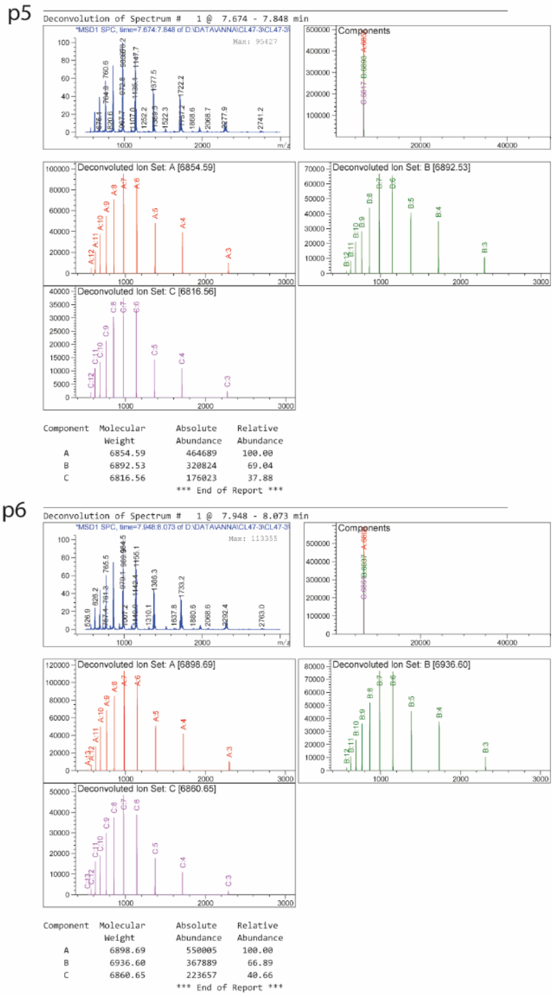

Mixture: ORN-2 and ORN-5a to ORN-5d continued

p7

Deconvolution of Spectrum # 1 @ 4.709 - 4.809 min

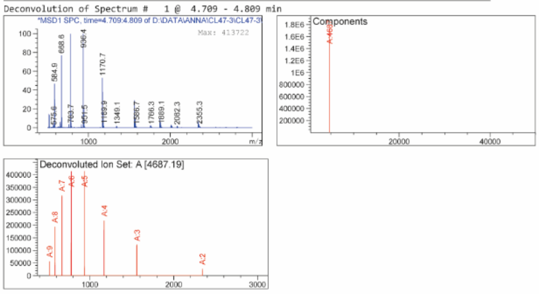

Component	Molecular Weight	Absolute Abundance	Relative Abundance
A	4687.19	1740489	100.00

*** End of Report ***

p8

Deconvolution of Spectrum # 1 @ 6.453 - 6.777 min

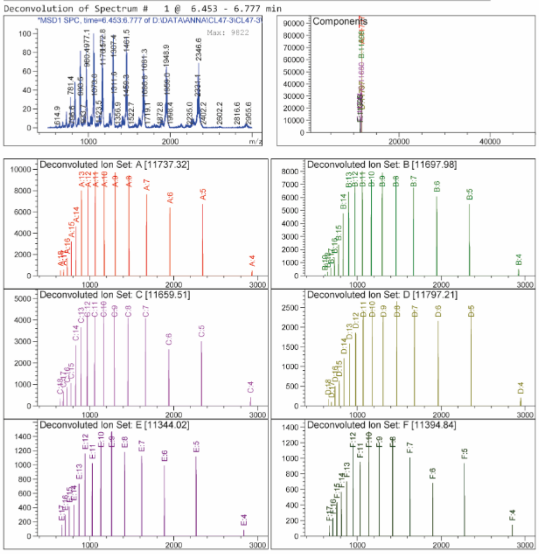

Component	Molecular Weight	Absolute Abundance	Relative Abundance
A	11737.32	86051	100.00
B	11697.98	71609	83.22
C	11659.51	40473	47.03
D	11797.21	22103	25.69
E	11344.02	11145	12.95
F	11394.84	9707	11.28

*** End of Report ***

p9

Deconvolution of Spectrum # 1 @ 7.027 - 7.126 min

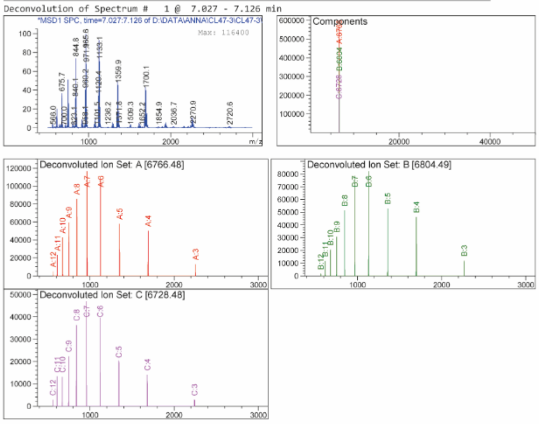

Component	Molecular Weight	Absolute Abundance	Relative Abundance
A	6766.48	558324	100.00
B	6804.49	386207	69.17
C	6728.48	209663	37.55

*** End of Report ***

p10

Deconvolution of Spectrum # 1 @ 7.400 - 7.549 min

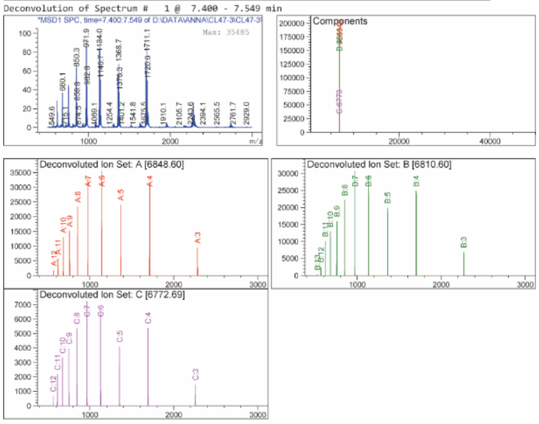

Component	Molecular Weight	Absolute Abundance	Relative Abundance
A	6848.60	191611	100.00
B	6810.60	174238	90.93
C	6772.69	39636	20.69

*** End of Report ***

p11

Deconvolution of Spectrum # 1 @ 7.325 - 7.450 min

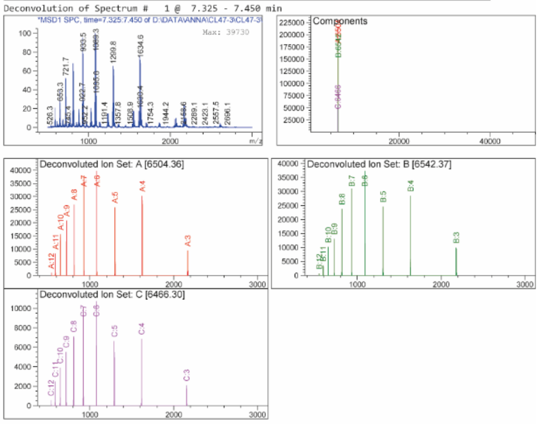

Component	Molecular Weight	Absolute Abundance	Relative Abundance
A	6504.36	214174	100.00
B	6542.37	179879	83.99
C	6466.30	55445	25.89

*** End of Report ***

p12

Deconvolution of Spectrum # 1 @ 7.674 - 7.799 min

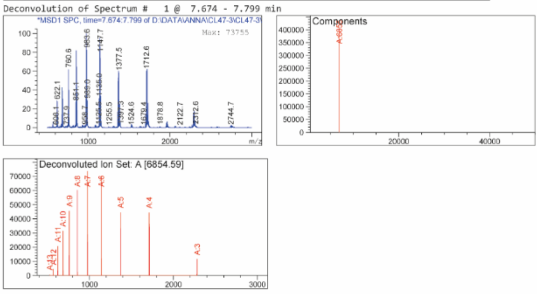

Component	Molecular Weight	Absolute Abundance	Relative Abundance
A	6854.59	406514	100.00

*** End of Report ***

p13

Deconvolution of Spectrum # 1 @ 7.973 - 8.073 min

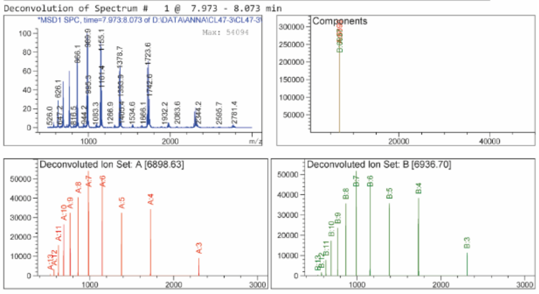

Component	Molecular Weight	Absolute Abundance	Relative Abundance
A	6898.63	297380	100.00
B	6936.70	266098	89.48

*** End of Report ***

Mixture: ORN-6a to ORN-6d and ORN-7

a)

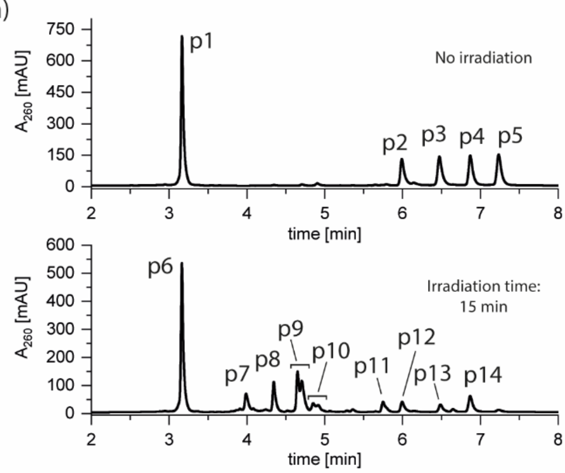

b)

Duplex	ORN	ORN mass	Calc. mass of the CL product
D13	ORN-6a	7592.7	12384.6
	ORN-7	4791.9	
D14	ORN-6b	7636.8	12428.7
	ORN-7	4791.9	
D15	ORN-6c	7680.8	12472.7
	ORN-7	4791.9	
D16	ORN-6d	7724.9	12516.8
	ORN-7	4791.9	

c)

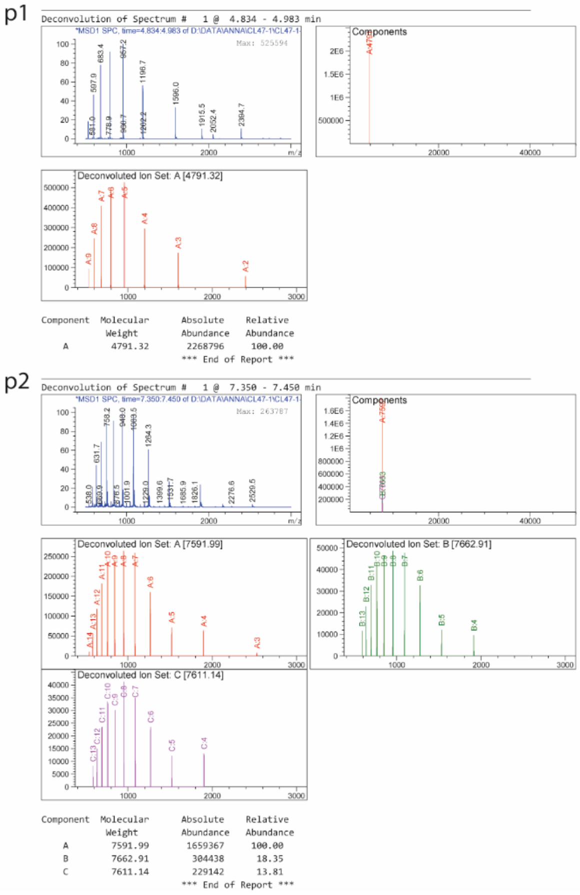

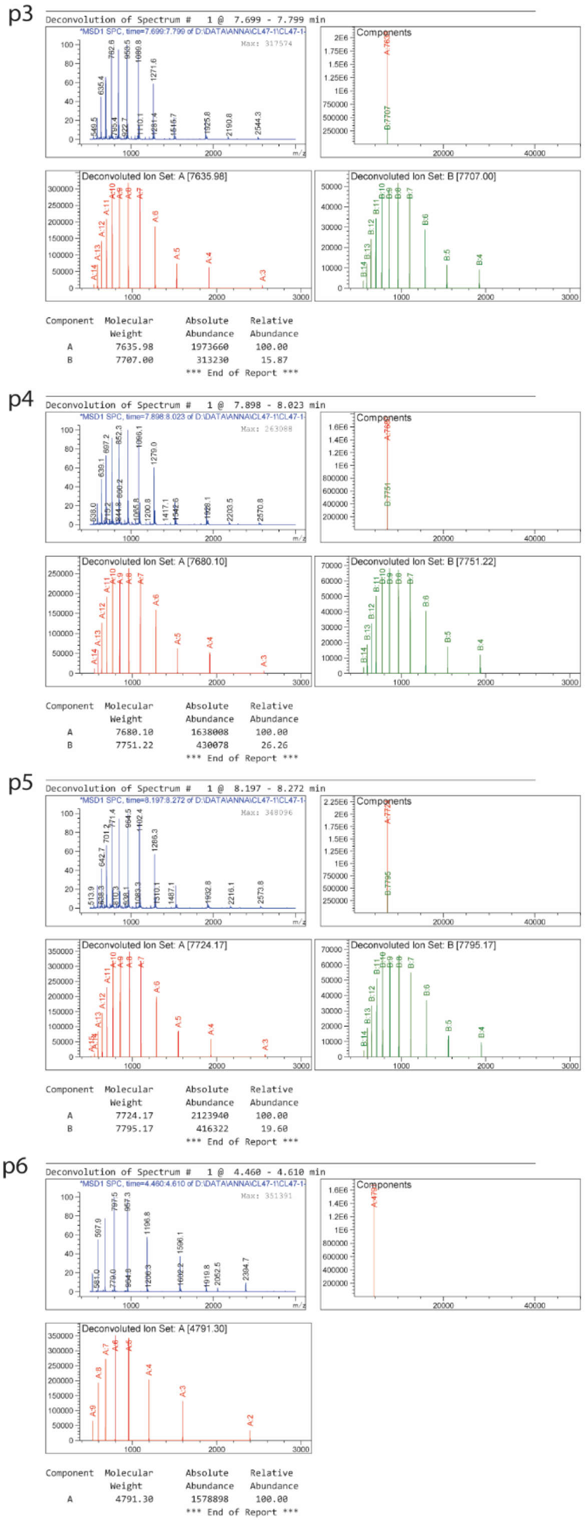

Mixture: ORN-6a to ORN-6d and ORN-7 continued

p7

Deconvolution of Spectrum # 1 @ 6.653 - 6.852 min

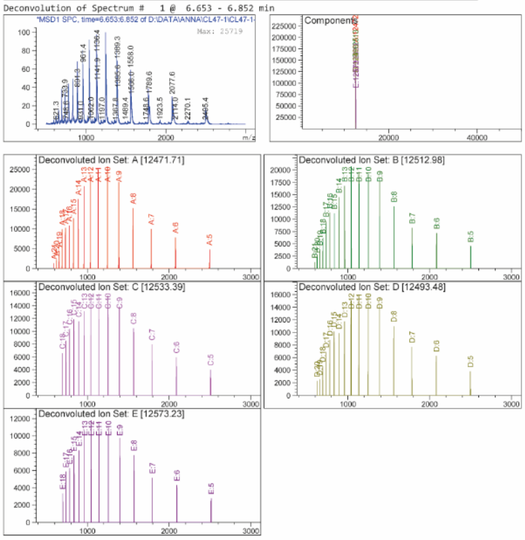

Component	Molecular Weight	Absolute Abundance	Relative Abundance
A	12471.71	222633	100.00
B	12512.98	184494	82.87
C	12533.39	148541	66.72
D	12493.48	145141	65.19
E	12573.23	104753	47.05

*** End of Report ***

p8

Deconvolution of Spectrum # 1 @ 6.702 - 6.827 min

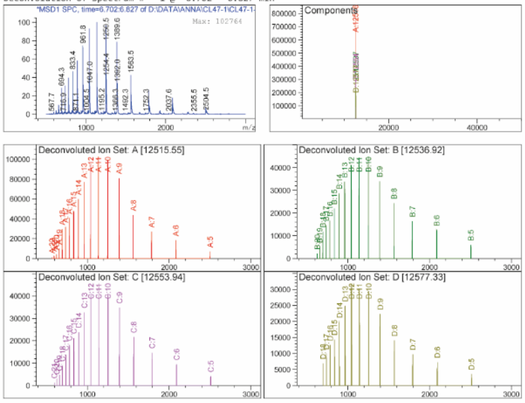

Component	Molecular Weight	Absolute Abundance	Relative Abundance
A	12515.55	774034	100.00
B	12536.92	356283	46.03
C	12553.94	336416	43.46
D	12577.33	233621	30.18

*** End of Report ***

p9

Deconvolution of Spectrum # 1 @ 6.453 - 6.603 min

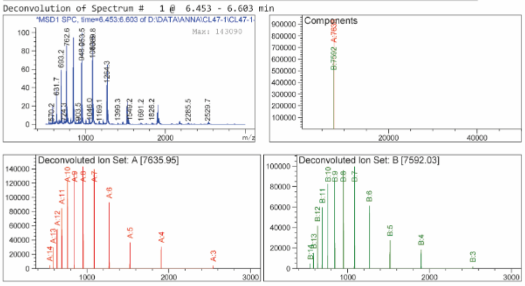

Component	Molecular Weight	Absolute Abundance	Relative Abundance
A	7635.95	874674	100.00
B	7592.03	595133	68.04

*** End of Report ***

p10

Deconvolution of Spectrum # 1 @ 6.179 - 6.304 min

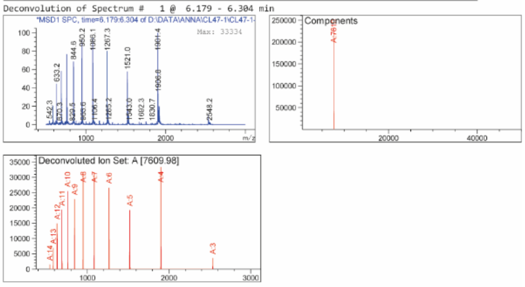

Component	Molecular Weight	Absolute Abundance	Relative Abundance
A	7609.98	233532	100.00

*** End of Report ***

p11

Deconvolution of Spectrum # 1 @ 7.226 - 7.325 min

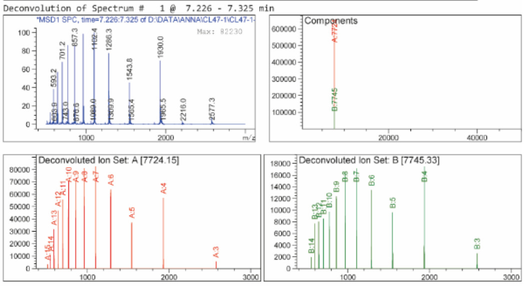

Component	Molecular Weight	Absolute Abundance	Relative Abundance
A	7724.15	609975	100.00
B	7745.33	123620	20.27

*** End of Report ***

p12

Deconvolution of Spectrum # 1 @ 7.226 - 7.375 min

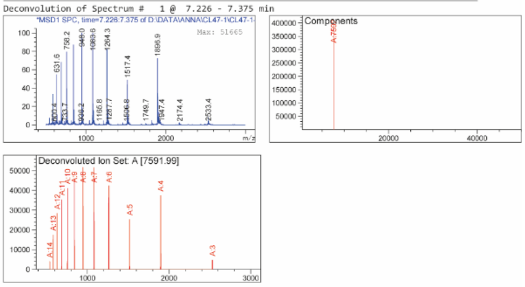

Component	Molecular Weight	Absolute Abundance	Relative Abundance
A	7591.99	382596	100.00

*** End of Report ***

p13

Deconvolution of Spectrum # 1 @ 7.599 - 7.699 min

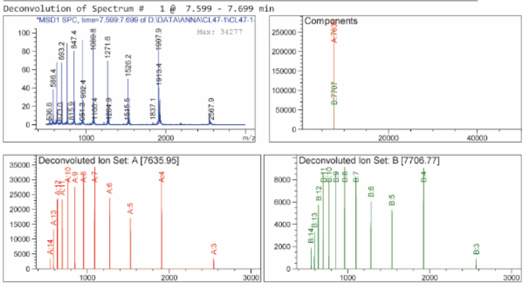

Component	Molecular Weight	Absolute Abundance	Relative Abundance
A	7635.95	261849	100.00
B	7706.77	73899	28.22

*** End of Report ***

p14

Deconvolution of Spectrum # 1 @ 7.873 - 7.973 min

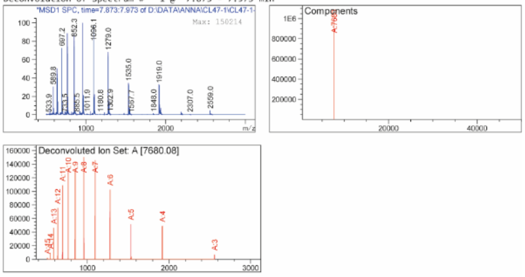

Component	Molecular Weight	Absolute Abundance	Relative Abundance
A	7680.08	1009890	100.00

*** End of Report ***

Mixture: ORN-8a to ORN-8d and ORN-9

a)

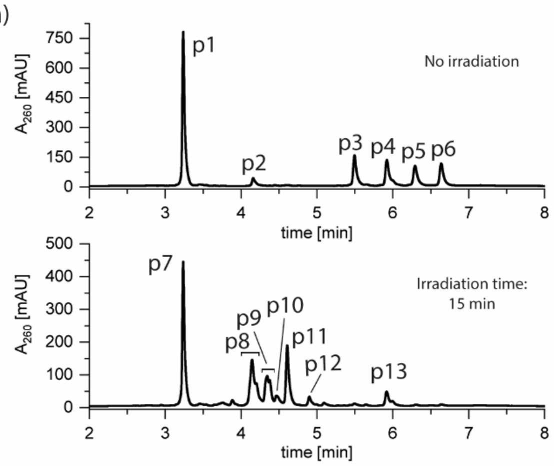

b)

Duplex	ORN	ORN mass	Calc. mass of the CL product
D17	ORN-8a	7327.6	12064.5
	ORN-9	4736.9	
D18	ORN-8b	7371.7	12108.6
	ORN-9	4736.9	
D19	ORN-8c	7415.7	12152.6
	ORN-9	4736.9	
D20	ORN-8d	7459.8	12196.7
	ORN-9	4736.9	

c) p1

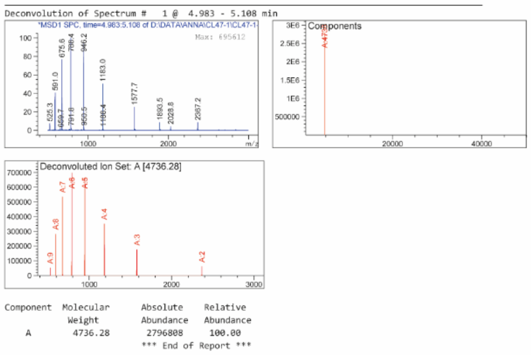

p2

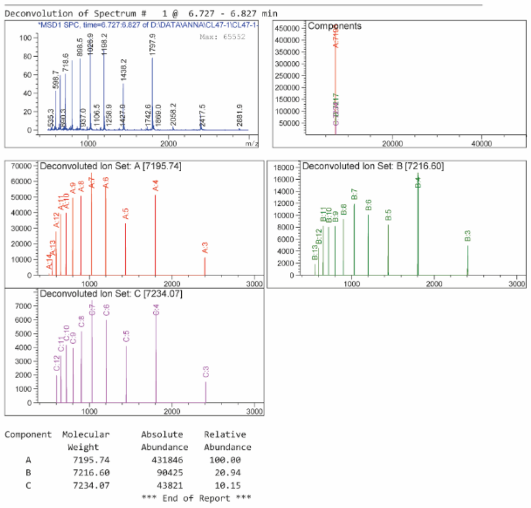

p3

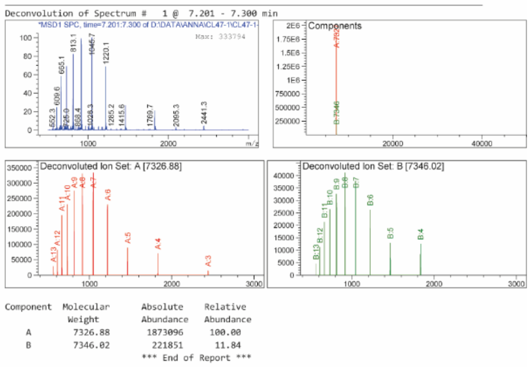

p4

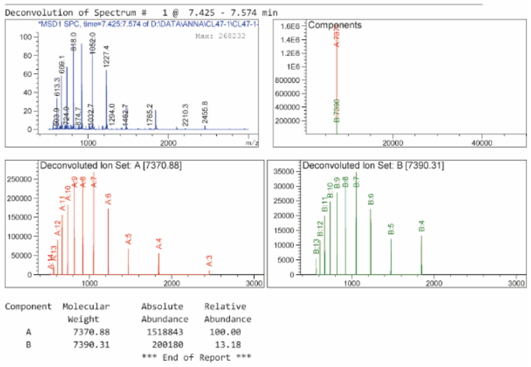

p5

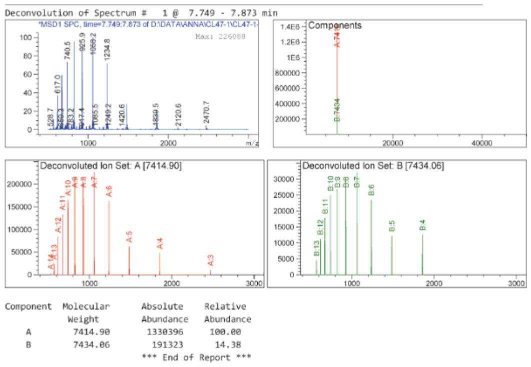

p6

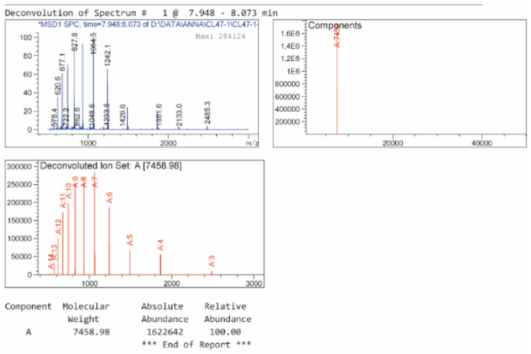

p7

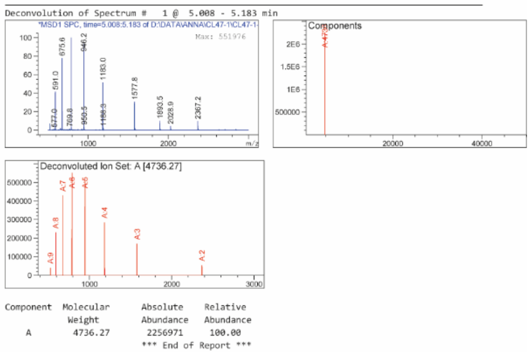

Mixture: ORN-8a to ORN-8d and ORN-9 continued

p8

Deconvolution of Spectrum # 1 @ 6.777 - 6.927 min

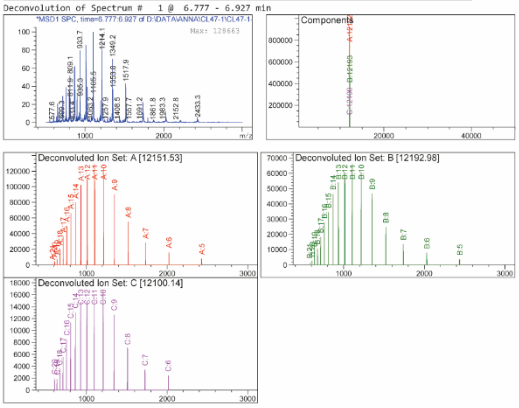

Component	Molecular Weight	Absolute Abundance	Relative Abundance
A	12151.53	924379	100.00
B	12192.98	482437	52.19
C	12100.14	131637	14.24

*** End of Report ***

p9

Deconvolution of Spectrum # 1 @ 6.677 - 6.827 min

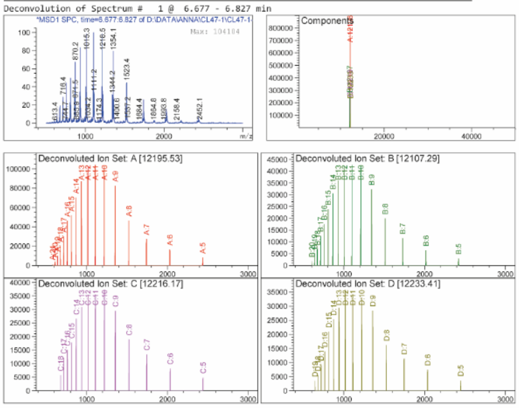

Component	Molecular Weight	Absolute Abundance	Relative Abundance
A	12195.53	806359	100.00
B	12107.29	331364	41.09
C	12216.17	288584	35.79
D	12233.41	274042	33.99

*** End of Report ***

p10

Deconvolution of Spectrum # 1 @ 6.727 - 7.026 min

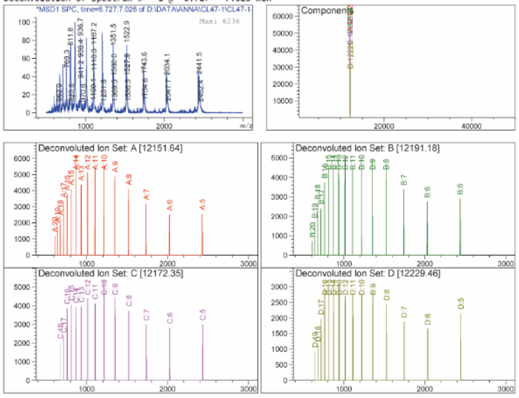

Component	Molecular Weight	Absolute Abundance	Relative Abundance
A	12151.64	59574	100.00
B	12191.18	58181	97.66
C	12172.35	50569	84.88
D	12229.46	34075	57.20

*** End of Report ***

p11

Deconvolution of Spectrum # 1 @ 6.827 - 6.902 min

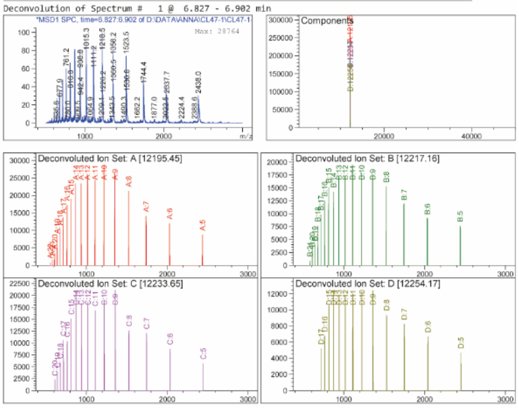

Component	Molecular Weight	Absolute Abundance	Relative Abundance
A	12195.45	279532	100.00
B	12217.16	196121	70.16
C	12233.65	193617	69.26
D	12254.17	117253	41.95

*** End of Report ***

p12

Deconvolution of Spectrum # 1 @ 6.653 - 6.752 min

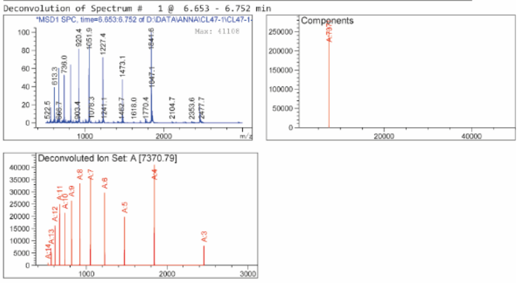

Component	Molecular Weight	Absolute Abundance	Relative Abundance
A	7370.79	262225	100.00

*** End of Report ***

p13

Deconvolution of Spectrum # 1 @ 7.475 - 7.574 min

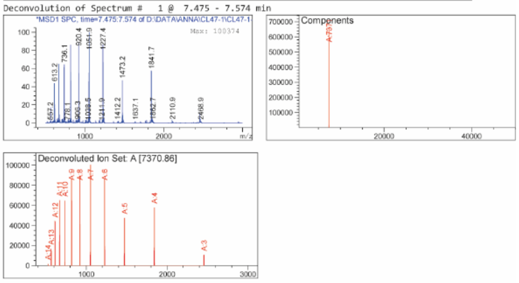

Component	Molecular Weight	Absolute Abundance	Relative Abundance
A	7370.86	665743	100.00

*** End of Report ***

Mixture: ORN-10a to ORN-10d and ORN-11

a)

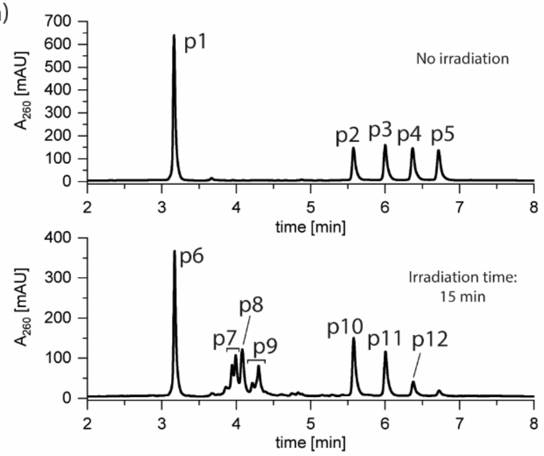

b)

Duplex	ORN	ORN mass	Calc. mass of the CL product
D21	ORN-10a	7380.7	12009.4
	ORN-11	4628.7	
D22	ORN-10b	7424.8	12053.5
	ORN-11	4628.7	
D23	ORN-10c	7468.8	12097.5
	ORN-11	4628.7	
D24	ORN-10d	7512.9	12141.6
	ORN-11	4628.7	

c)

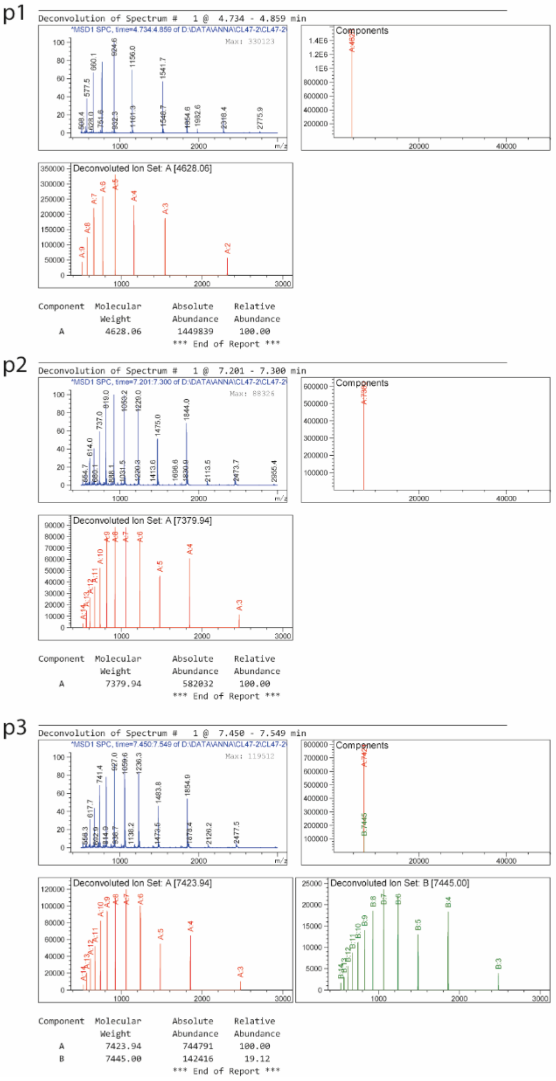

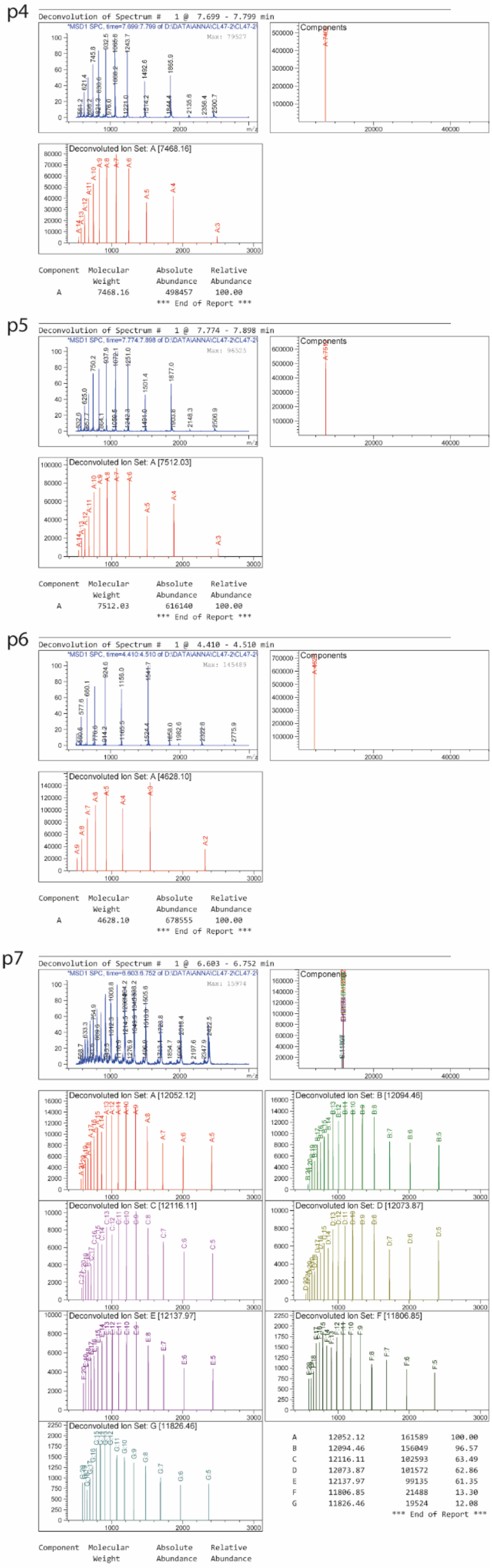

Mixture: ORN-10a to ORN-10d and ORN-11 continued

p8

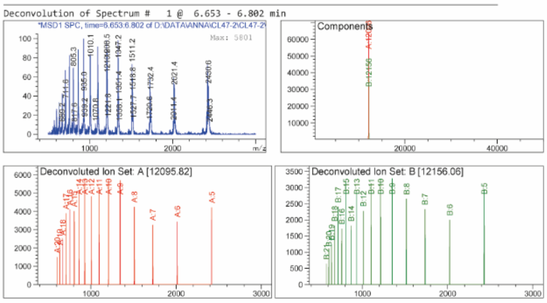

Component	Molecular Weight	Absolute Abundance	Relative Abundance
A	12095.82	63697	100.00
B	12156.06	37111	58.26

*** End of Report ***

p9

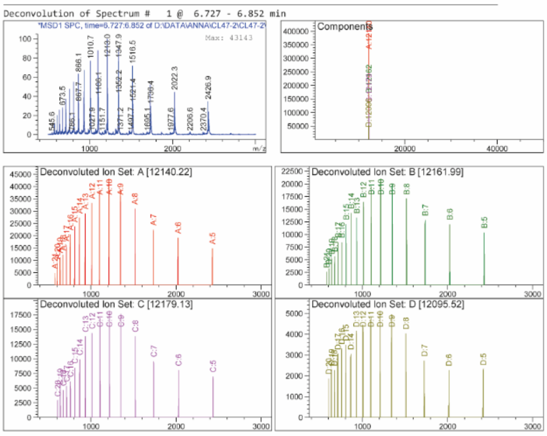

Component	Molecular Weight	Absolute Abundance	Relative Abundance
A	12140.22	392399	100.00
B	12161.99	201069	51.24
C	12179.13	160169	40.82
D	12095.52	52998	13.51

*** End of Report ***

p10

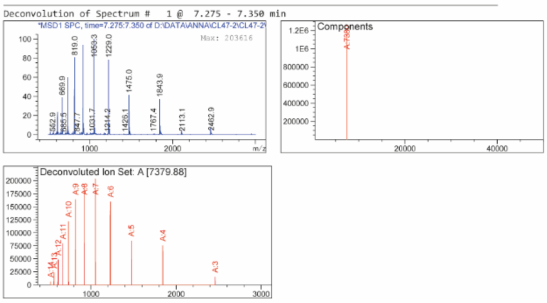

Component	Molecular Weight	Absolute Abundance	Relative Abundance
A	7379.88	1167977	100.00

*** End of Report ***

p11

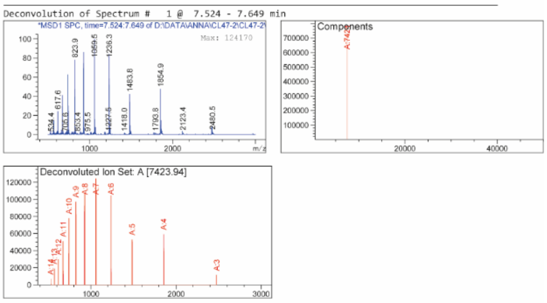

Component	Molecular Weight	Absolute Abundance	Relative Abundance
A	7423.94	732428	100.00

*** End of Report ***

p12

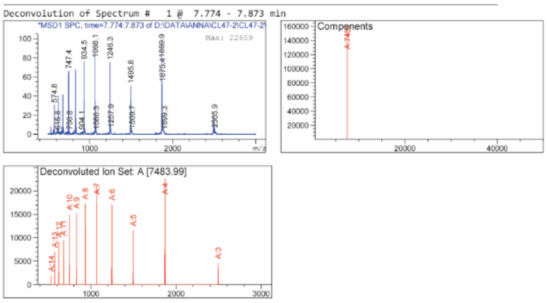

Component	Molecular Weight	Absolute Abundance	Relative Abundance
A	7483.99	150549	100.00

*** End of Report ***

Mixture: ORN-12a to ORN-12d and ORN-13

a)

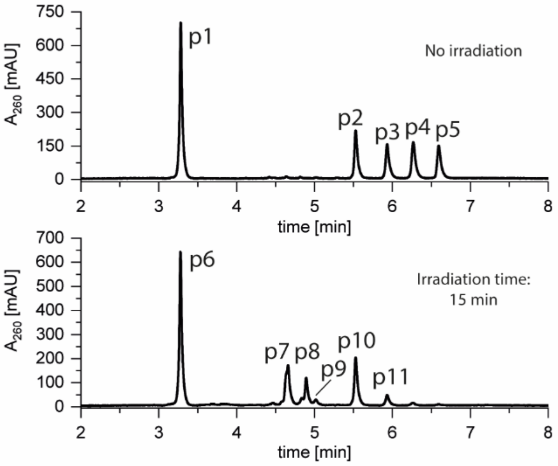

b)

Duplex	ORN	ORN mass	Calc. mass of the CL product
D25	ORN-12a	7175.5	12013.5
	ORN-13	4840.0	
D26	ORN-12b	7219.6	12057.6
	ORN-13	4840.0	
D27	ORN-12c	7263.6	12101.6
	ORN-13	4840.0	
D28	ORN-12d	7307.7	12145.7
	ORN-13	4840.0	

c) p1

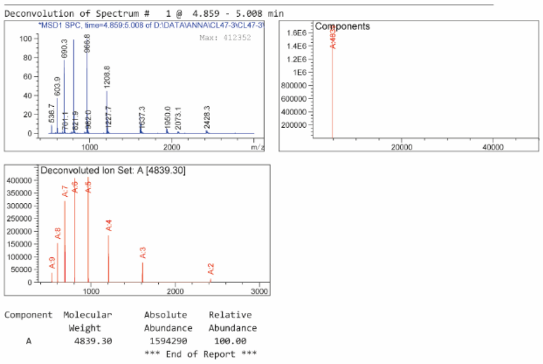

p2

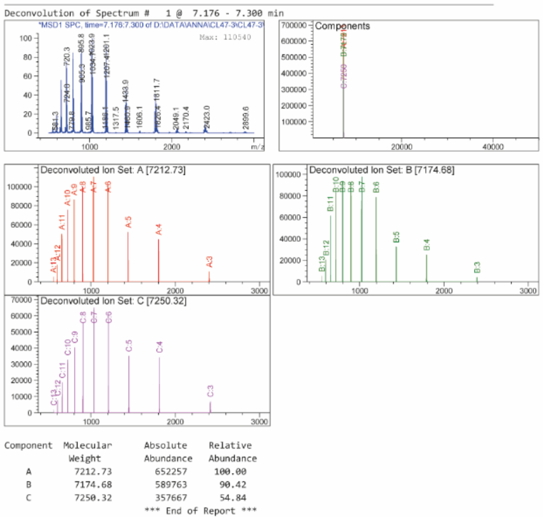

p3

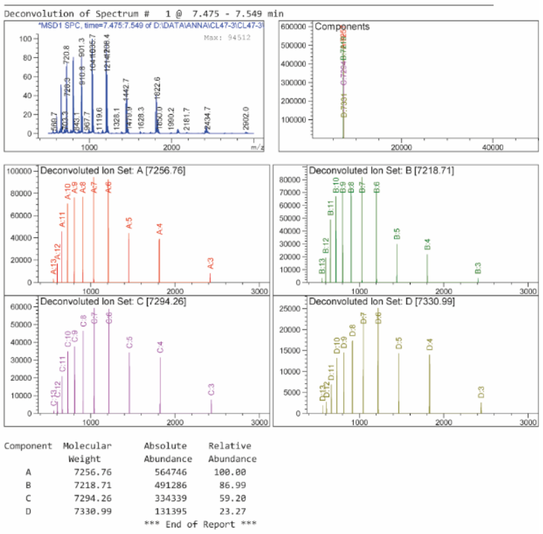

p4

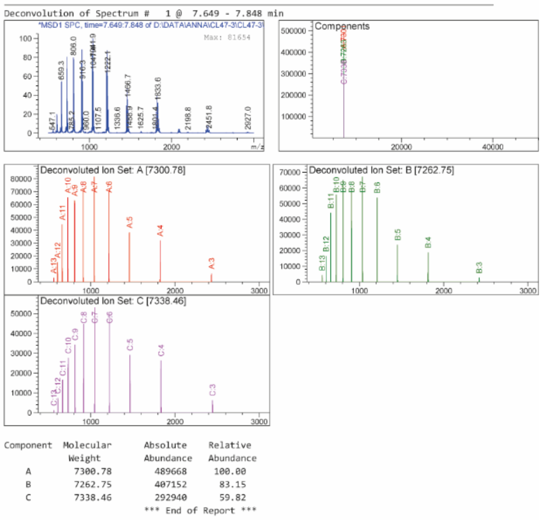

p5

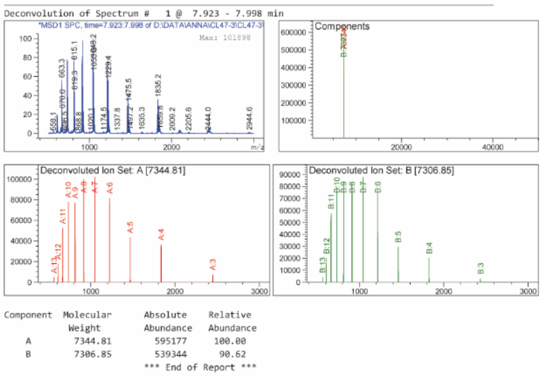

Mixture: ORN-12a to ORN-12d and ORN-13 continued

p6

Deconvolution of Spectrum # 1 @ 4.934 - 5.083 min

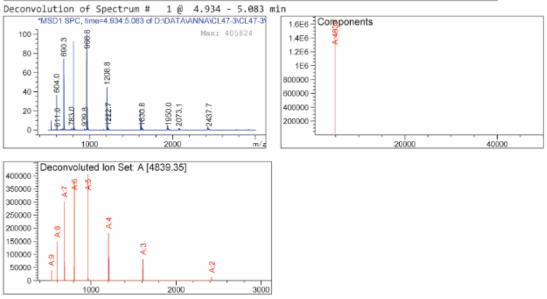

Component	Molecular Weight	Absolute Abundance	Relative Abundance
A	4839.35	1534746	100.00

*** End of Report ***

p7

Deconvolution of Spectrum # 1 @ 6.453 - 6.653 min

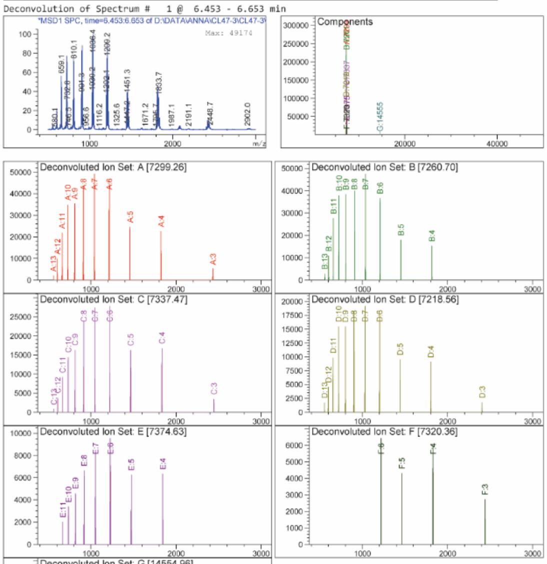

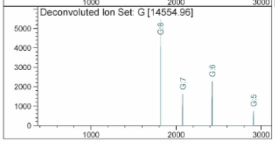

Component	Molecular Weight	Absolute Abundance	Relative Abundance
A	7299.26	291608	100.00
B	7260.70	273779	93.89
C	7337.47	159019	54.53
D	7218.56	122025	41.85
E	7374.63	46833	16.06
F	7320.36	18324	6.28
G	14554.96	9400	3.22

*** End of Report ***

p8

Deconvolution of Spectrum # 1 @ 6.603 - 7.001 min

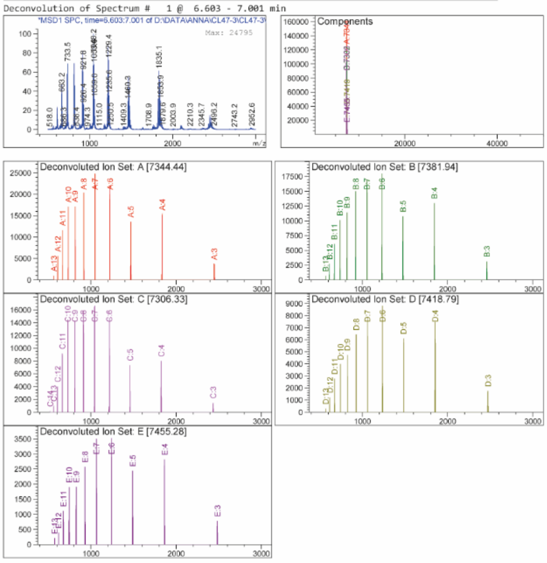

Component	Molecular Weight	Absolute Abundance	Relative Abundance
A	7344.44	150895	100.00
B	7381.94	106825	70.79
C	7306.33	106569	70.62
D	7418.79	50627	33.55
E	7455.28	20132	13.34

*** End of Report ***

p9

Deconvolution of Spectrum # 1 @ 6.678 - 6.777 min

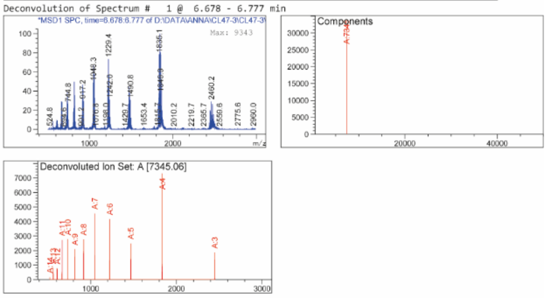

Component	Molecular Weight	Absolute Abundance	Relative Abundance
A	7345.06	31465	100.00

*** End of Report ***

p10

Deconvolution of Spectrum # 1 @ 7.101 - 7.250 min

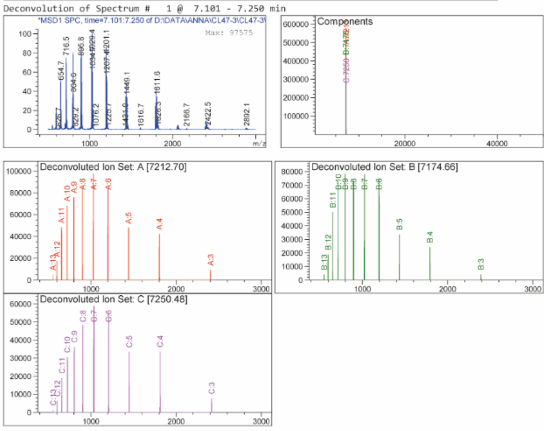

Component	Molecular Weight	Absolute Abundance	Relative Abundance
A	7212.70	577929	100.00
B	7174.66	509147	88.10
C	7250.48	327461	56.66

*** End of Report ***

p11

Deconvolution of Spectrum # 1 @ 7.375 - 7.549 min

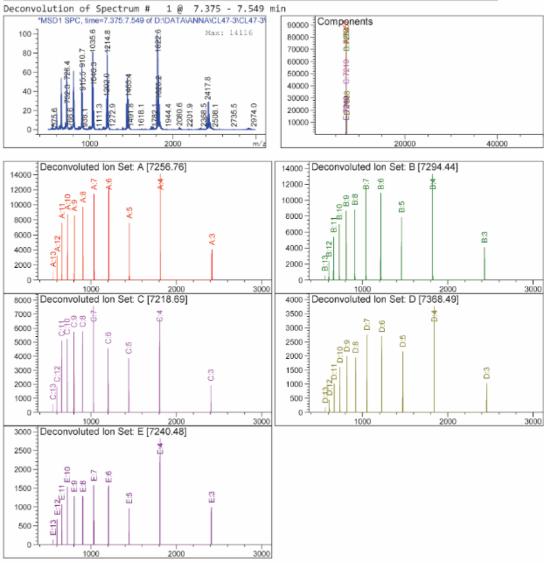

Component	Molecular Weight	Absolute Abundance	Relative Abundance
A	7256.76	87206	100.00
B	7294.44	80205	91.97
C	7218.69	48447	55.55
D	7368.49	18427	21.13
E	7240.48	13508	15.49

*** End of Report ***

[1] U. Pradere, A. Brunschweiger, L. F. Gebert, M. Lucic, M. Roos and J. Hall, *Angew Chem Int Ed Engl* **2013**, *52*, 12028-12032.
[2] D. Hei, *International Patent* **1996**.
[3] S. T. Isaacs, C.-K. J. Shen, J. E. Hearst and H. Rapoport, *Biochemistry* **1977**, *16*, 1058-1064.
[4] K. Shah, H. Wu and T. M. Rana, *Bioconjugate Chemistry* **1994**, *5*, 508-512.
[5] a) A. M. Jawalekar, N. Meeuwenoord, J. G. O. Cremers, H. S. Overkleeft, G. A. van der Marel, F. P. J. T. Rutjes and F. L. van Delft, *The Journal of Organic Chemistry* **2008**, *73*, 287-290; b) J. Imig, A. Brunschweiger, A. Brümmer, B. Guennewig, N. Mittal, S. Kishore, P. Tsikrika, A. P. Gerber, M. Zavolan and J. Hall, *Nat Chem Biol* **2015**, *11*, 107-114.
[6] U. Pradere and J. Hall, *Bioconjug Chem* **2016**, *27*, 681-687.
[7] J. A. Zagalak, M. Menzi, F. Schmich, H. Jahns, A. M. Dogar, F. Wullschleger, H. Towbin and J. Hall, *RNA* **2015**, *21*, 2132-2142.
